# Genetic architecture of primary biliary cholangitis: strong evidence for HLA and non-HLA risk loci

**DOI:** 10.3389/fimmu.2025.1600364

**Published:** 2025-08-29

**Authors:** Min Zhang, Liang Lyu, Liang Ge, Yizhou Wang, Dongqing Gu

**Affiliations:** ^1^ Department of Sleep and Psychology, Chongqing Health Center for Women and Children, Women and Children’s Hospital of Chongqing Medical University, Chongqing, China; ^2^ Department of College of Medical Informatics, Chongqing Medical University, Chongqing, China; ^3^ Department of Information, Chongqing Health Center for Women and Children (Women and Children’s Hospital of Chongqing Medical University), Chongqing, China; ^4^ Department of Laboratory of Infection and Immunity, West China School of Medical Sciences & Forensic Medicine, Sichuan University, Chengdu, China; ^5^ Department of Pathology, The Third Hospital of Mianyang, Sichuan Mental Health Center, Mianyang, Sichuan, China; ^6^ Department of Obstetrics and Gynecology, Chongqing Health Center for Women and Children (Women and Children’s Hospital of Chongqing Medical University), Chongqing, China

**Keywords:** primary biliary cholangitis, variants, meta-analysis, genetic architecture, cumulative evidence, functional annotation, phenome-wide analysis

## Abstract

**Background:**

Despite extensive genetic studies investigating primary biliary cholangitis (PBC), the mechanistic basis of risk-associated variants remains poorly understood. To address this gap, we performed a systematic evaluation of cumulative evidence linking genetic variants to PBC susceptibility.

**Methods:**

A comprehensive search was conducted to identify published studies on the association between genetic variants and PBC risk. Specifically, separate analyses were conducted for genome-wide association studies (GWASs) and candidate-gene association studies to address potential heterogeneity arising from differences in study design. Meta-analyses were performed to calculate pooled odds ratio (OR) and 95% confidence interval (CI) for the candidate-gene association studies. Significant associations were further graded using Venice criteria and false-positive report probability (FPRP) tests. Functional annotation, pathway enrichment, and phenome-wide analyses were performed to elucidate biological relevance.

**Results:**

Overall, we included 105 articles involving 71,031 cases and 140,499 controls. Meta-analyses were conducted for 70 variants across 33 genes. Among these, 44 variants were identified as significantly associated with PBC risk, comprising 30 HLA variants and 14 non-HLA variants. Separately, published GWAS have reported 115 significant variants. Nine variants (DQA1*0401, DQB1*0301, DQB1*0402, DQB1*0602, DRB1*08, DRB1*0803, DRB1*11, DRB1*1101, and rs7574865) were identified by both approaches. Additionally, meta-analyses of candidate-gene association studies provided strong evidence supporting the association of eight further variants (A*3303, B*4403, DPB1*0201, DQB1*0401, rs231725, rs231775, rs1544410, and rs9303277) with PBC at the genome-wide significance level (*P* < 5.0 × 10^-8^). Pathway analysis revealed significant enrichment of the mapped genes in immune cell regulation and immune response-regulating signaling pathways. Phenome-wide analyses further indicated that the missense variant rs231775 was significantly associated with thyroid problems and melanoma (*P*< 6.43×10^-5^).

**Conclusion:**

This study provides the most comprehensive synopsis to date of PBC’s genetic architecture, highlighting robust HLA and non-HLA risk loci.

**Systematic review registration:**

https:///www.crd.york.ac.uk/PROSPERO/view/CRD42021282146, identifier CRD42021282146.

## 1 Introduction

Primary biliary cholangitis (PBC), characterized by significant female predominance, is the most prevalent autoimmune liver disease (1). Individuals with PBC often experience symptoms that significantly impact their quality of life, including itching, fatigue, abdominal pain, and sicca complex (2). Untreated PBC is associated with an increased risk of cirrhosis and related complications, liver failure and even death (3). It is well known that genetic factors contribute to the pathogenesis of PBC. Several genome-wide association studies (GWASs) have identified variants in human leukocyte antigen (HLA) regions (e.g., DQB1*0301, DRB1*08, DRB1*1302) and outside HLA regions (non-HLA) that are associated with PBC susceptibility (4–7). Nevertheless, these loci together account for only 21% of the genetic causes of this disease (8).

Despite results from genome-wide association studies (GWASs) are prominent and increasingly available, candidate-gene association studies are still the most predominant type of research for identifying common risk alleles for PBC. Over the past decade, over 90 candidate-gene PBC association studies have been conducted, evaluating over 800 genetic loci in HLA region and non-HLA regions. While some of these genetic loci may indeed be linked to PBC risk, many others are false-positive associations that do not replicate in additional populations. The determination of whether these associations are validity typically involves a comprehensive examination of epidemiological evidence alongside biological plausibility, often through a meta-analysis which can enhance the statistical power and assess the replication and consistency of an association by consolidating data from multiple studies (9). In addition, following the guidelines developed by the Human Genome Epidemiology Network multidisciplinary workshop (10, 11), Venice criteria have been used to assess cumulative evidence of genetic associations (12–15). However, previous meta-analysis primarily focused on individual variants or those within a single gene (16–18), and no comprehensive field synopsis has been published to evaluate the cumulative evidence of associations between genetic variants and PBC risk so far.

In this study, we aimed to provide a comprehensive overview of the current understanding of the genetic architecture of PBC based on published literature. First, we conducted separate analyses for GWASs and candidate-gene association studies. For candidate-gene association studies, we performed a meta-analysis to comprehensively evaluate the association between genetic variants and PBC risk. We then evaluated the cumulative evidence for significant associations by combining Venice criteria and false-positive report probability (FPRP) tests. Finally, we conducted functional annotation, pathway analysis and phenome-wide analysis of potential pathogenic loci.

## 2 Materials and methods

The methodology for the meta-analysis followed the guidelines proposed by the Human Genome Epidemiology Network for a systematic review of genetic association studies and the Preferred Reporting Items for Systematic Reviews and Meta-Analyses statement (**Supplementary Tables S1**) (19, 20). The protocol was registered in the International Prospective Register of Systematic Reviews (CRD42021282146).

### 2.1 Literature search strategy and study eligibility

A comprehensive literature search of related studies was conducted using PubMed, Embase, and Web of Science (published on or before May 1, 2024), using the following keywords: “autoimmune liver disease OR primary biliary cholangitis OR primary biliary cirrhosis” AND “Genetic OR SNP OR polymorphism OR genotype OR variant OR allele OR mutation OR genome-wide association study OR GWAS.” The titles, abstracts, and full texts of the studies were reviewed as needed to identify all relevant articles. In addition, the reference lists of all included studies, reviews, and meta-analyses were manually screened for additional potential studies.

The inclusion criteria were as follows (1): original articles published in English (2); observational studies (3); investigating associations between genetic variants and risk of PBC; and (4) providing risk estimates [odds ratio (OR) and relative risk (RR]) and 95% confidence intervals (CIs) or data to calculate them. Exclusion criteria (1): participants complicated with other liver diseases (2); less than 50 cases and controls; and (3) reviews, abstracts, case reports, and letters. If several publications used the same or overlapping data, only the studies that reported results from the most recent or largest analysis was included. Two investigators (DG and YW) independently assessed the eligibility of each publication and any disagreements were discussed with the principal author (MZ).

### 2.2 Data extraction, preparation, and management

Two authors (DG and MZ) independently extracted data using a pre-designed collection sheet. The data included PMID, first author, publication year, study design, sample size of cases and controls, source of population, ethnicity, variants, gene, major and minor alleles, genotype and allele counts, risk estimates, and corresponding 95% CIs or *P*-value (for studies using multiple adjusted models, the most fully adjusted estimates were extracted).

### 2.3 Meta-analyses

To address potential heterogeneity arising from differences in study design, we conducted separate analyses for GWASs and candidate-gene association studies. For GWAS-derived data, we reported the SNP with the largest sample size at each locus within each ancestry group, along with its corresponding effect estimate, to avoid redundancy caused by linkage disequilibrium (LD) among SNPs at the same genomic region.

For candidate-gene association studies, we performed meta-analyses for variants with data available from at least three independent datasets. We calculated the pooled OR and 95%CIs using an additive genetic model. We meta-analyzed the associations of variants in human leukocyte antigen (*HLA*) gene with PBS risk and the associations of loci in non-HLA genes with PBS risk. Statistical heterogeneity among the studies was assessed using the Cochran Q statistic (*P* < 0.10 was considered statistically significant) and *I^2^* statistic (*I²* ≤ 25% represented mild heterogeneity, 25% - 50% represented moderate heterogeneity, and ≥ 50% represented large heterogeneity) (21). A random-effects model was used if *I^2^* ≥ 50%, while a fixed-effects model was used if *I^2^ <* 50%. For variants that showed a significant association with PBC, sensitivity analyses were performed by excluding the first published or positive study. Furthermore, we assessed potential publication bias using Begg’s test (22) and small-study bias using Egger’s test (23). In addition, we conducted subgroup meta-analyses stratified by Ethnicity (datasets ≥ 2 in either Asian or Caucasian populations). Between-subgroup heterogeneity was assessed using Cochran’s Q test, and *P* < 0.10 were considered indicative of significant ethnic heterogeneity. To explore potential sources of heterogeneity, we further performed meta-regression and subgroup analyses stratified by diagnostic criteria for PBC and genotyping method in meta-analyses with high heterogeneity.

### 2.4 Assessment of cumulative evidence

Associations with *P* < 0.05 in the primary meta-analyses were evaluated using the Venice criteria to assess epidemiological credibility. The detailed methods have been described in our previous research (24). Finally, epidemiological credibility was categorized as strong, moderate, or weak, based on the grade level of A, B, or C, according to three criteria: amount of evidence, replication, and protection from bias (10, 11). In addition, FPRP was calculated for these associations (25). Specifically, FPRP values of < 0.05, 0.20 - 0.05, and > 0.20 were considered strong, moderate, and weak evidence of a true association, respectively. We up-graded the cumulative evidence if the FPRP result was strong, and down-graded the cumulative evidence if the FPRP result was weak.

### 2.5 Functional annotation

To provide biological insights into the significant variants identified by our meta-analysis and previous GWASs, we mapped these SNPs to genes and conducted functional annotation using the Encyclopedia of DNA Elements (ENCODE) tool HaploReg v4.1 (26). To identify the tissues most relevant to the significant genes, we conducted Genotype-Tissue Expression (GTEx) tissue enrichment analysis based on 54 tissue types available from GTEx (version 8) using the functional mapping and annotation of genome-wide association studies (FUMA) GENE2FUNC process (27). Furthermore, we evaluated the enrichment of significantly mapped genes in Gene Ontology (GO) biological processes using the WebGestalt tool (28). We utilized the Benjamin-Hochberg procedure to correct for multiple testing and considered a false discovery rate (FDR) corrected *P*-value of less than 0.05 as a statistical difference.

### 2.6 Phenome-wide analyses

In addition, phenome-wide analyses were performed to estimate associations between the newly identified functional variants and 778 phenotypes from the UK Biobank, and summary data were generated using GeneATLAS (29). *P* values < 6.43×10^-5^ (0.05/778) were considered statistically significant after adjusting for multiple comparisons of variants and 778 phenotypes.

### 2.7 Statistical analysis

Statistical analysis was conducted using Stata version 15 (StataCorp, College Station, TX), and a two-tailed *P*-value of < 0.05 was considered statistically significant unless otherwise specified.

## 3 Results

### 3.1 Characteristics of the included studies

**Figure 1:**
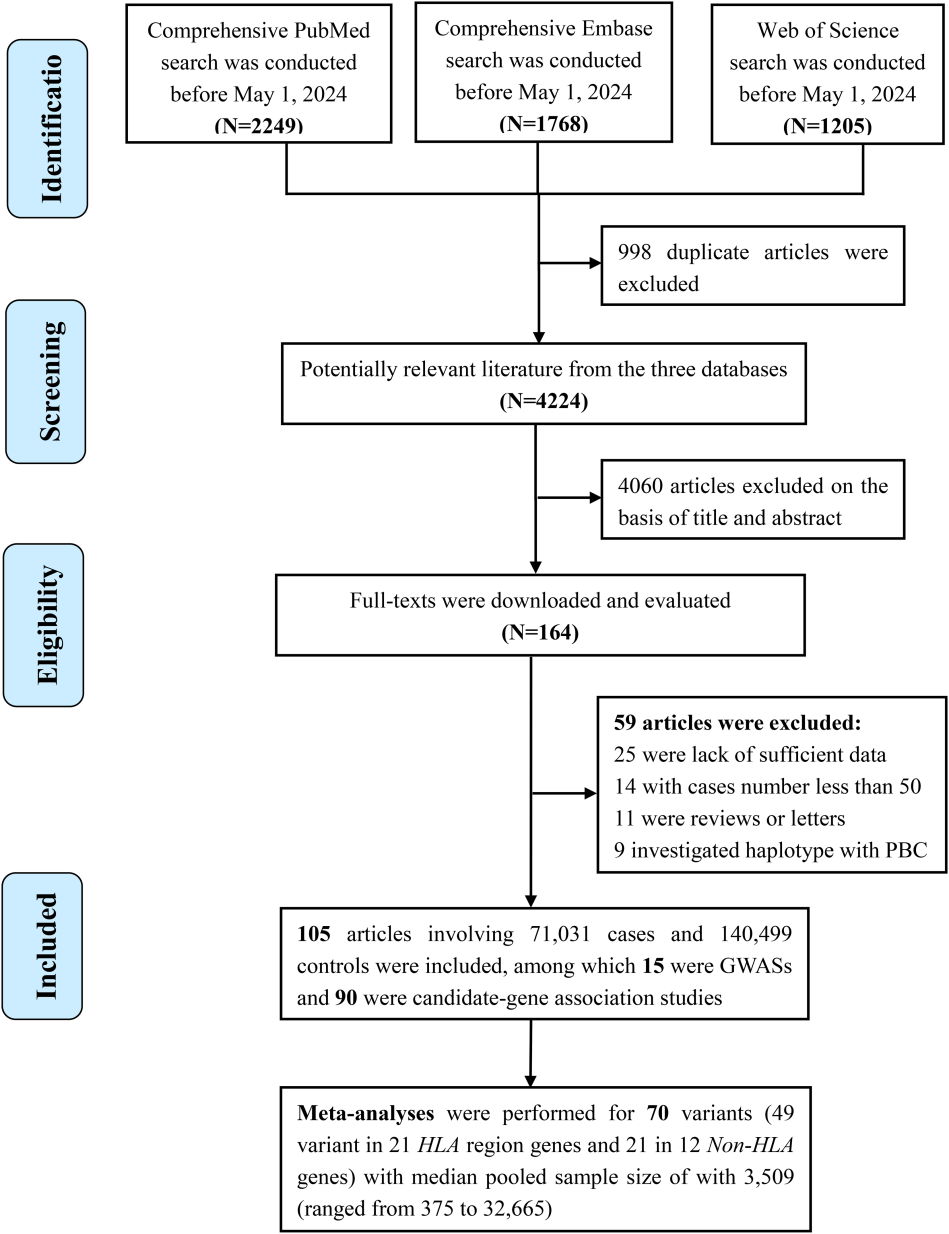
Flowchart of literature selection in the meta-analysis.

**Figure 2:**
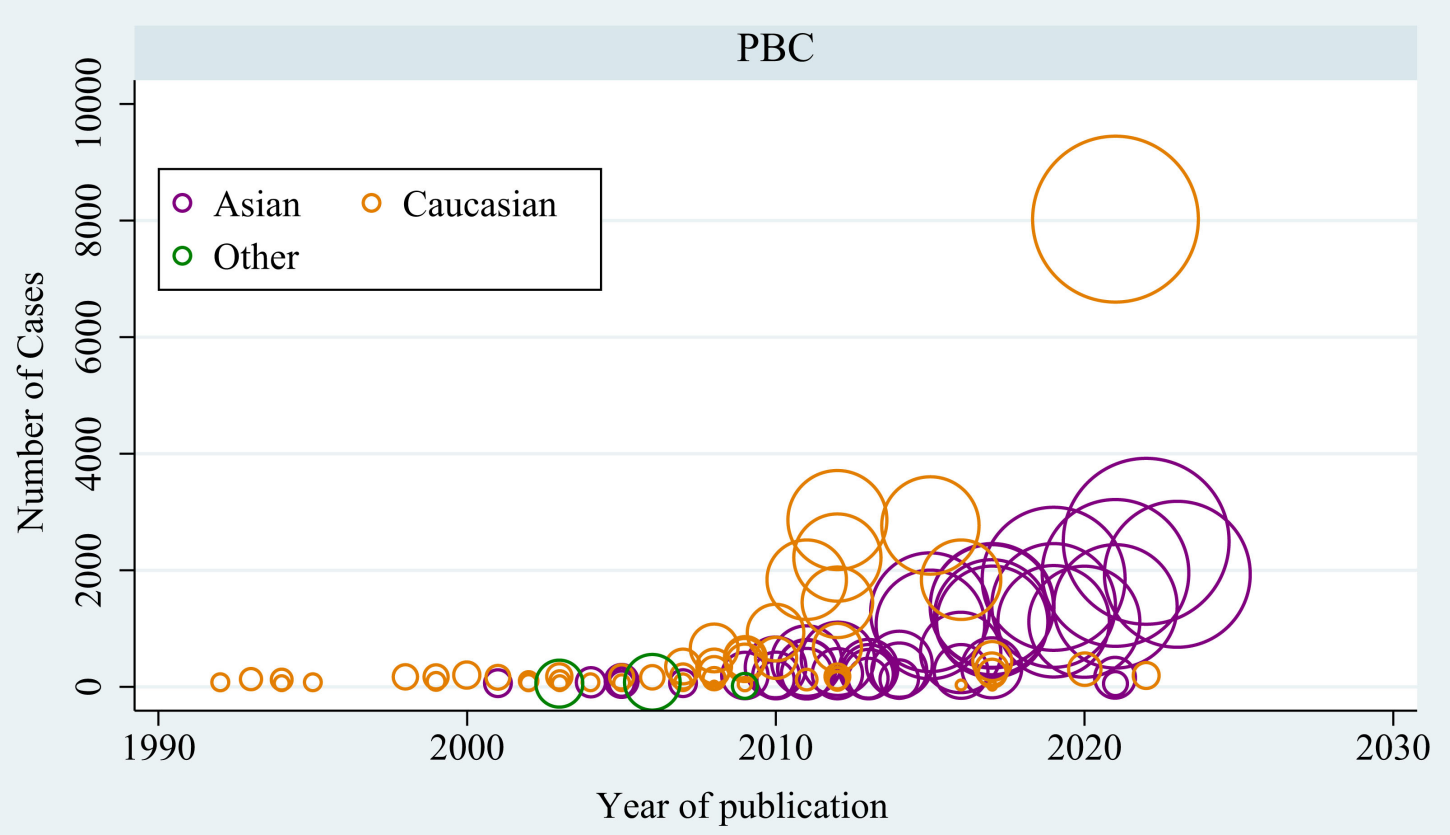
Characteristics of the included studies in the meta-analysis.

In total, 4,224 publications were screened after duplicates were excluded from the literature search (**Figure 1**). Ultimately, 105 articles involving 71,031 cases and 140,499 controls were included, and these articles investigated 1,341 variants located in 419 genes or chromosomal loci associated with risk of PBC. Most of these articles were conducted in Caucasians (n=62), followed by Asians (n=48) (**Figure 2**). The sample size ranged from 115 to 24,510 (median, 584), and the number of cases ranged from 16 to 8,061 (median, 232). Among these articles, 15 were GWASs (4, 6, 7, 30–41) (**Supplementary Table S2**) and 90 were candidate-gene association studies. Sixty-nine candidate-gene association studies explored the relationship between 302 variants in 122 non-HLA genes and the risk of PBC (**Supplementary Table S3**, **Supplementary Table S4**), whereas 23 studies investigated the association between 178 variants in *HLA* region and the risk of PBC (**Supplementary Table S5**, **Supplementary Table S6**).

### 3.2 Genome-wide significant associations in the GWASs

Fifteen GWASs identified 111 genome-wide significant SNPs across 55 loci associated with PBC, including 71 independent SNPs in 48 loci among Europeans, 26 independent SNPs in 17 loci among Chinese populations, and 16 independent SNPs in 10 loci among Japanese populations (**Table 1**). Among these SNPs, 40 (or loci in linkage disequilibrium with them) were replicated in more than two GWASs.

**Table 1:** Loci significantly associated with PBC identified by GWAS.

Chr	Locus	Gene	Country /Region	Variant	Cases/ Controls	OR (95%CI)	*P*	Linkage disequilibrium SNPs^†^	Confirmed by GWAS^§^
Non-HLA region									
1	1p13.1	*CD58*	Chinese	rs2300747	2029/6163	1.29 (1.20, 1.39)	1.11×10^-11^	rs10924106	(30)
1		*CD58*	Mix_European	rs10802191	8021/16489	0.81 (0.76, 0.87)	1.89×10^-8^		(31)
1	1p31	*IL12RB2*	Mix_European	rs6679356	8021/16489	1.55 (1.47, 1.63)	5.84×10^-64^	rs3790567, rs72678531, rs17129789, rs3790565	(4, 31–36)
1	1p36.32	*TNFSF14*	Mix_European	rs867436	8021/16489	1.14 (1.09, 1.20)	5.67×10^-9^	rs10752747, rs3748816	(31, 34, 36)
1	1q23.1	*FCRL3*	Mix_European	rs945635	8021/16489	0.89 (0.85, 0.92)	2.93×10^-8^		(31)
1	1q31.3	*DENND1B*	Mix_European	rs12123169	8021/16489	1.24 (1.18, 1.31)	2.78×10^-18^	rs2488393, rs17641524, rs12134279	(6, 31, 32, 34)
1	1q32.1	*INAVA*	Mix_European	rs55734382	8021/16489	0.87 (0.83, 0.91)	1.15×10^-9^		(31)
2	2p23	*DNMT3A*	Mix_European	rs34655300	8021/16489	1.15 (1.10, 1.20)	4.75×10^-10^		(31)
2		*LBH*	Mix_European	rs4952108	2764/10475	1.28 (1.17, 1.40)	5.05×10^-8^		(6)
2	2q21.3	*TMEM163*	Mix_European	rs859767	8021/16489	0.87 (0.83, 0.91)	1.51×10^-9^		(31)
2	2q32	*STAT4*	Chinese	rs10168266	2029/6163	1.31 (1.22, 1.41)	2.61×10^-13^		(30)
2		*STAT4*	Japanese	rs11889341	2181/2699	1.33 (1.21, 1.45)	3.32×10^-10^		(37)
2		*NAB1*	Mix_European	rs3771317	8021/16489	1.34 (1.26, 1.42)	4.18×10^-22^	rs10931468	(6, 31, 34)
2		*STAT4*	British	rs3024921	2861/8514	1.62 (1.45, 1.80)	2.21×10^-18^		(32, 33)
2		*STAT4*	British	rs7574865	2861/8514	1.31 (1.22, 1.40)	1.45×10^-14^		(32)
2	2q33.2	*CD28/CTLA4*	Chinese	rs4675369	2029/6163	1.31 (1.22, 1.41)	2.61×10^-13^	rs7599230	(30)
2	2q36.3	*IL18RAP*	Mix_European	rs4973341	4688/12221	0.82 (0.74, 0.90)	2.34×10^-10^		(6)
3	3p24.2	*RARB*	Mix_European	rs6550965	8021/16489	1.18 (1.13, 1.23)	1.27×10^-13^		(31)
3	3p24.3	*PLCL2*	Mix_European	rs9876137	8021/16489	1.15 (1.11, 1.21)	5.93×10^-11^	rs1372072	(6, 31, 34)
3	3q13.33	*CD80*	Japanese	rs9855065	2181/2699	0.72 (0.66, 0.79)	1.51×10^-12^	rs57271503, rs2293370	(37–40)
3		*CD80*	Chinese	rs3732421	2029/6163	0.74 (0.68, 0.80)	3.79×10^-13^		(30)
3		*CD80*	Mix_European	rs2293370	8021/16489	0.74 (0.70, 0.78)	6.33×10^-25^	rs1131265	(6, 31–34)
3	3q25.33	*IL12A*	Chinese	rs582537	2029/6163	0.75 (0.69, 0.82)	6.44×10^-11^		(30)
3		*IL12A*	Mix_European	rs589446	8021/16489	0.70 (0.67, 0.73)	6.15×10^-58^	rs6441286, rs9877910, rs2366643, rs668998, rs485499, rs574808	(4, 6, 31–36)
3		*IL12A*	British	rs80014155	2861/8514	3.44 (2.39, 4.94)	2.55×10^-11^		(32)
3		*IL12A*	British	rs62270414	2861/8514	1.41 (1.30, 1.53)	1.36×10^-16^		(32)
4	4p16.3	*GAK*	Mix_European	rs11724804	4556/12990	1.22 (1.12, 1.33)	9.01×10^-12^		(6)
4	4q24	*NFKB1*	Chinese	rs1598856	2029/6163	1.26 (1.17, 1.35)	2.44×10^-10^		(30)
4		*MANBA*	Japanese	rs223492	2181/2699	1.38 (1.27, 1.50)	1.87×10^-13^		(37)
4		*NFKB1*	Japanese	rs17033015	1855/1719	1.35 (1.23, 1.49)	9.00×10^-10^		(38)
4		*NFKB1*	Mix_European	rs7674640	8021/16489	0.81 (0.77, 0.84)	9.40×10^-23^	rs7665090, rs1054037	(6, 31, 32, 34)
4		*TET2*	Mix_European	rs7663401	8021/16489	0.88 (0.84, 0.92)	4.30×10^-8^		(31)
4	4q27	*IL21*	Chinese	rs925550	2029/6163	1.31 (1.21, 1.40)	3.95×10^-13^	rs17005934	(30)
5	5p13.2	*IL7R*	Japanese	rs11406102	2181/2699	0.70 (0.62, 0.78)	1.48×10^-9^		(37)
5		*IL7R*	Japanese	rs12697352	1855/1719	0.68 (0.60, 0.77)	2.00×10^-9^	rs6897932, rs6890853	(38–40)
5		*IL7R*	Mix_European	rs35467801	8021/16489	0.80 (0.76, 0.84)	3.25×10^-19^		(31)
5		*IL7R*	British	rs6871748	2861/8514	1.30 (1.21, 1.40)	1.77×10^-12^	rs860413	(6, 32, 34)
5	5q21.1	*PAM*	Mix_European	rs526231	6480/14736	0.87 (0.81, 0.93)	1.14×10^-8^		(6)
5	5q33.3	*IL12B/RNF145*	Mix_European	rs2546890	8021/16489	0.87 (0.83, 0.90)	5.93×10^-11^		(6, 31)
7	7p14.1	*ELMO1*	Mix_European	rs60600003	8021/16489	1.29 (1.20, 1.38)	4.88×10^-13^	rs7805218	(31, 34)
7	7p21.1	*ITGB8*	Mix_European	rs7805218	8021/16489	1.14 (1.09, 1.19)	2.04×10^-8^		(31)
7	7q32.1	*IRF5/TNPO3*	Mix_European	rs12531711	8021/16489	1.52 (1.43, 1.62)	8.10×10^-42^	rs10488631, rs35188261	(4, 6, 31–33, 35, 36)
7		*IRF5/TNPO3*	British	rs3807307	2861/8514	1.22 (1.14, 1.30)	2.94×10^-9^		(32)
7	7q34	*ZC3HAV1L*	Mix_European	rs370193557	8021/16489	1.13 (1.08, 1.18)	2.93×10^-^8		(31)
9	9q22.33	*TRIM14*	Mix_European	rs11390003	8021/16489	0.86 (0.82, 0.91)	3.42×10^-^8		(31)
9	9q32	*TNFSF8*	Chinese	rs4979467	2029/6163	1.53 (1.42, 1.64)	5.61×10^-31^		(30)
9		*TNFSF15*	Japanese	rs4979462	2181/2699	1.62 (1.49, 1.76)	4.49×10^-31^		(37–40)
10	10q11.23	*WDFY4*	Mix_European	rs7097397	8021/16489	0.87 (0.83, 0.91)	3.83×10^-10^		(31)
11	11p15.5	*IRF7*	Mix_European	rs58523027	8021/16489	0.88 (0.85, 0.92)	2.26×10^-8^		(31)
11	11q13.1	*CCDC88B*	Mix_European	rs11601860	8021/16489	0.86 (0.83, 0.90)	2.18×10^-10^	rs538147, rs510372	(6, 31, 34)
11	11q23.1	*POU2AF1*	Mix_European	rs12419634	8021/16489	0.88 (0.84, 0.92)	5.95×10^-9^		(31)
11		*POU2AF1*	Japanese	rs4938534	1381/1505	1.35 (1.22, 1.50)	1.49×10^-8^		(39, 40)
11	11q23.3	*CXCR/DDX6*	Chinese	rs77871618	2029/6163	1.40 (1.28, 1.53)	1.44×10^-13^		(30)
11		*CXCR/DDX6*	Mix_European	rs201150316	8021/16489	0.69 (0.65, 0.73)	9.06×10^-35^	rs7117261, rs80065107, rs6421571	(6, 31–34)
12	12p13.31	*TNFRSF1A*	Mix_European	rs1800693	8021/16489	1.20 (1.15, 1.25)	2.80×10^-16^	rs11064157	(6, 31, 32, 34)
12		*NFKB1*	Chinese	rs4149576	2029/6163	1.37 (1.23, 1.52)	5.56×10^-9^		(30)
12	12q24.12	*SH2B3/ATXN2*	Mix_European	rs35350651	8021/16489	0.83 (0.79, 0.86)	9.44×10^-20^	rs11065979, rs11065987	(6, 31, 32)
13	13q14.11	*TNFSF11*	Mix_European	rs9533122	8021/16489	0.86 (0.82, 0.89)	1.85×10^-12^	rs3862738	(31, 33)
13	13q14.2	*DLEU1*	Mix_European	rs9591325	8021/16489	0.64 (0.58, 0.70)	1.57×10^-19^		(6, 31)
14	14q24.1	*RAD51B*	Mix_European	rs3784099	8021/16489	0.82 (0.78, 0.86)	2.71×10^-17^	rs911263	(6, 31, 32, 34)
14	14q32.12	*RIN3*	Mix_European	rs72699866	8021/16489	0.82 (0.78, 0.87)	1.77×10^-11^		(31)
14	14q32.32	*TNFAIP2*	Mix_European	rs59643720	8021/16489	1.37 (1.31, 1.44)	1.37×10^-39^	rs8017161, rs2297067	(6, 31, 34)
15	15q25.1	*IL16*	Chinese	rs11556218	2029/6163	1.29 (1.18, 1.41)	2.08×10^-8^		(30)
16		*PRKCB*	Japanese	rs7404928	1893/8017	1.25 (1.09, 1.43)	4.13×10^-9^		(39)
16	16p12.1	*IL21R*	Mix_European	rs1119132	8021/16489	0.82 (0.77, 0.87)	7.67×10^-10^		(31)
16		*IL4R/IL21R*	Chinese	rs2189521	2029/6163	0.71 (0.66, 0.78)	9.23×10^-16^	rs10852316	(30)
16	16p13.13	*CLEC16A*	Mix_European	rs9652601	8021/16489	0.79 (0.75, 0.82)	1.52×10^-23^	rs12708715, rs12924729	(6, 31, 32, 34)
16		*SOCS1/RMI2*	British	rs1646019	2861/8514	1.31 (1.23, 141)	6.72×10^-15^	rs413024	(31, 32)
16		*SOCS1/RMI2*	British	rs80073729	2861/8514	2.96 (2.02, 4.33)	2.42×10^-8^		(32)
16	16q21	*CCDC113*	Chinese	rs2550374	2029/6163	0.81 (0.76, 0.87)	9.91×10^-10^		(30)
16	16q22.1	*DPEP3*	Mix_European	rs79577483	8021/16489	1.24 (1.16, 1.31)	7.99×10^-12^		(31)
16	16q24.1	*IRF8*	Mix_European	rs11117432	8021/16489	0.76 (0.72, 0.80)	4.93×10^-24^		(31, 32, 34)
17	17q12	*IKZF3*	Mix_European	rs33938760	8021/16489	0.77 (0.74, 0.80)	1.83×10^-32^	rs9303277, rs907092, rs907091, rs8067378, rs7208487, rs2305480, rs12924729	(4, 6, 31–36)
17		*IKZF3*	Chinese	rs9635726	2029/6163	1.37 (1.27, 1.48)	7.36×10^-16^		(30)
17		*ZPBP2*	Japanese	rs200216139	2181/2699	1.48 (1.34, 1.62)	3.43×10^-16^		(37)
17		*IKZF3*	Japanese	rs4795395	1855/1719	1.42 (1.29, 1.57)	4.00×10^-12^	rs9303277	(38–40)
17	17q21.31	*MAPT*	Mix_European	rs17564829	8021/16489	0.84 (0.80, 0.89)	3.71×10^-11^		(31, 32)
18	18q22.2	*CD226*	Mix_European	rs1808094	8021/16489	1.14 (1.09, 1.18)	1.09×10^-9^		(31)
18	18p11.21	*PTPN2*	Japanese	rs8098858	2181/2699	1.34 (1.21, 1.48)	2.56×10^-8^		(37)
19	19p13.2	*TYK2*	British	rs34536443	2861/8514	1.91 (1.59, 2.28)	1.96×10^-12^		(32)
19		*TYK2*	Mix_European	rs2304256	8021/16489	0.81 (0.78, 0.85)	1.32×10^-17^		(6, 31)
19	19p13.3	*ARID3A*	Chinese	rs10415976	2029/6163	0.77 (0.72, 0.84)	3.00×10^-11^	rs10414193	
19	19q13.33	*SPIB*	Mix_European	rs3745516	8021/16489	1.32 (1.25, 1.38)	3.45×10^-30^		(6, 31, 34, 35)
22	22q13.1	*RPL3/SYNGR1*	Chinese	rs137603	2029/6163	0.73 (0.65, 0.81)	2.07×10^-8^		(30)
22		*SYNGR1*	Mix_European	rs137687	8021/16489	0.80 (0.77, 0.84)	3.80×10^-23^	rs2267407, rs715505, rs2069235, rs968451	(6, 31–34)
HLA region									
6	6p21	*HLA-DRB1*	Chinese	rs16822805	1126/1770	1.70 (1.51, 1.92)	4.75×10^-18^		(7)
6		*HLA-DRB1*	Chinese	rs17886882	1126/1770	0.58 (0.52, 0.65)	1.08×10^-21^		(7)
6		*HLA-DRA*	Chinese	rs9268644	2029/6163	0.51 (0.45, 0.57)	7.83×10^-31^		(30)
6		*HLA-DRA*	Chinese	rs9501251	2029/6163	2.01 (1.76, 2.32)	2.10×10^-22^		(30)
6		*HLA-DQB1*	Japanese	rs9275175	487/476	1.94 (1.62, 2.33)	8.30×10^-13^		(40)
6		*HLA-DRA*	Japanese	rs9268641	2181/2699	0.46 (0.41, 0.52)	1.49×10^-37^	rs3129887	(37, 39)
6		*BTNL2*	Italian	rs116348417	676/1440	0.66 (0.57, 0.77)	4.90×10^-8^	rs3135363	(4, 41)
6		*HLA-DQB1/HLA-DQA2*	Mix_European	rs7775055	2216/5594	3.71 (3.00, 4.59)	1.27×10^-33^	rs115721871, rs4246055, rs114327274, rs2395148	(4, 33, 35, 41)
6		*HLA-DQB1/HLA-DQA2*	Mix_European	rs7774434	8021/16489	1.60 (1.53, 1.67)	2.91×10^-101^	rs114432443, rs114183935, rs9275424, rs9275390, rs2856683, rs7775228, rs9275312, rs660895, rs3806156, rs114796881, rs116493712	(4, 6, 31, 32, 34, 35, 41)
6		*HLA‐DPB1*	Mix_European	rs9277535	1351/4700	1.51 (1.37, 1.66)	3.98×10^-17^	rs2855430	(4, 36)
6	6q23.3	*TNFAIP3*	Mix_European	rs2327832	8021/16489	1.17 (1.12, 1.23)	1.19×10^-10^	rs6933404	(6, 31)
6		*HLA-DQB1*	Chinese	DQB1*03:01	1126/1770	0.52 (0.45, 0.61)	3.57×10^-17^		(7)
6		*HLA-DQB1*	British	DQB1*03:01	2861/8514	0.70 (0.64, 0.77)	6.48×10^-14^		(32)
6		*HLA-DQB1*	Italian	DQB1*03:01	676/1440	0.61 (0.52, 0.72)	6.10×10^-9^		(41)
6		*HLA-DQB1*	British	DQB1*06:02	2861/8514	0.64 (0.57, 0.72)	2.32×10^-15^		(32)
6		*HLA-DQB1*	Italian	DQB1*04:02	676/1440	3.16 (2.22, 4.49)	1.40×10^-10^		(41)
6		*HLA-DQA1*	British	DQA1*04:01	2861/8514	3.06 (2.62, 3.58)	5.90×10^-45^		(32)
6		*HLA-DQA1*	Italian	DQA1*04:01	676/1440	0.32 (0.23, 0.45)	1.90×10^-10^		(41)
6		*HLA-DQA1*	Chinese	DQA1*05:05	1126/1770	0.52 (0.43, 0.64)	1.15×10^-10^		(7)
6		*HLA-DPB1*	Chinese	DPB1*17:01	1126/1770	2.43 (1.88, 3.13)	8.62×10^-12^		(7)
6		*HLA-DRB1*	Italian	DRB1*11	676/1440	0.55 (0.46, 0.66)	1.40×10^-10^		(41)
6		*HLA-DRB1*	Chinese	DRB1*11:01	1126/1770	0.47 (0.36, 0.62)	1.39×10^-8^		(7)
6		*HLA-DRB1*	Italian	DRB1*08	676/1440	3.22 (2.29, 4.53)	1.60×10^-11^		(41)
6		*HLA-DRB1*	Chinese	DRB1*08:03	1126/1770	1.64 (1.38, 1.95)	2.04×10^-8^		(7)
6		*HLA-DRB1*	British	DRB1*04:04	2861/8514	1.57 (1.36, 1.82)	1.22×10^-9^		(32)
6		*HLA-DPA1*	Chinese	DPA1*01:03	1126/1770	0.71 (0.64, 0.80)	1.78×10^-9^		(7)

**^†^** Identified by GWAS; ^§^Confirmed by GWAS in the reference.

GWASs, genome-wide association studies; OR, odds ratio; CI, confidence interval; HLA, human leukocyte antigen.

### 3.3 Results of the meta-analysis in the candidate-gene association studies

Meta-analyses were performed for 70 associations for variants (49 variant in 21 *HLA* region genes and 21 in 12 non-HLA genes) with available data from at least three independent sources. The median pooled sample size of the 70 meta-analyses was 3,509 (ranged from 375 to 32,665).

30 variants within six HLA genes (*HLA-A*, *HLA-B*, *HLA-DQA1*, *HLA-DQB1*, *HLA-DRB1*, *HLA-DPB1*) were found to be significantly associated with the risk of PBC (**Table 2**). Strong associations (OR > 2 or < 0.5) with PBC-risk were identified for 11 variants, with the strongest positive association was observed for DRB1*0801 (OR=3.11, 95% CI=1.59-6.08, *P*=9.16×10-4) and negative association for DQB1*0604 (OR=0.31, 95% CI=0.20-0.48, *P*=1.42×10-7). Ten variants (A*3303, B*4403, DPB1*0201, DPB1*0501, DQB1*0301, DQB1*0401, DQB1*0601, DRB1*08, DRB1*0803, DRB1*1101) had associations with PBC risk at genome-wide significance level (*P* < 5.0×10-8), among which DQB1*0301, DRB1*08, DRB1*0803 and DRB1*1101 were previously identified genome-wide significant risk loci (**Table 1**). No significant associations were found for another 19 variants in *HLA* region (**Supplementary Table S7**). Subgroup analyses show that among the 21 variants eligible for subgroup analysis, 11 (52.4%) displayed significant between-subgroup heterogeneity (*P* for subgroup heterogeneity < 0.1).

**Table 2:** Variants in *HLA* genes significantly associated with risk of primary biliary cholangitis in meta-analysis.

Variant	Ethnicity	Data sets	Cases/ Controls	Risk estimates		Heterogeneity		P for Inter action	Venice criteria grade	FPRP	Cumulative evidence of association^§^
				OR (95%CI)	*P*	*І* ^2^	*P*				
A*33:03	Asian^†^	3	3757/3372	0.42 (0.36, 0.49)	6.68×10^-27^	0.00%	0.92		AAA	<0.001	Strong
B*44:03	Asian^†^	3	3757/3372	0.33 (0.28, 0.39)	9.48×10^-37^	0.00%	0.89		AAA	<0.001	Strong
DPB1*02:01	All ancestries	3	3610/2952	0.70 (0.63, 0.76)	2.22×10^-14^	0.00%	0.52	0.29	AAA	<0.001	Strong
	Asian	2	3528/3849	0.69 (0.63, 0.76)	1.26×10^-14^	0.00%	0.67				
DQB1*03:01	All ancestries	8	4549/4765	0.54 (0.47, 0.61)	1.29×10^-20^	12.70%	0.33	0.06	AAA	<0.001	Strong
	Asian	4	3902/3872	0.51 (0.44, 0.58)	6.09×10^-25^	0.00%	0.66				
	Caucasian	4	647/893	0.66 (0.52, 0.85)	1.00×10^-03^	0.00%	0.4				
DQB1*04:01	Asian^†^	4	3902/3807	1.43 (1.27, 1.62)	9.64×10^-09^	21.60%	0.28		AAA	<0.001	Strong
DRB1*08	All ancestries	9	2179/5040	2.88 (2.40, 3.46)	1.02×10^-29^	0.00%	0.68	1.00	AAA	<0.001	Strong
	Caucasian	8	1734/3996	2.88 (2.36, 3.51)	9.81×10^-26^	0.00%	0.56				
DRB1*08:03	Asian^†^	5	3954/3998	1.87 (1.63, 2.14)	1.90×10^-19^	17.20%	0.31		AAA	<0.001	Strong
DRB1*11:01	Asian^†^	5	3088/3060	0.42 (0.31, 0.57)	1.88×10^-8^	0.00%	0.74		AAA	<0.001	Strong
DRB1*14:03	Asian^†^	3	3573/3349	0.27 (0.17, 0.44)	1.44×10^-7^	0.00%	0.65		AAA	<0.001	Strong
DPB1*05:01	All ancestries	3	3610/2952	1.40 (1.29, 1.51)	5.23×10^-16^	0.00%	0.71	0.46	AAC	<0.001	Moderate
	Asian	2	3528/3849	1.40 (1.29, 1.52)	4.02×10^-16^	0.00%	0.71				
DQA1*04:01	All ancestries	5	3056/2467	2.60 (1.71, 3.95)	7.54×10^-6^	44.10%	0.13	0.01	ABC	<0.001	Moderate
	Caucasian	4	728/814	3.36 (2.17, 5.22)	6.48×10^-8^	0.00%	0.83				
DQB1*04:02	All ancestries	8	4511/4121	2.26 (1.63, 3.15)	1.20×10^-6^	55.60%	0.03	<0.001	ACC	<0.001	Moderate
	Asian	3	3673/3349	1.58 (1.32, 1.90)	8.18×10^-7^	0.00%	0.72				
	Caucasian	4	838/772	3.52 (2.38, 5.19)	2.48×10^-10^	0.00%	0.78				
DQB1*06:01	All ancestries	6	4057/4434	1.52 (1.40, 1.65)	5.73×10^-23^	0.00%	0.49	<0.001	AAC	<0.001	Moderate
	Asian	4	3902/3872	1.54 (1.39, 1.71)	3.38×10^-16^	18.90%	0.3				
	Caucasian	2	155/562	2.22 (0.70, 7.05)	0.18	0.00%	0.56				
DQB1*06:02	All ancestries	6	3021/3340	0.68 (0.52, 0.88)	4.00×10^-3^	50.00%	0.08	0.78	ABA	0.15	Moderate
	Asian	3	2702/2676	0.70 (0.48, 1.03)	0.07	71.40%	0.03				
	Caucasian	3	319/664	0.65 (0.40, 1.03)	0.07	32.60%	0.23				
DQB1*06:04	All ancestries	5	3912/3934	0.31 (0.20, 0.48)	1.42×10^-7^	64.20%	0.03	<0.001	ACA	<0.001	Moderate
	Asian	3	3757/3372	0.24 (0.19, 0.32)	3.32×10^-25^	29.60%	0.24				
	Caucasian	2	155/562	0.86 (0.36, 2.08)	0.74	0.00%	0.34				
DRB1*04:05	Asian^†^	5	3954/3998	1.40 (1.22, 1.60)	7.97×10^-7^	25.20%	0.25		ABC	0	Moderate
DRB1*07:01	Asian^†^	3	531/884	1.84 (1.31, 2.57)	3.97×10^-4^	0.00%	0.43		BAC	0.01	Moderate
DRB1*08:01	All ancestries	3	570/741	3.11 (1.59, 6.08)	9.16×10^-4^	0.00%	0.47	0.93	BAA	0.15	Moderate
	Caucasian	2	236/483	3.34 (1.39, 8.00)	7.00×10^-3^	33.10%	0.22				
DRB1*08:02	All ancestries	4	2879/2792	1.48 (1.22, 1.80)	9.19×10^-5^	0.00%	0.87	0.56	AAC	0	Moderate
	Asian	3	2807/2411	1.48 (1.21, 1.80)	1.04×10^-4^	0.00%	0.83				
DRB1*12:01	Asian^†^	4	2859/2537	0.66 (0.52, 0.83)	4.37×10^-4^	0.00%	0.86		AAC	0.01	Moderate
DRB1*13:02	All ancestries	6	4308/4511	0.38 (0.25, 0.57)	2.72×10^-6^	84.90%	<0.001	<0.001	ACA	<0.001	Moderate
DQA1*01:02	All ancestries	6	4256/3663	0.61 (0.42, 0.90)	0.01	85.10%	<0.001	0.01	ACC	0.32	Weak
	Asian	2	3528/2849	0.42 (0.34, 0.51)	7.43×10^-17^	51.90%	0.15				
	Caucasian	4	728/814	0.80 (0.50, 1.30)	0.38	63.90%	0.04				
DPB1*04:01	All ancestries	3	3610/2952	0.41 (0.18, 0.95)	0.04	92.10%	<0.001	<0.001	ACC	0.57	Weak
	Asian	2	3528/3849	0.25 (0.20, 0.32)	3.68×10^-30^	0.00%	0.75				
DRB1*11	Caucasian^†^	8	1619/3728	0.58 (0.40, 0.85)	5.00×10^-3^	64.40%	0.01		ACA	0.11	Weak
DRB1*12:02	Asian^†^	3	531/884	0.50 (0.31, 0.81)	4.00×10^-3^	0.00%	0.55		BAC	0.14	Weak
DRB1*13	Caucasian^†^	9	1756/4053	0.66 (0.46, 0.93)	0.02	55.50%	0.02		ACA	0.41	Weak
	Asian	5	4236/4130	0.30 (0.24, 0.39)	8.31×10^-22^	31.50%	0.21				
DRB1*14	All ancestries	6	1252/3808	1.68 (1.03, 2.74)	0.04	61.20%	0.02	0.92	ACC	0.48	Weak
	Caucasian	5	1107/3308	1.72 (0.93, 3.18)	0.09	68.10%	0.01				
DRB1*14:05	Asian^†^	3	531/884	1.80 (1.01, 3.22)	0.05	37.60%	0.2		BBC	0.64	Weak
DRB1*15	Caucasian^†^	5	722/1312	0.73 (0.55, 0.98)	0.03	11.40%	0.34		BAA	0.39	Weak
DRB1*15:01	All ancestries	6	3095/3020	0.70 (0.54, 0.89)	4.00×10^-3^	45.50%	0.1	0.9	ABC	0.15	Weak
	Asian	4	2859/2537	0.70 (0.53, 0.92)	0.01	51.10%	0.11				
	Caucasian	2	236/483	0.66 (0.29, 1.48)	0.31	65.40%	0.09				

FPRP, false positive report probability.^§^Cumulative epidemiological evidence as graded by Venice criteria and FPRP. ^†^Only Asian or Caucasian data were available for meta-analysis.

OR, odds ratio; CI, confidence interval; FPRP, false positive report probability; HLA, human leukocyte antigen.

As to variants located outside the HLA region (non-HLA genes), 14 variants within 11 genes were found to be significantly associated with PBC risk (**Table 3**). Five variants (rs231775, rs231725, rs9303277, rs1864325 and rs1544410) reached genome-wide significance (*P* < 5.0×10-8) across all ancestries, among which rs9303277 and rs1864325 were (or in LD with) previously identified by GWAS. Of these, rs1544410 within *VDR* exhibited the strongest association with PBC risk (OR=1.62, 95% CI=1.37-1.93, *P*=2.99×10^-08^). Subgroup analyses suggested only rs231775 in *CTLA-4* (OR=1.31, 95% CI=1.21-1.41, *P*=3.28×10^-12^) and rs9303277 in *IKZF3* were identified as genome-wide significant loci in Asian population. No significant associations were observed for another 7 variants within five non-HLA genes (**Supplementary Table S8**).

**Table 3:** Variants in Non-*HLA* genes significantly associated with risk of primary biliary cholangitis in meta-analysis.

Gene	Variant	Allele^*^	Ethnicity	Data sets	Cases/ Controls	Risk estimates		Heterogeneity		P for Inter action	Venice criteria grade	FPRP	Cumulative evidence of association§
						OR (95% CI)	*P*	*І* ^2^	*P*				
*CTLA-4*	rs231725	A/G	All^‡^	5	1421/1293	1.32 (1.20, 1.45)	6.67×10^-9^	0.00%	0.97	0.7	BAA	<0.001	Strong
			Asian	4	1070/1014	1.31 (1.18, 1.45)	2.28×10^-7^	0.00%	0.93				
*CTLA-4*	rs5742909	T/C	All^‡^	8	1967/2818	0.76 (0.66, 0.87)	4.24×10^-5^	0.00%	0.83	0.14	BAA	0.011	Strong
			Asian	4	697/803	0.87 (0.70, 1.08)	0.21	0.00%	0.81				
			Caucasian	4	1270/2015	0.70 (0.60, 0.83)	3.07×10^-5^	0.00%	0.95				
*CTLA-4*	rs231775	G/A	All^‡^	12	2844/3738	1.31 (1.21, 1.41)	3.28×10^-12^	11.40%	0.33	0.16	AAA	<0.001	Strong
			Asian	5	1147/1174	1.39 (1.26, 1.54)	4.00×10^-10^	0.00%	0.49				
			Caucasian	7	1697/2564	1.25 (1.13, 1.39)	1.99×10^-5^	12.70%	0.33				
*CTLA-4*	rs3087243	A/G	All^‡^	8	2249/2991	0.80 (0.72, 0.89)	2.30×10^-5^	23.40%	0.24	0.51	AAA	<0.001	Strong
			Asian	4	1014/1071	0.78 (0.68, 0.89)	1.96×10^-4^	0.00%	0.51				
			Caucasian	4	1179/1977	0.84 (0.70, 1.00)	0.05	54.70%	0.09				
*STAT4*	rs7574865	T/G	Asian†	3	1685/1927	1.30 (1.18, 1.45)	5.53×10^-7^	7.20%	0.34		AAA	<0.001	Strong
*IKZF3*	rs9303277	T/C	All^‡^	3	1816/2430	1.36 (1.24, 1.49)	8.58×10^-11^	0.00%	0.61	0.36	AAA	<0.001	Strong
			Asian	2	1373/1496	1.39 (1.25, 1.56)	3.10×10^-9^	0.00%	0.71				
*AKAP11*	rs9533090	T/C	All^‡^	3	2976/7251	1.20 (1.12, 1.29)	1.23×10^-7^	0.00%	1	0.97	AAA	<0.001	Strong
			Caucasian	2	2293/6099	1.20 (1.12, 1.30)	1.20×10^-6^	0.00%	1				
*VDR*	rs1544410	T/C	All^‡^	5	609/1015	1.62 (1.37, 1.93)	2.99×10^-8^	0.00%	0.44	0.37	BAA	<0.001	Strong
			Asian	2	253/339	2.35 (0.99, 5.56)	0.05	52.60%	0.15				
			Caucasian	3	356/676	1.56 (1.29, 1.89)	3.82×10^-6^	0.00%	0.7				
*CLDN14*	rs170183	G/A	All^‡^	3	2976/7251	0.87 (0.81, 0.93)	4.82×10^-5^	0.00%	0.5	0.44	AAC	0.001	Moderate
*MAPT*	rs1864325	T/C	All^‡^	3	2976/7251	0.78 (0.72, 0.85)	4.71×10^-9^	0.00%	0.75	0.63	AAC	<0.001	Moderate
*IL12RB2*	rs3790567	A/G	All^‡^	4	1447/1948	1.27 (1.02, 1.58)	0.04	72.40%	0.01	0.02	ACA	0.68	Weak
			Asian	2	698/756	1.06 (0.87, 1.30)	0.06	29.30%	0.23				
			Caucasian	2	749/1192	1.49 (1.21, 1.84)	1.48×10^-4^	42.20%	0.19				
*FGFR1OP* */CCR6*	rs9459874	C/T	All^‡^	3	10959/21706	1.17 (1.05, 1.32)	5.00×10^-3^	81.70%	0	0.54	ACA	0.14	Weak
			Caucasian	2	8464/17423	1.16 (0.97, 1.37)	0.1	74.50%	0.05				
*IL-10*	-1082 G/A	G/A	All^‡^	3	236/303	1.55 (1.08, 2.22)	0.02	0.00%	0.6	0.7	BAC	0.44	Weak
			Asian	2	142/231	1.41 (0.77, 2.56)	0.27	0.00%	0.35				
*TNF-α*	rs1800629	A/G	All^‡^	6	681/864	0.78 (0.63, 0.96)	0.02	0.00%	0.76	0.5	BAC	0.58	Weak
			Caucasian	5	624/781	0.79 (0.64, 0.99)	0.04	0.00%	0.71				

FPRP, false positive report probability. ^*^ Risk allele versus reference allele. ^§^Cumulative epidemiological evidence as graded by Venice criteria and FPRP. ^†^Only Asian or Caucasian data were available for meta-analysis. ^‡^ All ancestries were included.

OR, odds ratio; CI, confidence interval; FPRP, false positive report probability; HLA, human leukocyte antigen.

### 3.4 Heterogeneity, sensitivity analysis, and bias in the meta-analysis

Of the 70 meta-analysis, 23 (32.9%) had high heterogeneity, 6 (8.6%) had moderate heterogeneity, and 41 (58.6%) had no or little heterogeneity. The proportion of high heterogeneity in the 43 significant associations was lower than that in the remaining 26 non-significant associations (22.7% vs 42.3%%). Subgroup analyses (**Supplementary Table S9**) showed that diagnostic criteria, genotyping methodology, and ethnicity might be the source of heterogeneity (*P* for interaction <0.05). Meta-regression indicated that ethnicity and diagnostic criteria might contribute to the heterogeneity of DQB10402 (*P*=0.011) and DQA10102 (*P*=0.007), respectively. Sensitivity analyses by excluding the initial published or positive study were performed for the 44 variants significantly related to PBC-risk. The results indicated that 75.0% of the significant association was robustness, and the other 25.0% was no longer significant when excluding the initial positive study (**Supplementary Table S10**). Publication bias was evaluated by Begg’s tests. Six variants (rs170183, DRB1*0802, DQA1*0401, DQB1*0402, DPB1*0501) indicated evidence of publication bias (*P* < 0.10). As to bias due to small studies (estimated by Egger tests), four variants (rs1864325, DRB1*0405, DRB1*14, DRB1*0802) showed evidence of possible small study bias (*P* < 0.10) (**Supplementary Table S10**).

### 3.5 Cumulative evidence assessment

In the evaluation of the cumulative evidence for the 44 significant associations (**Table 2** and **Table 3**), grades of A were given to 34, 29, and 28 variants for the amount of evidence, replication of the association, and protection from bias, respectively by the Venice criteria. Grades of B were given to 10, five, and zero associations for each of the three criteria. Grades of C were given to 16 variants for protection from bias (**Supplementary Table S10**), mainly due to the loss of significance after excluding the initial report (n=10), small study bias (n=4) and significant publication bias (n=4). Significant associations with PBC-risk had a calculated FPRP < 0.05 for 29 variants, FPRP 0.05-0.20 for 6 variant, and FPRP > 0.20 for 9 variants. By integrating the Venice criteria and FPRP, cumulative epidemiological evidence of a significant relationship was graded as strong for 17 variants (9 within 5 *HLA* genes and 8 within 5 non-HLA genes), moderate for 14 variants (13 within 4 *HLA* genes and 2 within 2 non-HLA genes), and weak for 13 variants (8 within 3 *HLA* genes and 4 within 4 non-HLA genes).

### 3.6 Functional annotation and pathway analysis

Functional annotation was further conducted using HaploReg V4.1 for the variants that significant associated with PBC risk (**Supplementary Table S11**). The results suggested that these variants and their highly correlated SNPs might fall within a DNase I hypersensitivity site, a strong prompter, and an enhancer activity region. Of these variants, rs231775 was missense located in the *CTLA4* gene. GTEx tissue enrichment analysis indicated that the significant mapped genes for PBC were significantly enriched in the small intestine, lymphocytes, lungs, spleen, brain, and blood (**Supplementary Figure S1**). In addition, GO pathway analysis across these significantly mapped genes revealed enrichment in 10 biological pathways (FDR < 0.05), primarily involved in immune cell regulation and immune response-regulating signaling pathways (**Table 4**).

**Table 4:** GO pathway analysis across the significant mapped genes of primary biliary cholangitis.

Gene Set	Description	Enrichment Ratio	*P*	FDR
GO:1903131	Mononuclear cell differentiation	8.9127	6.36×10^-13^	5.33×10^-10^
GO:0070661	Leukocyte proliferation	10.261	1.00×10^-11^	4.21×10^-9^
GO:0001819	Positive regulation of cytokine production	7.873	9.73×10^-11^	2.72×10^-8^
GO:0051249	Regulation of lymphocyte activation	7.6349	1.53×10^-10^	3.21×10^-8^
GO:0042113	B cell activation	10.404	1.30×10^-9^	2.18×10^-7^
GO:0019221	Cytokine-mediated signaling pathway	7.1005	1.72×10^-9^	2.40×10^-7^
GO:0097696	Receptor signaling pathway via STAT	13.525	2.86×10^-9^	3.43×10^-7^
GO:0046631	Alpha-beta T cell activation	13.222	3.56×10^-9^	3.60×10^-7^
GO:0050867	Positive regulation of cell activation	8.2935	3.86×10^-9^	3.60×10^-7^
GO:0002250	Adaptive immune response	7.1413	6.08×10^-9^	5.10×10^-7^

FDR, false discovery rate.

### 3.7 Phenome-wide analysis

**Figure 3:**
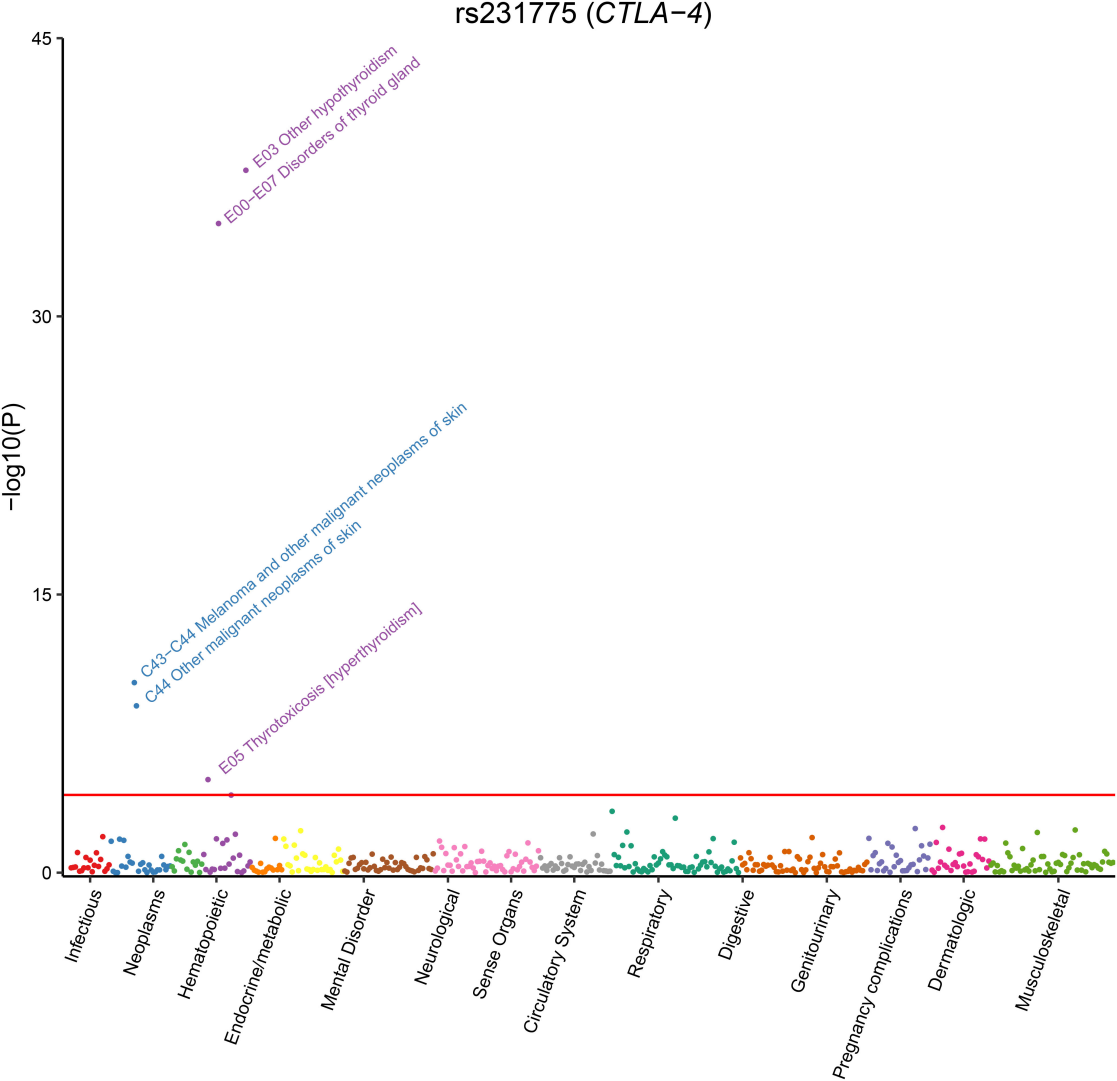
Phenome-wide analysis of rs231775 using data from UK Biobank.

Finally, we performed phenome-wide analysis for the two additional genome-wide significant SNPs, rs1544410 and rs231725 (rs231775 and rs231725 were in strong LD with R^2^ = 0.85), identified by our meta-analysis. The results suggested that rs231725 was primarily associated with thyroid problems (such as hypothyroidism, thyroid gland disorders, and hyperthyroidism/thyrotoxicosis) and melanoma (**Figure 3** **and** **Supplementary Table S12**). However, no significant association was identified for rs1544410.

## 4 Discussion

To the best of our knowledge, this is the largest and most comprehensive study to systematically assess the relationship between genetic variants (in HLA region and non-HLA region) and risk of PBC. This research incorporated data from 105 articles that involved 71,031 cases and 140,499 controls. Meta-analyses of candidate-gene association studies identified 44 variants significantly associated with PBC risk (30 variants within six *HLA* genes and 14 variants within 11 non-HLA genes). Separately, published GWAS reported 115 significant variants. Among these variants, nine variants (eight variants in *HLA* genes and rs7574865 in *STAT4*) were identified by both approaches. Cumulative epidemiological evidence graded 17 strong, 14 moderate, and 13 weak associations. Notably, strong evidence supports the missense variant rs231775 in *CTLA4* as a genome-wide significant locus, emphasizing its potential role in PBC pathogenesis. In addition, tissue enrichment analysis and phenome-wide analysis showed that PBC may share a common genetic architecture with some autoimmune diseases. This study comprehensively evaluated published research on the relationship between genetic variants and risk of PBC. These findings improve our current understanding of the genetic architecture of this disease.

The HLA has been extensively studied in a variety of immune-mediated diseases, such as rheumatoid arthritis (42), inflammatory bowel disease (43), and autoimmune hepatitis (7, 44). Our study confirmed the importance of variations in the *HLA* gene in the pathogenesis of PBC. Specifically, eight variants (DQA1*0401, DQB1*0301, DQB1*0402, DQB1*0602, DRB1*08, DRB1*0803, DRB1*11, and DRB1*1101) have been shown to be associated with PBC both in published GWAS and meta-analyses of candidate-gene association studies. Among these variants, strong evidence supports four variants (DRB1*08, DRB1*1101, DRB1*0803, and DQB1*0301) were associated with PBC at the genome-wide significance level by our meta-analyses. These results are consistent with previous studies, indicating that *HLA* is a susceptibility gene for PBC (7, 33).

The HLA-DRB1*08 allele family has been the most extensively studied in terms of PBC susceptibility. Our meta-analysis suggests that DRB1*0803 is associated with PBC at the genome-wide significance level with strong evidence, which is also verified by a published GWAS of in the Chinese population (7). Another variant in this family that exhibits the strongest association with PBC is DRB1*0801 (OR=3.11). This variant is significantly associated with PBC in Caucasian populations, yet its association in Japanese populations has been reported as non-significant (45). However, cumulative evidence grades this association as moderate, and it has not been replicated in large-scale studies such as GWAS. Studies have indicated that DRB1*0801 plays a crucial role in disrupting hepatic self-tolerance by binding and presenting charged pyruvate dehydrogenase E2 (PDC-E2) peptides (46). Additionally, this allele family is a major susceptibility factor for autoimmune hepatitis in white European and American populations (47–49), and is also associated with a reduced risk of primary sclerosing cholangitis (50).

In addition to the *HLA* locus, non-HLA genes also play an important role in the pathogenesis of PBC. Strong evidence from meta-analysis indicates that rs7574865 in *STAT4* is a risk variant for PBC in Asian populations. Furthermore, published GWAS have also identified its association with PBC susceptibility in the British population (32). Rs7574865 located in the third intron of the *STAT4* gene. Although this variant does not disrupt any transcription factor binding sites (51), it has been suggested to affect alternative splicing and is associated with *STAT4* gene upregulation (52). This allele is also linked to an increased risk of rheumatoid arthritis (53) and ulcerative colitis (54). Furthermore, strong evidence from meta-analysis supports seven additional associations, four of which reached genome-wide significance (rs231725, rs231775, rs1544410, and rs9303277). Among these, rs231775 is a missense variant located in exon 1 of *CTLA4*, resulting in a threonine-to-alanine amino acid change (p.Thr17Ala). Functional evidence suggests that the A (Thr) allele increases CTLA-4 surface expression, which may modulate T-cell regulation and thus contribute to pathogenesis of autoimmune diseases such as PBC, although the possibility remains that it is a tag SNP in linkage disequilibrium with an untyped causal variant (55, 56). Another significant variant, rs231725, resides in the 3′-UTR of *CTLA4* and has been reported to regulate mRNA stability and translational efficiency (57). This SNP reduces CTLA-4 expression and modulates CD4^+^ T-cell signaling thresholds, potentially contributing to PBC pathogenesis (58). These results are consistent with previous studies showing that CTLA-4 inhibitors (such as ipilimumab) enhance T-cell activation, potentially increasing the risk of autoimmune disorders (59, 60). In contrast, abatacept—a CTLA-4 agonist that inhibits T-cell activation—is currently under evaluation in a multicenter trial for UDCA-unresponsive PBC patients (NCT02078882) (61). In addition, phenome-wide analyses have suggested that PBC may share a common genetic architecture with certain autoimmune diseases, such as hypothyroidism/myxedema, hyperthyroidism/thyrotoxicosis, and inflammatory bowel disease. These results were consistent with clinical observations that PBC could coexist with other autoimmune diseases (62, 63), or hematological disorders (64). These findings could help develop strategies for the prevention and treatment of PBC and other related diseases.

However, our study had several limitations. First, our meta-analyses were conducted for variants with at least three independent datasets, which may have resulted in other important PBC-associated variants being overlooked (354 variants with only one dataset). However, we further performed meta-analysis for variants with two datasets and identified additional 11 loci significantly associated with PBC risk (**Supplementary Table S13**). Second, although functional variants have been identified, it is unknown whether they are causal variants, and further research is required to address this issue. Third, despite sensitivity analyses suggested robustness for most of the associations, a large heterogeneity was found in approximately 30% of the associations. To explore the potential sources of heterogeneity, we conducted subgroup analyses and meta-regression. The results suggested that ethnicity, diagnostic criteria for PBC, and genotyping methods may all contribute to the heterogeneity. Among these, ethnicity appeared to be a major factor, which may be partially explained by differences in allele frequencies across populations. For example, the HLA allele DQB1*0601 had an allele frequency of 0.109 in East Asians but only 0.013 in Europeans (**Supplementary Table S14**), consistent with its significant association in Asian populations only. Such differences underscore the importance of considering population background and methodological variations in genetic meta-analyses. Finally, although the HLA region demonstrates strong association with PBC risk, the low population incidence of PBC results in poor positive/negative predictive values for clinical screening. Nonetheless, this study identified disease-associated variants within this region and provides mechanistic insights for future investigation.

This comprehensive landmark study delivers the most extensive genetic dissection of PBC to date. Meta-analyses of candidate-gene association studies identified 44 risk-associated variants, comprising 30 variants within six *HLA* genes and 14 variants within 11 non-HLA genes. Among these variants, 17 across 10 genes supported by strong epidemiological evidence. Published GWAS have separately reported 115 significant variants associated with PBC. Notably, nine variants were identified by both approaches: the *HLA* alleles DQA1*0401, DQB1*0301, DQB1*0402, DQB1*0602, DRB1*08, DRB1*0803, DRB1*11, and DRB1*1101, along with the *STAT4* variant rs7574865. Our findings not only consolidate the current understanding of PBC susceptibility but also uncover previously unappreciated genetic features underlying disease pathogenesis.

## Data Availability

The original contributions presented in the study are included in the article/**Supplementary Material**. Further inquiries can be directed to the corresponding authors.
