# Supplementary material for: Genetic architecture of primary biliary cholangitis: strong evidence for HLA and non-HLA risk loci
Source: Front Immunol. 2025 Aug 29;16:1600364. doi: 10.3389/fimmu.2025.1600364 (PMC12425925; doi:10.3389/fimmu.2025.1600364)
Supplement: Supplementary file 1 [file DataSheet1.pdf]

Supplementary Material

List of supplementary material:

1.1 Supplementary Figures:

Supplementary Fig. S1. GTEx tissue enrichment analysis for expression of the mapped genes significant with primary biliary cholangitis.

1.2 Supplementary tables:

Supplementary Table 1. Checklist of Preferred Reporting Items for Systematic Reviews and Meta-Analyses.

Supplementary Table 2. Characteristics of the genome-wide association studies of primary biliary cirrhosis.

Supplementary Table 3. Characteristics of the the candidate-gene association studies on variants in Non-HLA genes and risk of primary biliary cirrhosis in the meta-analysis

Supplementary Table 4. Characteristics of the candidate-gene association studies on variants in Non-HLA genes and risk of primary biliary cirrhosis with datasets less than three.

Supplementary Table 5. Characteristics of the the candidate-gene association studies on variants in HLA genes and risk of primary biliary cirrhosis in the meta-analysis

Supplementary Table 6. Characteristics of the candidate-gene association studies on variants in HLA genes and risk of primary biliary cirrhosis with datasets less than three

Supplementary Table 7. Variants in HLA gene that were not associated with risk of PBC in meta-analysis.

Supplementary Table 8. Variants in Non-HLA gene that were not associated with PBC in meta-analysis.

Supplementary Table 9. Subgroup analyses stratified by diagnostic items and genotyping methods for the associations with high heterogeneity.

Supplementary Table 10. Details of protection from bias for variants significantly associated with risk of diseases.

Supplementary Table 11. Functional annotation for variants associated with PBC through HeploReg V4.1.

Supplementary Table 12. Significant association in phenome-wide analysis of the novel variant using data from UK Biobank.

Supplementary Table 13. Variants significant associated with risk of primary biliary cirrhosis in meta-analysis with two datasets.

Supplementary Table 14. Allele frequency of variants in HLA region of Asia and Europe.

1 Supplementary Figures and Tables

1.1 Supplementary Figures

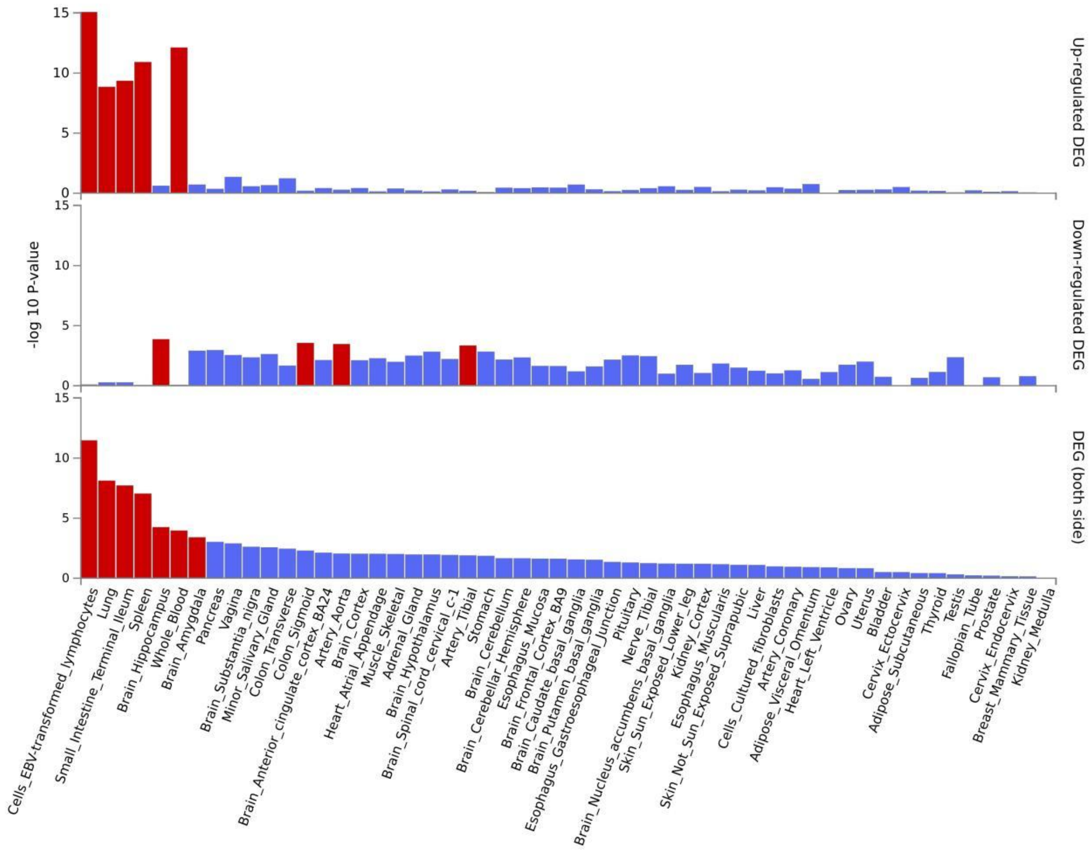

Supplementary Fig. S1. GTEx tissue enrichment analysis for expression of the mapped genes significant with primary biliary cholangitis. Red represents significant tissue enrichment after Benjamin-Hochberg correction.

1.2 Supplementary tables

Supplementary table 1 Checklist of Preferred Reporting Items for Systematic Reviews and Meta-Analyses.

Section and Topic	Item #	Checklist item	Location where item is reported
**TITLE**			
Title	1	Identify the report as a systematic review.	P1
**ABSTRACT**			
Abstract	2	See the PRISMA 2020 for Abstracts checklist.	P2
**INTRODUCTION**			
Rationale	3	Describe the rationale for the review in the context of existing knowledge.	P3
Objectives	4	Provide an explicit statement of the objective(s) or question(s) the review addresses.	P3
**METHODS**			
Eligibility criteria	5	Specify the inclusion and exclusion criteria for the review and how studies were grouped for the syntheses.	P4
Information sources	6	Specify all databases, registers, websites, organisations, reference lists and other sources searched or consulted to identify studies. Specify the date when each source was last searched or consulted.	P4
Search strategy	7	Present the full search strategies for all databases, registers and websites, including any filters and limits used.	P4
Selection process	8	Specify the methods used to decide whether a study met the inclusion criteria of the review, including how many reviewers screened each record and each report retrieved, whether they worked independently, and if applicable, details of automation tools used in the process.	P4
Data collection process	9	Specify the methods used to collect data from reports, including how many reviewers collected data from each report, whether they worked independently, any processes for obtaining or confirming data from study investigators, and if applicable, details of automation tools used in the process.	P4
Data items	10a	List and define all outcomes for which data were sought. Specify whether all results that were compatible with each outcome domain in each study were sought (e.g. for all measures, time points, analyses), and if not, the methods used to decide which results to collect.	P4
	10b	List and define all other variables for which data were sought (e.g. participant and intervention characteristics, funding sources). Describe any assumptions made about any missing or unclear information.	P4
Study risk of bias assessment	11	Specify the methods used to assess risk of bias in the included studies, including details of the tool(s) used, how many reviewers assessed each study and whether they worked independently, and if applicable, details of automation tools used in the process.	P5

Section and Topic	Item #	Checklist item	Location where item is reported
Effect measures	12	Specify for each outcome the effect measure(s) (e.g. risk ratio, mean difference) used in the synthesis or presentation of results.	P5
Synthesis methods	13a	Describe the processes used to decide which studies were eligible for each synthesis (e.g. tabulating the study intervention characteristics and comparing against the planned groups for each synthesis (item #5)).	P5
	13b	Describe any methods required to prepare the data for presentation or synthesis, such as handling of missing summary statistics, or data conversions.	P5
	13c	Describe any methods used to tabulate or visually display results of individual studies and syntheses.	P5
	13d	Describe any methods used to synthesize results and provide a rationale for the choice(s). If meta-analysis was performed, describe the model(s), method(s) to identify the presence and extent of statistical heterogeneity, and software package(s) used.	P5
	13e	Describe any methods used to explore possible causes of heterogeneity among study results (e.g. subgroup analysis, meta-regression).	P5
	13f	Describe any sensitivity analyses conducted to assess robustness of the synthesized results.	P5
Reporting bias assessment	14	Describe any methods used to assess risk of bias due to missing results in a synthesis (arising from reporting biases).	P5
Certainty assessment	15	Describe any methods used to assess certainty (or confidence) in the body of evidence for an outcome.	P5
**RESULTS**			
Study selection	16a	Describe the results of the search and selection process, from the number of records identified in the search to the number of studies included in the review, ideally using a flow diagram.	P6, Fig. 1
	16b	Cite studies that might appear to meet the inclusion criteria, but which were excluded, and explain why they were excluded.	P6, Tables S2-6
Study characteristics	17	Cite each included study and present its characteristics.	P6, Tables S2-6
Risk of bias in studies	18	Present assessments of risk of bias for each included study.	P7
Results of individual studies	19	For all outcomes, present, for each study: (a) summary statistics for each group (where appropriate) and (b) an effect estimate and its precision (e.g. confidence/credible interval), ideally using structured tables or plots.	Tables S2-6
Results of syntheses	20a	For each synthesis, briefly summarise the characteristics and risk of bias among contributing studies.	P6-P7
	20b	Present results of all statistical syntheses conducted. If meta-analysis was done, present for each the summary estimate and its precision (e.g. confidence/credible interval) and measures of statistical heterogeneity. If	P6-P7, Table 1, Table 2, Table S7, Table S8

Section and Topic	Item #	Checklist item	Location where item is reported
		comparing groups, describe the direction of the effect.	
	20c	Present results of all investigations of possible causes of heterogeneity among study results.	Table S9
	20d	Present results of all sensitivity analyses conducted to assess the robustness of the synthesized results.	Table S9
Reporting biases	21	Present assessments of risk of bias due to missing results (arising from reporting biases) for each synthesis assessed.	Table S9
Certainty of evidence	22	Present assessments of certainty (or confidence) in the body of evidence for each outcome assessed.	Tables 1-3, Table S8
**DISCUSSION**			
Discussion	23a	Provide a general interpretation of the results in the context of other evidence.	P8
	23b	Discuss any limitations of the evidence included in the review.	P9
	23c	Discuss any limitations of the review processes used.	P9
	23d	Discuss implications of the results for practice, policy, and future research.	P9
**OTHER INFORMATION**			
Registration and protocol	24a	Provide registration information for the review, including register name and registration number, or state that the review was not registered.	Cumulative Evidence on Associations between Genetic Variants and Autoimmune liver diseases (CRD42021282146)
	24b	Indicate where the review protocol can be accessed, or state that a protocol was not prepared.	PROSPERO
	24c	Describe and explain any amendments to information provided at registration or in the protocol.	None
Support	25	Describe sources of financial or non-financial support for the review, and the role of the funders or sponsors in the review.	This work was supported by Senior Medical Talents Program of Chongqing for Young and Middle-aged (No. YXGD202440). The funding agency had no role in study design, data collection, data management, data analysis, data interpretation, writing of the manuscript, or submission decision.
Competing	26	Declare any competing interests of review authors.	The authors declare

Section and Topic	Item #	Checklist item	Location where item is reported
interests			no conflicts of interest
Availability of data, code and other materials	27	Report which of the following are publicly available and where they can be found: template data collection forms; data extracted from included studies; data used for all analyses; analytic code; any other materials used in the review.	Data used for all analyses was extracted from included studies

From: Page MJ, McKenzie JE, Bossuyt PM, Boutron I, Hoffmann TC, Mulrow CD, et al. The PRISMA 2020 statement: an updated guideline for reporting systematic reviews. BMJ 2021;372:n71. doi: 10.1136/bmj.n71

Supplementary Table 2. Characteristics of the genome-wide association studies of primary biliary cirrhosis.

Study, PMID	Country/ Region	Locus	*Gene*	Variant	Cases/Controls	A1/A2	MAF	*OR (95%CI)*	P
**Non-HLA region studies**									
Qiu 2017, 28425483	China	1p13.1	*CD58*	rs2300747	2029/6163	A/G	0.48/0.49	1.29 (1.20, 1.39)	1.11E-11
Qiu 2017, 28425483	China	1p13.1	*CD58*	rs10924106	2029/6163	A/G	0.51/0.45	1.28 (1.19, 1.37)	6.42E-12
Cordell 2021, 34033851	Mix_Europe	1p13.1	*CD58*	rs10802191	8021/16489	A/T	NA/NA	0.81 (0.76, 0.87)	1.89E-08
Cordell 2021, 34033851	Mix_Europe	1p31	*IL12RB2*	rs6679356	8021/16489	C/T	NA/NA	1.55 (1.47, 1.63)	5.84E-64
Cordell 2015, 26394269	Mix_Europe	1p31	*IL12RB2*	rs6679356	2764/10475	C/T	NA/0.21	1.52 (1.41, 1.64)	6.60E-28
Liu 2012, 22961000	UK	1p31	*Il12RB2*	rs72678531	2861/8514	G/A	NA/0.17	1.61 (1.49, 1.73)	7.45E-36
Juran 2012, 22936693	Mix_Europe	1p31	*IL12RB2*	rs72678531	2216/5594	C/T	0.26/0.17	1.68 (1.51, 1.86)	1.75E-22
Mells 2011, 21399635	UK	1p31	*IL12RB2*	rs17129789	1840/5163	C/T	0.18/0.25	1.52 (1.39, 1.67)	3.76E-19
Liu 2010, 20639880	Italy	1p31	*IL12RB2*	rs3790567	934/4651	A/G	0.38/0.31	1.48 (1.32, 1.66)	7.63E-12
Hirschfield 2010, 20639879	Mix_Europe	1p31	*IL12RB2*	rs3790567	1351/4700	C/A	0.33/0.24	1.38 (1.25, 1.52)	1.08E-10
Hirschfield_2009, 19458352	Canada, US	1p31	*IL12RB2*	rs3790567	1031/2713	A/C	0.34/0.24	1.51 (1.33, 1.70)	4.65E-11
Hirschfield_2009, 19458352	Canada, US	1p31	*IL12RB2*	rs3790565	1031/2713	C/AGT	0.26/0.18	1.46 (1.28, 1.67)	2.44E-08
Cordell 2021, 34033851	Mix_Europe	1q31.3	*DENND1B*	rs12123169	8021/16489	A/T	NA/NA	1.24 (1.18, 1.31)	2.78E-18
Liu 2012, 22961000	UK	1q31.3	*DENND1B*	rs2488393	2861/8514	A/G	NA/0.21	1.28 (1.19, 1.37)	6.42E-12
Mells 2011, 21399635	UK	1q31.3	*DENND1B*	rs12134279	2460/7677	T/C	0.2/0.25	1.34 (1.25, 1.45)	1.08E-14
Cordell 2015, 26394269	Mix_Europe	1q31.3	*DENND1B*	rs17641524	2764/10475	C/T	NA/0.83	0.77 (0.71, 0.83)	1.01E-11
Cordell 2021, 34033851	Mix_Europe	1p36.32	*TNFSF14*	rs867436	8021/16489	T/C	NA/NA	1.14 (1.09, 1.20)	5.67E-09
Mells 2011, 21399635	UK	1p36	*MMEL1*	rs10752747	1840/5163	T/G	0.34/0.37	1.13 (1.04, 1.22)	0.002
Hirschfield 2010, 20639879	Mix_Europe	1p36	*MMEL1*	rs3748816	1351/4700	C/T	0.41/0.34	1.33 (1.20, 1.47)	3.62E-08
Cordell 2021, 34033851	Mix_Europe	1q23.1	*FCRL3*	rs945635	8021/16489	G/C	NA/NA	0.89 (0.85, 0.92)	2.93E-08
Cordell 2021, 34033851	Mix_Europe	1q32.1	*INAVA*	rs55734382	8021/16489	T/C	NA/NA	0.87 (0.83, 0.91)	1.15E-09
Cordell 2021, 34033851	Mix_Europe	2p23.3	*DNMT3A*	rs34655300	8021/16489	T/C	NA/NA	1.15 (1.10, 1.20)	4.75E-10
Cordell 2015, 26394269	Mix_Europe	2p23.1	*LBH*	rs4952108	2764/10475	C/T	NA/0.81	1.28 (1.17, 1.40)	5.05E-08
Cordell 2021, 34033851	Mix_Europe	2p25.1	*ID2*	rs891058	8021/16489	A/G	NA/NA	0.89 (0.85, 0.93)	5.73E-07
Cordell 2015, 26394269	Mix_Europe	2q12.1	*L1R1/IL1RL2*	rs12712133	6480/14736	A/G	NA/0.56	1.14 (1.07, 1.21)	5.01E-08
Cordell 2021, 34033851	Mix_Europe	2q21.3	*TMEM163*	rs859767	8021/16489	G/A	NA/NA	0.87 (0.83, 0.91)	1.51E-09

Cordell 2021, 34033851	Mix_Europe	2q32.2	*NAB1*	rs3771317	8021/16489	C/T	NA/NA	1.34 (1.26, 1.42)	4.18E-22
Cordell 2015, 26394269	Mix_Europe	2q32.3	*NAB1*	rs3771317	2764/10475	T/C	NA/0.86	0.71 (0.65, 0.78)	2.29E-14
Qiu 2017, 28425483	China	2q32	*STAT4*	rs10168266	2029/6163	A/G	0.4/0.34	1.31 (1.22, 1.41)	2.61E-13
Hitomi 2024, 38652555	Japan	2q32	*STAT4*	rs11889341	2181/2699	T/C	0.36/0.31	1.33 (1.21, 1.45)	3.32E-10
Mells 2011, 21399635	UK	2q32	*STAT4*	rs10931468	2460/7677	A/C	0.12/0.16	1.50 (1.37, 1.64)	9.94E-19
Liu 2012, 22961000	UK	2q32	*STAT4*	rs3024921	2861/8514	A/T	NA/0.06	1.62 (1.45, 1.80)	2.21E-18
Juran 2012, 22936693	Mix_Europe	2q32	*STAT4*	rs3024921	2216/5594	A/T	0.1/0.06	1.75 (1.48, 2.07)	6.22E-11
Liu 2012, 22961000	UK	2q32	*STAT4*	rs7574865	2861/8514	A/T	NA/0.22	1.31 (1.22, 1.40)	1.45E-14
Hirschfield_2009, 19458352	Canada, US	2q32	*STAT4*	rs16833239	1031/2713	G/A	0.96/0.96	1.65 (1.30, 2.10)	4.25E-05
Qiu 2017, 28425483	China	2q33.2	*CD28/CTLA4*	rs4675369	2029/6163	G/A	0.53/0.45	1.31 (1.22, 1.41)	2.61E-13
Qiu 2017, 28425483	China	2q33.2	*CD28/CTLA4*	rs7599230	2029/6163	G/A	0.47/0.41	1.26 (1.18, 1.36)	1.76E-10
Cordell 2021, 34033851	Mix_Europe	2q33.2	*CD28*	rs10581773	8021/16489	AT/A	NA/NA	1.24 (1.14, 1.35)	4.50E-07
Hirschfield_2009, 19458352	Canada, US	2q33	*CTLA-4*	rs6748358	505/1507	C/AT	0.57/0.5	0.72 (0.62, 0.84)	2.77E-05
Cordell 2015, 26394269	Mix_Europe	2q36.3	*IL18RAP*	rs4973341	4688/12221	C/T	NA/0.67	0.82 (0.74, 0.90)	2.34E-10
Cordell 2021, 34033851	Mix_Europe	3p24.2	*RARB*	rs6550965	8021/16489	A/C	NA/NA	1.18 (1.13, 1.23)	1.27E-13
Cordell 2021, 34033851	Mix_Europe	3p24.3	*PLCL2*	rs9876137	8021/16489	G/A	NA/NA	1.15 (1.11, 1.21)	5.93E-11
Mells 2011, 21399635	UK	3p24.3	*PLCL2*	rs1372072	3394/12328	A/G	0.37/0.4	1.20 (1.12, 1.27)	1.30E-08
Cordell 2015, 26394269	Mix_Europe	3p24.3	*PLCL2*	rs1372072	2764/10475	A/G	NA/0.38	1.20 (1.12, 1.28)	4.60E-08
Hitomi 2024, 38652555	Japan	3q13.33	*CD80*	rs9855065	2181/2699	A/G	0.24/0.3	0.72 (0.66, 0.79)	1.51E-12
Hitomi 2019, 30643196	Japan	3q13.33	*ARHGAP31*	rs9855065	1855/1719	A/G	NA/NA	0.73 (0.65, 0.81)	3.41E-09
Hitomi 2019, 30643196	Japan	3q13.33	*CD80*	rs57271503	1855/1719	A/G	NA/0.34	0.72 (0.65, 0.81)	3.97E-09
Hitomi 2019, 30643196	Japan	3q13.33	*TIMMDC1/TMEM39A*	rs2293370	1855/1719	A/G	NA/NA	1.37 (1.23, 1.53)	6.23E-09
Kawashima 2017, 28062665	Japan	3q13.33	*C3orf1*	rs2293370	1381/1505	A/G	0.23/0.29	1.34 (1.19, 1.51)	1.38E-06
Nakamura 2012, 23000144	Japan	3q13.33	*CD80*	rs2293370	1274/1091	A/G	NA/NA	1.48 (1.30, 1.69)	3.04E-09
Qiu 2017, 28425483	China	3q13.33	*CD80*	rs3732421	2029/6163	G/A	0.27/0.33	0.74 (0.68, 0.80)	3.79E-13
Cordell 2021, 34033851	Mix_Europe	3q13.33	*CD80/TIMMDC1*	rs2293370	8021/16489	A/G	NA/NA	0.74 (0.70, 0.78)	6.33E-25
Liu 2012, 22961000	UK	3q13.33	*CD80*	rs2293370	2861/8514	G/ACT	NA/0.8	1.39 (1.29, 1.52)	3.61E-15
Mells 2011, 21399635	UK	3q13.33	*CD80*	rs2293370	2460/7677	G/ACT	0.8/0.85	1.41 (1.27, 1.56)	5.80E-11
Cordell 2015, 26394269	Mix_Europe	3q13.33	*TIMMDC1*	rs2293370	2764/10475	G/A	NA/0.86	1.42 (1.30, 1.55)	4.19E-15
Juran 2012, 22936693	Mix_Europe	3q13.33	*TIMMDC1*	rs1131265	2216/5594	G/CT	0.85/0.8	1.42 (1.26, 1.60)	8.72E-09
Qiu 2017, 28425483	China	3q25	*IL12A*	rs582537	2029/6163	C/A	0.23/0.29	0.75 (0.69, 0.82)	6.44E-11

Liu 2012, 22961000	UK	3q25	*IL12A*	rs80014155	2861/8514	A/G	NA/0.004	3.44 (2.39, 4.94)	2.55E-11
Liu 2012, 22961000	UK	3q25	*IL12A*	rs62270414	2861/8514	G/A	NA/0.15	1.41 (1.30, 1.53)	1.36E-16
Cordell 2021, 34033851	Mix_Europe	3q25	*IL12A*	rs589446	8021/16489	T/G	NA/NA	0.70 (0.67, 0.73)	6.15E-58
Liu 2012, 22961000	UK	3q25	*IL12A*	rs2366643	2861/8514	A/GC	NA/0.57	1.35 (1.27, 1.44)	7.65E-21
Liu 2012, 22961000	UK	3q25	*IL12A*	rs668998	2861/8514	G/A	NA/0.43	1.26 (1.17, 1.36)	1.74E-09
Mells 2011, 21399635	UK	3q25	*IL12A*	rs485499	1840/5163	T/C	0.57/0.65	1.38 (1.28, 1.50)	1.71E-15
Liu 2010, 20639880	Italy	3q25	*IL12A*	rs6441286	934/4651	G/T	0.49/0.44	1.44 (1.30, 1.60)	7.43E-12
Hirschfield 2010, 20639879	Mix_Europe	3q25	*IL12A*	rs6441286	1351/4700	G/T	0.5/0.39	1.44 (1.31, 1.57)	2.90E-15
Hirschfield_2009, 19458352	Canada, US	3q25.33	*IL12A*	rs6441286	1031/2713	G/T	0.5/0.39	1.54 (1.38, 1.72)	1.53E-14
Hirschfield_2009, 19458352	Canada, US	3q25.33	*IL12A*	rs574808	1031/2713	T/AC	0.68/0.57	0.65 (0.58, 0.73)	3.91E-13
Juran 2012, 22936693	Mix_Europe	3q25.33	*IL12A-AS1*	rs9877910	2216/5594	T/C	0.46/0.38	1.47 (1.34, 1.60)	1.65E-17
Cordell 2015, 26394269	Mix_Europe	3q25.33	*IL12A-AS1*	rs485499	2764/10475	C/T	NA/0.4	1.41 (1.32, 1.51)	1.49E-23
Hirschfield_2009, 19458352	Canada, US	3q26.1	*ARL14*	rs4679904	1031/2713	G/C	0.8/0.72	0.73 (0.64, 0.83)	1.22E-06
Cordell 2015, 26394269	Mix_Europe	4p16.3	*GAK*	rs11724804	4556/12990	A/G	NA/0.44	1.22 (1.12, 1.33)	9.01E-12
Hirschfield_2009, 19458352	Canada, US	4p15	*PCDH7*	rs10222962	505/1507	G/A	0.11/0.07	1.70 (1.33, 2.18)	2.56E-05
Cordell 2021, 34033851	Mix_Europe	4q24	*NFKB1*	rs7674640	8021/16489	C/T	NA/NA	0.81 (0.77, 0.84)	9.40E-23
Mells 2011, 21399635	UK	4q24	*NFKB1*	rs7665090	2460/7677	C/A	0.52/0.57	1.26 (1.18, 1.34)	1.04E-12
Liu 2012, 22961000	UK	4q24	*MANBA*	rs7665090	2861/8514	G/T	NA/0.52	1.26 (1.19, 1.34)	2.32E-14
Cordell 2015, 26394269	Mix_Europe	4q24	*MANBA*	rs1054037	2764/10475	T/C	NA/0.52	1.22 (1.15, 1.30)	8.27E-10
Cordell 2021, 34033851	Mix_Europe	4q24	*TET2*	rs7663401	8021/16489	C/T	NA/NA	0.88 (0.84, 0.92)	4.30E-08
Qiu 2017, 28425483	China	4q24	*NFKB1*	rs1598856	2029/6163	G/A	0.54/0.75	1.26 (1.17, 1.35)	2.44E-10
Hitomi 2024, 38652555	Japan	4q24	*MANBA*	rs223492	2181/2699	C/G	0.38/0.31	1.38 (1.27, 1.50)	1.87E-13
Hitomi 2019, 30643196	Japan	4q24	*NFKB1/MANBA*	rs17033015	1855/1719	A/C	NA/0.47	1.35 (1.23, 1.49)	9.00E-10
Qiu 2017, 28425483	China	4q27	*IL21*	rs925550	2029/6163	A/C	0.43/0.37	1.31 (1.21, 1.40)	3.95E-13
Qiu 2017, 28425483	China	4q27	*IL21*	rs17005934	2029/6163	G/A	0.41/0.35	1.29 (1.21, 1.39)	6.12E-13
Hirschfield_2009, 19458352	Canada, US	4q27	*TRPC3*	rs6838639	1031/2713	G/AC	0.8/0.73	1.59 (1.33, 1.92)	3.45E-07
Cordell 2021, 34033851	Mix_Europe	5p13.2	*IL7R*	rs35467801	8021/16489	GT/G	NA/NA	0.80 (0.76, 0.84)	3.25E-19
Hitomi 2024, 38652555	Japan	5p13.2	*IL7R*	rs11406102	2181/2699	GT/G	0.12/0.17	0.70 (0.62, 0.78)	1.48E-09
Hitomi 2019, 30643196	Japan	5p13.2	*IL7R/CAPSL*	rs12697352	1855/1719	G/A	NA/0.19	0.68 (0.60, 0.77)	2.00E-09
Kawashima 2017, 28062665	Japan	5p13.2	*IL7R*	rs6897932	1381/1505	T/C	0.14/0.2	1.52 (1.32, 1.75)	6.33E-09
Kawashima 2017, 28062665	Japan	5p13.2	*IL7R*	rs6890853	1381/1505	A/G	0.2/0.26	1.38 (1.22, 1.57)	4.09E-07

Nakamura 2012, 23000144	Japan	5p13.2	*IL7R*	rs6890853	1274/1091	A/G	0.24/0.26	1.47 (1.28, 1.69)	3.66E-08
Liu 2012, 22961000	UK	5p13.2	*IL7R*	rs6871748	2861/8514	A/T	NA/0.72	1.30 (1.21, 1.40)	1.77E-12
Mells 2011, 21399635	UK	5p13.2	*IL7R*	rs860413	2460/7677	A/CTG	0.72/0.77	1.30 (1.21, 1.40)	1.77E-12
Cordell 2015, 26394269	Mix_Europe	5p13.2	*IL7R*	rs860413	2764/10475	A/C	NA/0.79	1.28 (1.19, 1.38)	5.39E-11
Cordell 2015, 26394269	Mix_Europe	5q21.1	*PAM*	rs526231	6480/14736	T/C	NA/0.32	0.87 (0.81, 0.93)	1.14E-08
Cordell 2021, 34033851	Mix_Europe	5q33.3	*IL12B/RNF145*	rs2546890	8021/16489	G/A	NA/NA	0.87 (0.83, 0.90)	5.93E-11
Cordell 2015, 26394269	Mix_Europe	5q33.3	*RNF145*	rs2546890	6480/14736	G/A	NA/0.5	0.87 (0.82, 0.93)	1.06E-10
Cordell 2021, 34033851	Mix_Europe	7p14.1	*ELMO1*	rs60600003	8021/16489	G/T	NA/NA	1.29 (1.20, 1.38)	4.88E-13
Mells 2011, 21399635	UK	7p14.1	*ELMO1*	rs6974491	2460/7677	A/G	0.17/0.21	1.25 (1.16, 1.36)	3.82E-08
Cordell 2021, 34033851	Mix_Europe	7p21.1	*ITGB8*	rs7805218	8021/16489	A/G	NA/NA	1.14 (1.09, 1.19)	2.04E-08
Cordell 2021, 34033851	Mix_Europe	7q32.1	*IRF5/TNPO3*	rs12531711	8021/16489	G/A	NA/NA	1.52 (1.43, 1.62)	8.10E-42
Mells 2011, 21399635	UK	7q32.1	*IRF5/TNPO3*	rs12531711	1840/5163	G/A	0.11/0.16	1.58 (1.41, 1.76)	6.11E-16
Hirschfield 2010, 20639879	Mix_Europe	7q32.1	*IRF5-TNPO3*	rs10488631	1351/4700	C/T	0.17/0.12	1.57 (1.38, 1.77)	1.21E-12
Juran 2012, 22936693	Mix_Europe	7q32.1	*IRF5/TNPO3*	rs10488631	2216/5594	C/T	0.17/0.11	1.56 (1.38, 1.77)	2.49E-12
Liu 2010, 20639880	Italy	7q32.1	*IRF5/TNPO3*	rs10488631	934/4651	C/T	0.12/0.08	1.63 (1.40, 1.90)	2.63E-10
Hirschfield_2009, 19458352	Canada, US	7q32.1	*IRF5/TNPO3*	rs10488631	1031/2713	G/A	0.17/0.12	1.52 (1.30, 1.78)	1.76E-07
Cordell 2015, 26394269	Mix_Europe	7q32.1	*IRF5/TNPO3*	rs10488631	2764/10475	T/C	NA/0.91	1.59 (1.45, 1.74)	6.50E-23
Liu 2012, 22961000	UK	7q32.1	*IRF5/TNPO3*	rs35188261	2861/8514	A/G	NA/0.17	1.52 (1.39, 1.63)	6.69E-25
Liu 2012, 22961000	UK	7q32.1	*IRF5/TNPO3*	rs3807307	2861/8514	G/A	NA/0.47	1.22 (1.14, 1.30)	2.94E-09
Cordell 2021, 34033851	Mix_Europe	7q34	*ZC3HAV1L*	rs370193557	8021/16489	GAT/G	NA/NA	1.13 (1.08, 1.18)	2.93E-08
Cordell 2021, 34033851	Mix_Europe	9q22.33	*TRIM14*	rs11390003	8021/16489	GA/G	NA/NA	0.86 (0.82, 0.91)	3.42E-08
Qiu 2017, 28425483	China	9q32	*TNFSF8*	rs4979467	2029/6163	A/G	0.4/0.32	1.53 (1.42, 1.64)	5.61E-31
Hitomi 2024, 38652555	Japan	9q32	*TNFSF15*	rs4979462	2181/2699	T/C	0.58/0.47	1.62 (1.49, 1.76)	4.49E-31
Hitomi 2019, 30643196	Japan	9q32	*TNFSF15/TNFSF8*	rs4979462	1855/1719	C/T	NA/0.32	0.60 (0.55, 0.66)	2.00E-26
Kawashima 2017, 28062665	Japan	9q32	*TNFSF15*	rs4979462	1381/1505	T/C	0.42/0.54	1.60 (1.44, 1.78)	1.03E-18
Nakamura 2012, 23000144	Japan	9q32	*TNFSF15*	rs4979462	1274/1091	T/C	0.35/0.55	1.56 (1.39, 1.75)	2.84E-14
Hirschfield_2009, 19458352	Canada, US	9q34.3	*NOTCH1*	rs3124607	505/1507	G/ACT	0.73/0.67	1.45 (1.24, 1.70)	3.91E-06
Cordell 2021, 34033851	Mix_Europe	10q11.23	*WDFY4*	rs7097397	8021/16489	A/G	NA/NA	0.87 (0.83, 0.91)	3.83E-10
Cordell 2021, 34033851	Mix_Europe	11p15.5	*IRF7*	rs58523027	8021/16489	TAA/T	NA/NA	0.88 (0.85, 0.92)	2.26E-08
Cordell 2021, 34033851	Mix_Europe	11q13.1	*CCDC88B*	rs11601860	8021/16489	T/A	NA/NA	0.86 (0.83, 0.90)	2.18E-10
Mells 2011, 21399635	UK	11q13.1	*RPS6KA4*	rs538147	3394/12328	G/AT	0.61/0.65	1.23 (1.15, 1.31)	4.68E-10

Cordell 2015, 26394269	Mix_Europe	11q13.1	*CCDC88B*	rs510372	2764/10475	T/C	NA/0.43	0.81 (0.76, 0.87)	1.64E-09
Cordell 2021, 34033851	Mix_Europe	11q23.1	*POU2AF1*	rs12419634	8021/16489	G/C	NA/NA	0.88 (0.84, 0.92)	5.95E-09
Kawashima 2017, 28062665	Japan	11q23.1	*POU2AF1*	rs4938534	1381/1505	A/G	0.52/0.45	1.35 (1.22, 1.50)	1.49E-08
Nakamura 2012, 23000144	Japan	11q23.1	*POU2AF1*	rs4938534	1274/1091	A/G	0.53/0.42	1.39 (1.24, 1.56)	2.38E-08
Qiu 2017, 28425483	China	11q23.3	*CXCR5/DDX6*	rs77871618	2029/6163	A/G	0.23/0.16	1.40 (1.28, 1.53)	1.44E-13
Cordell 2021, 34033851	Mix_Europe	11q23.3	*CXCR/DDX6*	rs201150316	8021/16489	AT/A	NA/NA	0.69 (0.65, 0.73)	9.06E-35
Liu 2012, 22961000	UK	11q23.3	*DDX6*	rs80065107	2861/8514	A/T	NA/0.79	1.39 (1.28, 1.50)	3.99E-16
Juran 2012, 22936693	Mix_Europe	11q23.3	*CXCR/DDX6*	rs7117261	2216/5594	C/T	0.85/0.81	1.46 (1.30, 1.64)	1.71E-10
Mells 2011, 21399635	UK	11q23.3	*CXCR5/DDX6*	rs6421571	2460/7677	C/T	0.81/0.86	1.37 (1.25, 1.50)	1.30E-11
Cordell 2015, 26394269	Mix_Europe	11q23.3	*CXCR5/DDX6*	rs6421571	2764/10475	C/T	NA/0.8	1.39 (1.27, 1.52)	1.82E-13
Cordell 2021, 34033851	Mix_Europe	12p13.31	*TNFRSF1A*	rs1800693	8021/16489	C/T	NA/NA	1.20 (1.15, 1.25)	2.80E-16
Liu 2012, 22961000	UK	12p13.31	*TNFRSF1A*	rs1800693	2861/8514	G/A	NA/0.4	1.27 (1.19, 1.34)	2.97E-15
Mells 2011, 21399635	UK	12p13.31	*TNFRSF1A*	rs1800693	2460/7677	C/T	0.4/0.45	1.22 (1.14, 1.30)	2.94E-09
Cordell 2015, 26394269	Mix_Europe	12p13.31	*TNFRSF1A*	rs1800693	2764/10475	T/C	NA/0.49	1.22 (1.14, 1.30)	1.74E-09
Liu 2012, 22961000	UK	12p13.31	*TNFRSF1A*	rs11064157	2861/8514	A/C	NA/0.25	1.23 (1.15, 1.32)	3.96E-09
Qiu 2017, 28425483	China	12p13.31	*NFKB1*	rs4149576	2029/6163	A/G	0.15/0.11	1.37 (1.23, 1.52)	5.56E-09
Cordell 2021, 34033851	Mix_Europe	12q24.12	*SH2B3/ATXN2*	rs35350651	8021/16489	AC/A	NA/NA	0.83 (0.79, 0.86)	9.44E-20
Liu 2012, 22961000	UK	12q24.12	*ATXN2*	rs11065979	2861/8514	A/G	NA/0.44	1.20 (1.13, 1.27)	9.41E-10
Cordell 2015, 26394269	Mix_Europe	12q24.12	*ATXN2*	rs11065987	2764/10475	A/G	NA/0.63	0.84 (0.79, 0.89)	3.27E-08
Cordell 2021, 34033851	Mix_Europe	13q14	*TNFSF11*	rs9533122	8021/16489	A/G	NA/NA	0.86 (0.82, 0.89)	1.85E-12
Juran 2012, 22936693	Mix_Europe	13q14	*LINC02341/TNFSF11*	rs3862738	2216/5594	G/A	0.73/0.78	1.33 (1.20, 1.47)	3.62E-08
Cordell 2021, 34033851	Mix_Europe	13q14	*DLEU1*	rs9591325	8021/16489	C/T	NA/NA	0.64 (0.58, 0.70)	1.57E-19
Cordell 2015, 26394269	Mix_Europe	13q14	*DLEU1*	rs9591325	2764/10475	T/C	NA/0.94	1.63 (1.41, 1.89)	1.14E-10
Hirschfield_2009, 19458352	Canada, US	13q33.3	*FAM155A*	rs2211312	1031/2713	A/C	0.93/0.88	1.75 (1.33, 2.33)	5.08E-05
Cordell 2021, 34033851	Mix_Europe	14q24	*RAD51B*	rs3784099	8021/16489	A/G	NA/NA	0.82 (0.78, 0.86)	2.71E-17
Liu 2012, 22961000	UK	14q24	*RAD51B*	rs911263	2861/8514	A/C	NA/0.71	1.26 (1.17, 1.35)	2.44E-10
Mells 2011, 21399635	UK	14q24	*RAD51B*	rs911263	2460/7677	T/G	0.71/0.76	1.29 (1.20, 1.39)	1.11E-11
Cordell 2015, 26394269	Mix_Europe	14q24	*RAD51B*	rs911263	2764/10475	T/C	NA/0.73	1.24 (1.16, 1.33)	2.21E-09
Cordell 2021, 34033851	Mix_Europe	14q32.12	*RIN3*	rs72699866	8021/16489	A/G	NA/NA	0.82 (0.78, 0.87)	1.77E-11
Cordell 2021, 34033851	Mix_Europe	14q32.32	*TNFAIP2*	rs59643720	8021/16489	C/A	NA/NA	1.37 (1.31, 1.44)	1.37E-39
Mells 2011, 21399635	UK	14q32.32	*TNFAIP2*	rs8017161	3394/12328	A/G	0.4/0.44	1.22 (1.16, 1.27)	7.70E-18

Cordell 2015, 26394269	Mix_Europe	14q32.32	*EXOC3L4*	rs2297067	2764/10475	C/T	NA/0.82	0.72 (0.67, 0.77)	4.48E-19
Qiu 2017, 28425483	China	15q25.1	*IL16*	rs11556218	2029/6163	C/T	0.23/0.19	1.29 (1.18, 1.41)	2.08E-08
Kawashima 2017, 28062665	Japan		*PRKCB*	rs7404928	1893/8017	T/C	0.67/0.61	1.25 (1.09, 1.43)	4.13E-09
Cordell 2021, 34033851	Mix_Europe	16p12.1	*IL21R*	rs1119132	8021/16489	A/G	NA/NA	0.82 (0.77, 0.87)	7.67E-10
Qiu 2017, 28425483	China	16p12.1	*IL4R/IL21R*	rs2189521	2029/6163	G/C	0.24/0.31	0.71 (0.66, 0.78)	9.23E-16
Qiu 2017, 28425483	China	16p12.1	*IL4R/IL21R*	rs10852316	2029/6163	A/C	0.38/0.24	0.76 (0.71, 0.82)	8.10E-14
Cordell 2021, 34033851	Mix_Europe	16p13.13	*CLEC16A*	rs9652601	8021/16489	A/G	NA/NA	0.79 (0.75, 0.82)	1.52E-23
Liu 2012, 22961000	UK	16p13.13	*CLEC16A*	rs12708715	2861/8514	C/T	NA/0.68	1.29 (1.21, 1.38)	3.13E-14
Cordell 2015, 26394269	Mix_Europe	16p13.13	*CLEC16A*	rs12924729	2764/10475	G/A	NA/0.67	1.31 (1.22, 1.40)	2.46E-14
Mells 2011, 21399635	UK	16p13.13	*CLEC16A*	rs12924729	2460/7677	G/A	0.68/0.74	1.29 (1.20, 1.38)	9.19E-13
Liu 2012, 22961000	UK	16p13	*SOCS1/RMI2*	rs1646019	2861/8514	G/A	NA/0.71	1.31 (1.23, 141)	6.72E-15
Juran 2012, 22936693	Mix_Europe	16p13	*RMI2*	rs413024	2216/5594	T/C	0.74/0.71	1.31 (1.19, 1.44)	2.84E-08
Liu 2012, 22961000	UK	16p13	*SOCS1/RMI2*	rs80073729	2861/8514	A/G	NA/0.004	2.96 (2.02, 4.33)	2.42E-08
Qiu 2017, 28425483	China	16q21	*CCDC113*	rs2550374	2029/6163	A/G	0.42/0.48	0.81 (0.76, 0.87)	9.91E-10
Cordell 2021, 34033851	Mix_Europe	16q22.1	*DPEP3*	rs79577483	8021/16489	G/A	NA/NA	1.24 (1.16, 1.31)	7.99E-12
Cordell 2021, 34033851	Mix_Europe	16q24	*IRF8*	rs11117432	8021/16489	A/G	NA/NA	0.76 (0.72, 0.80)	4.93E-24
Liu 2012, 22961000	UK	16q24	*IRF8*	rs11117433	2861/8514	G/ACT	NA/0.77	1.26 (1.17, 1.36)	1.74E-09
Mells 2011, 21399635	UK	16q24	*LOC124903741*	rs11117432	2460/7677	G/ACT	0.76/0.81	1.31 (1.21, 1.43)	2.35E-10
Cordell 2021, 34033851	Mix_Europe	17q12	*IKZF3*	rs33938760	8021/16489	CTT/C	NA/NA	0.77 (0.74, 0.80)	1.83E-32
Cordell 2015, 26394269	Mix_Europe	17q12	*IKZF3*	rs9303277	2764/10475	C/T	NA/0.5	1.24 (1.16, 1.31)	2.42E-11
Liu 2010, 20639880	Italy	17q12	*IKZF3*	rs9303277	934/4651	T/C	0.53/0.45	1.38 (1.24, 1.53)	1.54E-09
Qiu 2017, 28425483	China	17q12	*IKZF3*	rs9635726	2029/6163	G/C	0.38/0.31	1.37 (1.27, 1.48)	7.36E-16
Hitomi 2024, 38652555	Japan	17q12	*ZPBP2*	rs200216139	2181/2699	GC/G	0.3/0.23	1.48 (1.34, 1.62)	3.43E-16
Hitomi 2019, 30643196	Japan	17q12	*IKZF3*	rs4795395	1855/1719	AG/T	NA/0.35	1.42 (1.29, 1.57)	4.00E-12
Kawashima 2017, 28062665	Japan	17q12	*IKZF3*	rs9303277	1381/1505	T/C	0.39/0.31	1.43 (1.29, 1.60)	8.78E-11
Nakamura 2012, 23000144	Japan	17q12	*IKZF3*	rs9303277	1274/1091	T/C	0.37/0.3	1.44 (1.28, 1.63)	3.66E-09
Hirschfield_2009, 19458352	Canada, US	17q12	*IKZF3*	rs9303277	505/1507	A/G	0.57/0.5	1.41 (1.22, 1.66)	1.22E-05
Hirschfield 2010, 20639879	Mix_Europe	17q12	*IKZF3*	rs907092	1351/4700	A/G	0.53/0.45	1.33 (1.22, 1.45)	9.63E-11
Hirschfield_2009, 19458352	Canada, US	17q12	*IKZF3*	rs907092	1031/2713	A/G	0.52/0.45	1.29 (1.15, 1.44)	9.05E-06
Juran 2012, 22936693	Mix_Europe	17q12	*IKZF3*	rs907091	2216/5594	C/T	0.56/0.52	1.29 (1.19, 1.41)	4.00E-09
Liu 2012, 22961000	UK	17q12	*ORMDL3*	rs8067378	2861/8514	G/A	NA/0.52	1.26 (1.19, 1.34)	2.32E-14

Mells 2011, 21399635	UK	17q12	*ORMDL3*	rs7208487	1840/5163	T/CG	0.84/0.87	1.32 (1.18, 1.48)	7.89E-07
Hirschfield_2009, 19458352	Canada, US	17q12	*GSDMB*	rs2305480	505/1507	A/G	0.51/0.44	1.38 (1.19, 1.60)	2.00E-05
Hirschfield 2010, 20639879	Mix_Europe	17q12.21	*ZPBP2*	rs11557467	1351/4700	G/T	0.43/0.51	0.72 (0.66, 0.79)	7.93E-13
Cordell 2021, 34033851	Mix_Europe	17q21.31	*MAPT*	rs17564829	8021/16489	C/A	NA/NA	0.84 (0.80, 0.89)	3.71E-11
Liu 2012, 22961000	UK	17q21.31	*MAPT*	rs17564829	2861/8514	G/A	NA/0.24	1.25 (1.16, 1.35)	8.08E-09
Hirschfield_2009, 19458352	Canada, US	18q21	*CCDC68*	rs9964104	1031/2713	A/T	0.69/0.61	0.80 (0.71, 0.90)	2.77E-05
Cordell 2021, 34033851	Mix_Europe	18q22.2	*CD226*	rs1808094	8021/16489	T/C	NA/NA	1.14 (1.09, 1.18)	1.09E-09
Hitomi 2024, 38652555	Japan	18p11.21	*PTPN2*	rs8098858	2181/2699	A/C	0.22/0.18	1.34 (1.21, 1.48)	2.56E-08
Liu 2012, 22961000	UK	19p12	*TYK2*	rs34536443	2861/8514	G/CT	NA/0.95	1.91 (1.59, 2.28)	1.96E-12
Cordell 2021, 34033851	Mix_Europe	19p13.2	*TYK2*	rs2304256	8021/16489	A/C	NA/NA	0.81 (0.78, 0.85)	1.32E-17
Cordell 2015, 26394269	Mix_Europe	19p13.2	*TYK2*	rs2304256	2764/10475	A/C	NA/0.29	0.79 (0.74, 0.85)	1.47E-10
Cordell 2021, 34033851	Mix_Europe	19q13	*SPIB*	rs3745516	8021/16489	A/G	NA/NA	1.32 (1.25, 1.38)	3.45E-30
Mells 2011, 21399635	UK	19q13	*SPIB*	rs3745516	1840/5163	A/GT	0.23/0.29	1.38 (1.32, 1.44)	1.04E-47
Liu 2010, 20639880	Italy	19q13	*SPIB*	rs3745516	934/4651	A/G	0.36/0.27	1.46 (1.30, 1.64)	7.11E-11
Cordell 2015, 26394269	Mix_Europe	19q13	*SPIB*	rs3745516	2764/10475	G/A	NA/0.76	1.39 (1.30, 1.49)	1.13E-20
Qiu 2017, 28425483	China	19p13.3	*ARID3A*	rs10415976	2029/6163	G/A	0.41/0.49	0.77 (0.72, 0.84)	3.00E-11
Qiu 2017, 28425483	China	19p13.3	*ARID3A*	rs10414193	2029/6163	G/A	0.4/0.47	0.79 (0.73, 0.85)	1.27E-09
Hirschfield_2009, 19458352	Canada, US	20p13	*SLC52A3*	rs6140113	1031/2713	G/A	0.89/0.84	1.61 (1.30, 2.04)	3.75E-05
Cordell 2021, 34033851	Mix_Europe	22q13	*SYNGR1*	rs137687	8021/16489	A/G	NA/NA	0.80 (0.77, 0.84)	3.80E-23
Liu 2012, 22961000	UK	22q13	*SYNGR1*	rs2267407	2861/8514	A/G	NA/0.23	1.29 (1.21, 1.38)	3.13E-14
Juran 2012, 22936693	Mix_Europe	22q13	*SYNGR1*	rs715505	2216/5594	C/G	0.28/0.23	1.41 (1.28, 1.60)	1.58E-09
Cordell 2015, 26394269	Mix_Europe	22q13	*SYNGR1*	rs2069235	2764/10475	G/A	NA/0.68	0.79 (0.74, 0.85)	2.10E-11
Qiu 2017, 28425483	China	22q13	*RPL3/SYNGR1*	rs137603	2029/6163	C/A	0.11/0.15	0.73 (0.65, 0.81)	2.07E-08
Mells 2011, 21399635	UK	22q13	*MAP3K7IP1*	rs968451	2460/7677	T/G	0.19/0.23	1.27 (1.18, 1.38)	2.17E-09
**HLA-region genes**									
Wang 2019, 31810856	China	6p21	*HLA-DRB1*	rs16822805	1126/1770	G/C	0.32/0.22	1.70 (1.51, 1.92)	4.75E-18
Wang 2019, 31810856	China	6p21	*HLA-DRB1*	rs17886882	1126/1770	A/P	0.38/0.51	0.58 (0.52, 0.65)	1.08E-21
Qiu 2017, 28425483	China	6p21	*HLA-DRA*	rs9268644	2029/6163	G/T	0.1/0.18	0.51 (0.45, 0.57)	7.83E-31
Qiu 2017, 28425483	China	6p21	*HLA-DRA*	rs9501251	2029/6163	A/G	0.08/0.04	2.01 (1.76, 2.32)	2.10E-22
Nakamura 2012, 23000144	Japan	6p21	*HLA-DQB1*	rs9275175	487/476	A/G	0.37/0.53	1.94 (1.62, 2.33)	8.30E-13
Hitomi 2024, 38652555	Japan	6p21	*HLA-DRA*	rs9268641	2181/2699	C/T	0.12/0.22	0.46 (0.41, 0.52)	1.49E-37

Kawashima 2017, 28062665	Japan	6p21	*HLA-DRA*	rs3129887	1893/8017	A/G	NA/NA	3.13 (2.54, 3.85)	2.04E−29
Invernizzi 2012, 22573116	Italy	6p21	*HLA*	rs114327274	676/1440	A/G	0.24/0.17	1.66 (1.39, 1.99)	4.04E-08
Liu 2010, 20639880	Italy	6p21.3	*C6orf10*	rs2395148	934/4651	T/G	0.06/0.02	2.75 (2.13, 3.55)	9.71E-15
Hirschfield_2009, 19458352	Canada, US	6p21.3	*C6orf10*	rs2395148	1031/2713	A/C	0.06/0.02	2.87 (2.16, 3.82)	4.20E-13
Liu 2010, 20639880	Italy	6p21.3	*BTNL2*	rs3806156	934/4651	T/G	0.37/0.32	1.42 (1.28, 1.58)	1.66E-10
Hirschfield_2009, 19458352	Canada, US	6p21.3	*BTNL2*	rs3806156	1031/2713	A/G	0.46/0.35	1.42 (1.27, 1.58)	3.10E-10
Invernizzi 2012, 22573116	Italy	6p21.3	*BTNL2*	rs116348417	676/1440	A/G	0.26/0.37	0.66 (0.57, 0.77)	4.90E-08
Hirschfield_2009, 19458352	Canada, US	6p21.3	*BTNL2*	rs3135363	505/1507	A/CGT	0.78/0.72	1.56 (1.30, 1.85)	4.46E-08
Hirschfield_2009, 19458352	Canada, US	6p21	*HLA-DRB1*	rs660895	505/1507	G/A	0.28/0.2	1.60 (1.35, 1.90)	4.68E-08
Hirschfield_2009, 19458352	Canada, US	6p21	*HLA-DQB1*	rs2856683	1031/2713	C/T	0.36/0.22	1.75 (1.55, 1.98)	3.26E-19
Juran 2012, 22936693	Mix_Europe	6p21.32	*HLA-DQB1/HLA-DQA2*	rs7775055	2216/5594	C/T	0.08/0.02	3.71 (3.00, 4.59)	1.27E-33
Invernizzi 2012, 22573116	Italy	6p21.32	*HLA-DQB1/HLA-DQA2*	rs115721871	676/1440	A/G	0.17/0.08	2.61 (2.08, 3.27)	1.00E-16
Invernizzi 2012, 22573116	Italy	6p21.32	*HLA-DQB1/HLA-DQA2*	rs4246055	676/1440	C/T	0.19/0.1	2.47 (1.99, 3.07)	2.87E-16
Cordell 2021, 34033851	Mix_Europe	6p21.32	*HLA-DQB1/HLA-DQA2*	rs7774434	8021/16489	C/T	NA/NA	1.60 (1.53, 1.67)	2.91E-101
Mells 2011, 21399635	UK	6p21.32	*HLA-DQB1/HLA-DQA2*	rs7774434	1840/5163	C/T	0.38/0.49	1.60 (1.30, 1.90)	3.86E-34
Cordell 2015, 26394269	Mix_Europe	6p21.32	*HLA-DQB1/HLA-DQA2*	rs7774434	2764/10475	C/T	NA/0.47	1.68 (1.58, 1.79)	6.30E-56
Liu 2012, 22961000	UK	6p21.32	*HLA-DQB1/HLA-DQA2*	rs7774434	2861/8514	C/T	0.38/NA	1.57 (1.48, 1.67)	2.06E-48
Invernizzi 2012, 22573116	Italy	6p21.32	*HLA-DQB1/HLA-DQA2*	rs114183935	676/1440	A/G	0.59/0.43	1.80 (1.56, 2.08)	8.30E-16
Invernizzi 2012, 22573116	Italy	6p21.32	*HLA-DQB1/HLA-DQA2*	rs114432443	676/1440	T/C	0.39/0.53	0.55 (0.47, 0.64)	4.30E-15
Liu 2010, 20639880	Italy	6p21.32	*HLA-DQB1/HLA-DQA2*	rs9275424	934/4651	G/A	0.26/0.18	1.70 (1.52, 1.91)	1.58E-19
Hirschfield_2009, 19458352	Canada, US	6p21.32	*HLA-DQB1/HLA-DQA2*	rs9275390	505/1507	G/T	0.37/0.25	1.81 (1.55, 2.11)	4.67E-14
Hirschfield_2009, 19458352	Canada, US	6p21.32	*HLA-DQB1/HLA-DQA2*	rs7775228	505/1507	G/T	0.21/0.12	1.87 (1.54, 2.27)	2.55E-10
Invernizzi 2012, 22573116	Italy	6p21.32	*HLA-DQB1/HLA-DQA2*	rs114796881	676/1440	A/G	0.3/0.41	0.63 (0.54, 0.73)	1.12E-09
Invernizzi 2012, 22573116	Italy	6p21.32	*HLA-DQB1/HLA-DQA2*	rs116493712	676/1440	T/C	0.57/0.46	1.56 (1.35, 1.80)	1.12E-09
Hirschfield_2009, 19458352	Canada, US	6p21.32	*HLA-DQB1/HLA-DQA2*	rs9275312	505/1507	G/A	0.23/0.13	2.01 (1.66, 2.42)	3.87E-13
Hirschfield 2010, 20639879	Mix_Europe	6p21	*HLA - DPB1*	rs9277535	1351/4700	G/A	0.33/0.24	1.51 (1.37, 1.66)	3.98E-17
Hirschfield_2009, 19458352	Canada, US	6p21	*HLA-DPB1*	rs9277535	1031/2713	G/A	0.33/0.24	1.50 (1.33, 1.70)	9.45E-11
Liu 2010, 20639880	Italy	6p21	*COL11A2*	rs2855430	934/4651	A/G	0.13/0.1	1.53 (1.32, 1.77)	9.56E-09
Cordell 2021, 34033851	Mix_Europe	6q23.3	*TNFAIP3*	rs2327832	8021/16489	G/A	NA/NA	1.17 (1.12, 1.23)	1.19E-10
Cordell 2015, 26394269	Mix_Europe	6q23.3	*LOC102723649*	rs6933404	6480/14736	C/T	NA/0.17	1.18 (1.09, 1.27)	1.27E-10
Wang 2019, 31810856	China	6p21	*HLA-DQB1*	DQB1*03:01	1126/1770	P/A	0.12/0.21	0.52 (0.45, 0.61)	3.57E-17

Liu 2012, 22961000	UK	6p21	*HLA-DQB1*	DQB1*03:01	2861/8514	NA	0.13/0.18	0.70 (0.64, 0.77)	6.48E-14
Invernizzi 2012, 22573116	Italy	6p21	*HLA-DQB1*	DQB1*03:01	676/1440	SPEC/ OTHER	0.21/0.31	0.61 (0.52, 0.72)	6.10E-09
Liu 2012, 22961000	UK	6p21	*HLA-DQB1*	DQB1*06:02	2861/8514	NA	0.09/0.13	0.64 (0.57, 0.72)	2.32E-15
Invernizzi 2012, 22573116	Italy	6p21	*HLA-DQB1*	DQB1*04:02	676/1440	SPEC/ OTHER	0.07/0.03	3.16 (2.22, 4.49)	1.40E-10
Liu 2012, 22961000	UK	6p21	*HLA-DQA1*	DQA1*04:01	2861/8514	NA	0.06/0.02	3.06 (2.62, 3.58)	5.90E-45
Invernizzi 2012, 22573116	Italy	6p21	*HLA-DQA1*	DQA1*04:01	676/1440	SPEC/O THER	0.07/0.02	0.32 (0.23, 0.45)	1.90E-10
Wang 2019, 31810856	China	6p21	*HLA-DQA1*	DQA1*05:05	1126/1770	P/A	0.02/0.11	0.52 (0.43, 0.64)	1.15E-10
Wang 2019, 31810856	China	6p21	*HLA-DPB1*	DPB1*17:01	1126/1770	P/A	0.07/0.03	2.43 (1.88, 3.13)	8.62E-12
Invernizzi 2012, 22573116	Italy	6p21	*HLA-DRB1*	DRB1*11	676/1440	SPEC/ OTHER	0.15/0.26	0.55 (0.46, 0.66)	1.40E-10
Wang 2019, 31810856	China	6p21	*HLA-DRB1*	DRB1*11:01	1126/1770	P/A	0.03/0.07	0.47 (0.36, 0.62)	1.39E-08
Invernizzi 2012, 22573116	Italy	6p21	*HLA-DRB1*	DRB1*08	676/1440	SPEC/ OTHER	0.08/0.03	3.22 (2.29, 4.53)	1.60E-11
Wang 2019, 31810856	China	6p21	*HLA-DRB1*	DRB1*08:03	1126/1770	P/A	0.13/0.08	1.64 (1.38, 1.95)	2.04E-08
Liu 2012, 22961000	UK	6p21	*HLA-DRB1*	DRB1*04:04	2861/8514	NA	0.07/0.05	1.57 (1.36, 1.82)	1.22E-09
Wang 2019, 31810856	China	6p21	*HLA-DPA1*	DPA1*01:03	1126/1770	P/A	0.31/0.38	0.71 (0.64, 0.80)	1.78E-09

Supplementary Table 3. Characteristics of the the candidate-gene association studies on variants in Non-HLA genes and risk of primary biliary cirrhosis in the meta-analysis.

Study, PMID	Gene	Variant	Country/ Region	A1/ A2	Cases/ Controls	OR (95%CI)	Participants
Li 2013, 23432218	*CTLA-4*	rs231775	China	A/G	312/375	1.44 (1.24, 1.67)	Female PBC patients from Chinese Han population who had received treatment at three different centers since 2006
Fan 2004, 15378793	*CTLA-4*	rs231775	China	A/G	77/160	1.80 (1.20, 2.72)	Participants were enrolled into the study from Eastern China, including Shanghai, Zhejiang and Jiangsu province, China
Aiba 2011, 21594562	*CTLA-4*	rs231775	Japan	A/G	450/371	1.35 (1.10, 1.65)	The PBC Study Group in National Hospital Organization Study Group for Liver Disease in Japan (NHOSLJ) and the Shinshu PBC Study Group in Japan
Joshita 2010, 20557968	*CTLA-4*	rs231775	Japan	A/G	198/170	1.30 (0.96, 1.75)	Shinshu University Hospital
Joshita 2010, 20557968	*CTLA-4*	rs231775	Japan	A/G	110/98	1.10 (0.74, 1.63)	National Hospital Organization Nagasaki Medical Center
Walker 2009, 19333938	*CTLA-4*	rs231775	Canada	A/G	481/1248	1.28 (1.09, 1.50)	Unrelated Caucasian participants recruited from Toronto General Hospital and Mount Sinai Hospital
Poupon 2008, 18930330	*CTLA-4*	rs231775	France	A/G	258/286	1.60 (1.24, 2.07)	French white continental patients with French ancestry recruited specifically for genetic studies
Schott 2007, 18049163	*CTLA-4*	rs231775	German	A/G	180/202	1.32 (0.98, 1.78)	Patients were German descent (71.6%), Turkish or Arabicases (8.2%) and other Caucasian descent (5.9%)
Donaldson 2007, 17482523	*CTLA-4*	rs231775	Italy	A/G	80/99	0.88 (0.54, 1.43)	Participants were recruited from Padova area of northern Italy
Donaldson 2007, 17482523	*CTLA-4*	rs231775	UK	A/G	247/292	1.13 (0.89, 1.45)	Participants were recruited from Newcastle area of UK
Mantaka 2012, 22609442	*CTLA-4*	rs231775	Greece	A/G	100/158	1.13 (0.79, 1.61)	PBC patients originated from Crete, who attended the Liver Clinic of the Department of Gastroenterology of the University Hospital of Heraklion between September 1989 and May 2010
Juran 2008, 18778710	*CTLA-4*	rs231775	USA	A/G	351/279	1.19 (0.95, 1.49)	Recruited into Mayo Clinic PBC Genetic Epidemiology Registry and Biospecimen Repository[†]
Juran 2007, 18041714	*CTLA-4*	rs231775	USA	A/G	351/205	1.11 (0.86, 1.42)	Recruited into Mayo Clinic PBC Genetic Epidemiology Registry and Biospecimen Repository[†]
Li 2013, 23432218	*CTLA-4*	rs5742909	China	C/T	312/375	0.93 (0.68, 1.27)	Female PBC patients from Chinese Han population who had received treatment at three different centers since 2006
Fan 2004, 15378793	*CTLA-4*	rs5742909	China	C/T	77/160	0.69 (0.38, 1.26)	Participants were enrolled into the study from Eastern China, including Shanghai, Zhejiang and Jiangsu province, China
Joshita 2010, 20557968	*CTLA-4*	rs5742909	Japan	C/T	198/170	0.79 (0.49, 1.29)	Shinshu University Hospital
Joshita 2010, 20557968	*CTLA-4*	rs5742909	Japan	C/T	110/98	0.95 (0.54, 1.67)	National Hospital Organization Nagasaki Medical Center
Walker 2009, 19333938	*CTLA-4*	rs5742909	Canada	C/T	481/1248	0.69 (0.54, 0.89)	Unrelated Caucasian participants recruited from Toronto General Hospital and Mount Sinai Hospital
Poupon 2008, 18930330	*CTLA-4*	rs5742909	France	C/T	258/286	0.66 (0.43, 1.00)	French white continental patients with French ancestry recruited specifically for genetic studies
Juran 2008, 18778710	*CTLA-4*	rs5742909	US	C/T	351/279	0.78 (0.53, 1.15)	Recruited into Mayo Clinic PBC Genetic Epidemiology Registry and Biospecimen Repository
Schott 2007, 18049163	*CTLA-4*	rs5742909	German	C/T	180/202	0.70 (0.49, 0.98)	Patients were German descent (71.6%), Turkish or Arabicases (8.2%) and other Caucasian descent (5.9%)
Li 2013, 23432218	*CTLA-4*	rs3087243	China	G/A	312/375	0.86 (0.68, 1.07)	Female PBC patients from Chinese Han population who had received treatment at three different centers since 2006
Aiba 2011, 21594562	*CTLA-4*	rs3087243	Japan	G/G	450/371	0.68 (0.55, 0.85)	The PBC Study Group in National Hospital Organization Study Group for Liver Disease in Japan (NHOSLJ) and the Shinshu PBC Study Group in Japan
Joshita 2010, 20557968	*CTLA-4*	rs3087243	Japan	G/A	198/170	0.82 (0.59, 1.13)	Shinshu University Hospital

Joshita 2010, 20557968	*CTLA-4*	rs3087243	Japan	G/A	110/98	0.79 (0.52, 1.21)	National Hospital Organization Nagasaki Medical Center
Walker 2009, 19333938	*CTLA-4*	rs3087243	Canada	G/A	481/1248	0.69 (0.58, 0.81)	Unrelated Caucasian participants recruited from Toronto General Hospital and Mount Sinai Hospital
Donaldson 2007, 17482523	*CTLA-4*	rs3087243	UK	G/A	247/292	0.91 (0.70, 1.18)	Participants were recruited from Newcastle area of UK
Mantaka 2012, 22609442	*CTLA-4*	rs3087243	Greece	G/A	100/158	0.87 (0.61, 1.24)	PBC patients originated from Crete, who attended the Liver Clinic of the Department of Gastroenterology of the University Hospital of Heraklion between September 1989 and May 2010
Juran 2008, 18778710	*CTLA-4*	rs3087243	USA	G/A	351/279	0.96 (0.77, 1.20)	Recruited into Mayo Clinic PBC Genetic Epidemiology Registry and Biospecimen Repository†
Juran 2007, 18041714	*CTLA-4*	rs3087243	USA	G/A	351/205	1.00 (0.79, 1.28)	Recruited into Mayo Clinic PBC Genetic Epidemiology Registry and Biospecimen Repository†
Li 2013, 23432218	*CTLA-4*	rs231725	China	G/A	312/375	1.29 (1.12, 1.48)	Female PBC patients from Chinese Han population who had received treatment at three different centers since 2006
Aiba 2011, 21594562	*CTLA-4*	rs231725	Japan	G/A	450/371	1.37 (1.13, 1.67)	The PBC Study Group in National Hospital Organization Study Group for Liver Disease in Japan (NHOSLJ) and the Shinshu PBC Study Group in Japan
Joshita 2010, 20557968	*CTLA-4*	rs231725	Japan	G/A	198/170	1.32 (0.99, 1.77)	Shinshu University Hospital
Joshita 2010, 20557968	*CTLA-4*	rs231725	Japan	G/A	110/98	1.21 (0.82, 1.78)	National Hospital Organization Nagasaki Medical Center
Juran 2008, 18778710	*CTLA-4*	rs231725	US	G/A	351/279	1.38 (1.09, 1.75)	Recruited into Mayo Clinic PBC Genetic Epidemiology Registry and Biospecimen Repository
Joshita 2010, 20557968	*CTLA-4*	rs733618	Japan	/C	198/170	1.25 (0.93, 1.67)	Shinshu University Hospital
Joshita 2010, 20557968	*CTLA-4*	rs733618	Japan	/C	110/98	0.85 (0.58, 1.26)	National Hospital Organization Nagasaki Medical Center
Donaldson 2007, 17482523	*CTLA-4*	rs733618	UK	A/G	247/292	1.27 (0.82, 1.97)	Participants were recruited from Newcastle area of UK
Wasik 2017, 28299343	*IL12RB2*	rs3790567	Poland	G/A	306/258	1.70 (1.30, 2.20)	A homogenous group of Caucasian patients with PBC who were treated in two Polish liver transplant centres, in Szczecin and in Warsaw
Paziewska 2017, 28056976	*IL12RB2*	rs3790567	Poland	G/A	443/934	1.37 (1.14, 1.65)	Polish Caucasians were recruited at nine recruitment centers
Joshita 2014, 24648611	*IL12RB*	rs3790567	Japan	G/A	395/458	1.16 (0.93, 1.45)	Enrolled at Shinshu University Hospital and the National Hospital Organization Nagasaki Medical Center, Japan
Tanaka 2011, 21506939	*IL12RB2*	rs3790567	Japan	G/A	303/298	0.94 (0.73, 1.23)	Patients were recruited at several referral hospitals in Japan
Dong 2015, 25690649	*17q12*	rs9303277	China	C/T	1070/1198	1.38 (1.22, 1.56)	Genetically unrelated PBC patients in South China (mainly in Shanghai and Jiangsu Province)
Tanaka 2011, 21506939	*IKZF3*	rs9303277	Japan	C/T	303/298	1.45 (1.14, 1.86)	Patients were recruited at several referral hospitals in Japan
Paziewska 2017, 28056976	*IKZF3*	rs9303277	Poland	C/T	443/934	1.27 (1.07, 1.50)	Polish Caucasians were recruited at nine recruitment centers
Niro 2009, 19758199	*TNF-α*	rs1800629	Italy	G/A	52/61	1.19 (0.45, 3.12)	One medical center located in San Giovanni Rotondo in the south of Italy
Niro 2009, 19758199	*TNF-α*	rs1800629	Italy	G/A	55/80	0.70 (0.30, 1.63)	One medical center located in Milan in the North of Italy
Poupon 2008, 18930330	*TNF-α*	rs1800629	France	G/A	258/286	0.92 (0.64, 1.33)	French white continental patients with French ancestry recruited specifically for genetic studies
Gordon 1999, 10453936	*TNF-α*	rs1800629	England	G/2	91/209	0.66 (0.42, 1.04)	England Caucasian patients with PBC were recruited, and the control group were unrelated North of England Caucasians with no family history of autoimmune disease
Jones 1999, 10068101	*TNF-α*	rs1800629	England	G/2	168/145	0.74 (0.50, 1.09)	Unrelated PBC patients from the North-East of England attending the Freeman Hospital PBC clinic
Bittencourt 2003, 12911663	*TNF-α*	rs1800629	Brazil	G/2	57/83	0.59 (0.26, 1.34)	Participant were from the metropolitan area of S ão Paulo, Brazil, were studied
Gervais 2021, 33833419	*STAT4*	rs7574865	Japan	G/T	220/271	1.44 (1.08, 1.91)	Japan PBC-GWAS Consortium

Joshita 2014, 24648611	*STAT4*	rs7574865	Japan	G/T	395/458	1.43 (1.17, 1.74)	Enrolled at Shinshu University Hospital and the National Hospital Organization Nagasaki Medical Center, Japan
Dong 2015, 25690649	*STAT4*	rs7574865	China	G/T	1070/1198	1.23 (1.09, 1.39)	Genetically unrelated PBC patients in South China (mainly in Shanghai and Jiangsu Province)
Kempinska 2012, 22690210	*VDR*	rs1544410	Poland	G/A	143/306	1.69 (1.27, 2.24)	Cases were from Poland, and controls were umbilical cord blood samples from healthy newborn children
Tanaka2009, 19376604	*VDR*	rs1544410	Italy	G/A	139/156	1.54 (1.10, 2.16)	Patients were recruited at one center in Italy
Vogel 2002, 11786968	*VDR*	rs1544410	German	G/A	74/214	1.38 (0.94, 2.02)	Cases were from Germany and an autoimmune disease-free control group consisted of Northern German white patients
Tanaka2009, 19376604	*VDR*	rs1544410	Japan	G/A	195/179	1.74 (1.13, 2.67)	Patients were recruited at several referral hospitals in Japan
Fan 2005, 15683428	*VDR*	rs1544410	China	G/A	58/160	4.48 (1.35, 14.87)	Participants were enrolled into the study from Eastern China, including Shanghai, Zhejiang and Jiangsu province, China
Kempinska 2012, 22690210	*VDR*	rs731236	Poland	T/C	143/306	1.61 (1.22, 2.13)	Cases were from Poland and controls were umbilical cord blood samples from healthy newborn children
Tanaka2009, 19376604	*VDR*	rs731236	Italy	T/C	139/156	0.99 (0.71, 1.37)	Patients were recruited at one center in Italy
Vogel 2002, 11786968	*VDR*	rs731236	German	T/C	74/214	0.79 (0.53, 1.17)	Cases were from Germany and an autoimmune disease-free control group consisted of Northern German white patients
Tanaka2009, 19376604	*VDR*	rs731236	Japan	T/C	195/179	1.27 (0.78, 2.04)	Patients were recruited at several referral hospitals in Japan
Fan 2005, 15683428	*VDR*	rs731236	China	T/C	58/160	0.64 (0.24, 1.74)	Participants were enrolled into the study from Eastern China, including Shanghai, Zhejiang and Jiangsu province, China
Kempinska 2012, 22690210	*VDR*	rs7975232	Poland	G /T	143/306	0.84 (0.63, 1.20)	Cases were from Poland and controls were umbilical cord blood samples from healthy newborn children
Tanaka2009, 19376604	*VDR*	rs7975232	Italy	G /T	139/156	1.02 (0.73, 1.44)	Patients were recruited at one center in Italy
Vogel 2002, 11786968	*VDR*	rs7975232	German	G /T	74/214	0.77 (0.53, 1.11)	Cases were from Germany and an autoimmune disease-free control group consisted of Northern German white patients
Tanaka2009, 19376604	*VDR*	rs7975232	Japan	G /T	195/179	1.70 (1.27, 2.27)	Patients were recruited at several referral hospitals in Japan
Fan 2005, 15683428	*VDR*	rs7975232	China	G /T	58/160	1.08 (0.69, 1.70)	Participants were enrolled into the study from Eastern China, including Shanghai, Zhejiang and Jiangsu province, China
Niro 2009, 19758199	*TNF-α*	rs361525	Italy	G/A	52/61	0.25 (0.05, 1.17)	One medical center located in San Giovanni Rotondo in the south of Italy
Niro 2009, 19758199	*TNF-α*	rs361525	Italy	G/A	55/80	0.82 (0.24, 2.89)	One medical center located in Milan in the North of Italy
Gordon 1999, 10453936	*TNF-α*	rs361525	England	G/2	91/205	1.24 (0.62, 2.49)	England Caucasian patients with PBC were recruited, and the control group were unrelated North of England Caucasians with no family history of autoimmune disease
Jones 1999, 10068101	*TNF-α*	rs361525	England	G/A	168/145	1.40 (0.63, 3.14)	Unrelated PBC patients from the North-East of England attending the Freeman Hospital PBC clinic
Kuś 2017, 28922436	*FCRL3*	rs7528684	Poland	A/G	230/421	1.01 (0.79, 1.27)	All patients were recruited in two Polish university Centres (Pomeranian Medical University, Szczecin and Medical University of Warsaw
Tanaka 2011, 21299530	*FCRL3*	rs7528684	Italy	T/C	216/180	0.84 (0.60, 1.18)	Patients were recruited at one center in Italy
Tanaka 2011, 21299530	*FCRL3*	rs7528684	Japan	T/C	232/230	0.39 (0.26, 0.58)	Patients were recruited at several referral hospitals in Japan
Tang 2016, 26842849	*AKAP11*	rs9533090	UK	/T	1840/5163	1.21 (1.11, 1.31)	Welcome Trust Case Control Consortium (WTCCC) PBC data
Tang 2016, 26842849	*AKAP11*	rs9533090	Italy	/T	453/936	1.20 (1.01, 1.42)	Italian PBC data

Tang 2016, 26842849	*AKAP11*	rs9533090	China	/T	683/1152	1.20 (1.01, 1.42)	A South China cohort.
Tang 2016, 26842849	*MAPT*	rs1864325	UK	/T	1840/5163	0.80 (0.72, 0.88)	Welcome Trust Case Control Consortium (WTCCC) PBC data
Tang 2016, 26842849	*MAPT*	rs1864325	Italy	/T	453/936	0.75 (0.61, 0.92)	Italian PBC data
Tang 2016, 26842849	*MAPT*	rs1864325	China	/T	683/1152	0.75 (0.61, 0.92)	A South China cohort.
Tang 2016, 26842849	*CLDN14*	rs170183	UK	A/G	1840/5163	0.89 (0.82, 0.97)	Welcome Trust Case Control Consortium (WTCCC) PBC data
Tang 2016, 26842849	*CLDN14*	rs170183	Italy	A/G	453/936	0.82 (0.68, 0.97)	Italian PBC data
Tang 2016, 26842849	*CLDN14*	rs170183	China	A/G	683/1152	0.81 (0.68, 0.97)	A South China cohort.
Hitomi 2022, 34864633	*FGFR1OP*	rs9459874	China,Japan	T/C	2495/4283	1.23 (1.13, 1.32)	Japan PBC-GWAS Consortium and China Jiangsu Province PBC Collaboration Group
Hitomi 2022, 34864633	*FGFR1OP*	rs9459874	Mix-Europe	T/C	8021/16489	1.08 (1.03, 1.12)	Canadian–US PBC Consortium, Italian PBC Genetics Study Group and UK-PBC Consortium.
Paziewska 2017, 28056976	*CCR6*	rs9459874	Poland	/T	443/934	1.29 (1.08, 1.53)	Polish Caucasians were recruited at nine recruitment centers
Fan 2005, 15884119	*IL-10*	-592 C/A	China	A/C	77/160	1.23 (0.83, 1.83)	Participants were enrolled into the study from Eastern China, including Shanghai, Zhejiang and Jiangsu province, China
Matsushita 2002, 12765479	*IL-10*	-592 C/A	Japan	A/C	65/71	1.22 (0.73, 2.03)	Subjects originated from Teikyo University School of Medicine (Kanagawa, Japan)
Matsushita 2002, 12765479	*IL-10*	-592 C/A	Italy	A/C	94/72	1.34 (0.82, 2.21)	Subjects originated from Molinette Hospital (Torino, Italy)
Zappala 1998, 9625317	*IL-10*	-592 C/A	England	A/C	171/141	0.85 (0.57, 1.25)	North East of England, attending the Freeman Hospital PBC Clinic
Fan 2005, 15884119	*IL-10*	-1082 G/A	China	A/G	77/160	1.75 (0.82, 3.74)	Participants were enrolled into the study from Eastern China, including Shanghai, Zhejiang and Jiangsu province, China
Matsushita 2002, 12765479	*IL-10*	-1082 G/A	Japan	A/G	65/71	0.97 (0.36, 2.59)	Subjects originated from Teikyo University School of Medicine (Kanagawa, Japan)
Matsushita 2002, 12765479	*IL-10*	-1082 G/A	Italy	A/G	94/72	1.63 (1.04, 2.56)	Subjects originated from Molinette Hospital (Torino, Italy)
Fan 2005, 15884119	*IL-10*	-819 C/T	China	C/T	77/160	0.79 (0.53, 1.19)	Participants were enrolled into the study from Eastern China, including Shanghai, Zhejiang and Jiangsu province, China
Matsushita 2002, 12765479	*IL-10*	-819 C/T	Japan	C/T	65/71	0.82 (0.49, 1.36)	Subjects originated from Teikyo University School of Medicine (Kanagawa, Japan)
Matsushita 2002, 12765479	*IL-10*	-819 C/T	Italy	C/T	94/72	0.74 (0.45, 1.22)	Subjects originated from Molinette Hospital (Torino, Italy)

† The participants were repeated.

Supplementary Table 4. Characteristics of the candidate-gene association studies on variants in Non-HLA genes and risk of primary biliary cirrhosis with datasets less than three.

Study, PMID	Gene	Variants	Country	Cases/ Controls	OR (95%CI)
Zhang 2024, 38405661	*CCR6*	rs6905911	China	1960/3316	1.17 (1.08, 1.26)
Hitomi 2022, 34864633	*CCR6*	rs6905911	Mix_Europe	2495/4283	1.26 (1.16, 1.36)
Kruk 2022, 36397154	*ABCB4*	c.711A > T	Poland	196/150	1.16 (0.78, 1.73)
Kruk 2022, 36397154	*ABCB4*	c.711A > T	Poland	260/318	1.24 (0.90, 1.70)
Hitomi 2015, 25899471	*TNFSF15*	rs4979462	Japan	1279/1091	1.57 (1.40, 1.76)
Dong 2015, 25690649	*TNFSF15*	rs4979462	China	1070/1198	1.42 (1.26, 1.62)
Joshita 2014, 24648611	*IL12A*	rs574808	Japan	395/458	0.96 (0.75, 1.23)
Tanaka 2011, 21506939	*IL12A*	rs574808	Japan	303/298	0.75 (0.55, 1.02)
Li 2016, 27175695	*IL12A*	rs485499	China	586/726	1.11 (0.88, 1.40)
Dong 2015, 25690649	*IL12A*	rs485499	China	1070/1198	1.20 (1.01, 1.44)
Tanaka 2011, 21299530	*FCRL3*	rs11264799	Japan	232/230	0.81 (0.60, 1.09)
Tanaka 2011, 21299530	*FCRL3*	rs11264799	Italy	216/180	1.28 (0.93, 1.76)
Hitomi 2022, 34864633	*CCR6*	rs968334	Mix_Europe	2495/4283	1.24 (1.14, 1.34)
Hitomi 2022, 34864633	*CCR6*	rs968334	Mix_Europe	8021/16489	1.10 (1.05, 1.15)
Baragiotta 2004, 15566517	*CCR5*	Δ32	Italy	82/92	1.50 (0.81, 2.80)
Baragiotta 2004, 15566517	*CCR5*	Δ32	UK	144/105	1.09 (0.61, 1.93)
Bittencourt 2003, 12911663	*CTLA-4*	exon 1	Brazil	50/67	0.86 (0.49, 1.50)
Agarwal 2000, 10782900	*CTLA-4*	exon 1	England	200/200	1.86 (1.40, 2.49)
Kuś 2017, 28922436	*PTPN22*	rs2476601	Poland	230/421	0.70 (0.50, 0.98)
Milkiewicz 2006, 16671954	*PTPN22*	rs2476601	Canada	160/290	0.88 (0.53, 1.46)
Fan 2005, 15683428	*VDR*	Fok I	China	58/160	1.29 (0.84, 1.98)
Vogel 2002, 11786968	*VDR*	Fok I	German	74/214	0.72 (0.49, 1.05)
Dong 2015, 25690649	*SOCS1*	rs725613	China	1070/1198	1.10 (0.96, 1.27)
Hirschfield 2012, 22257840	*CLEC16A*	rs725613	Mix_Europe	1450/2967	1.20 (1.09, 1.31)
Hirschfield 2012, 22257840	*SPIB*	rs3745516	Mix_Europe	1450/2967	1.30 (1.18, 1.43)
Paziewska 2017, 28056976	*SPIB*	rs3745516	Poland	443/934	1.45 (1.21, 1.74)
Jawed 2020, 33284381	*RUNX3*	rs7529070	China	1118/4036	1.23 (1.12, 1.36)
Jawed 2020, 33284381	*RUNX3*	rs7529070	China	1435/3205	1.13 (1.02, 1.24)
Umemura 2017, 28703133	*SH2B3*	rs11065904	Japan	327/325	0.89 (0.72, 1.11)
Umemura 2017, 28703133	*SH2B3*	rs739496	Japan	327/325	0.98 (0.73, 1.31)
Greverath 2020, 31747477	*CHRM3*	rs11578320	German	306/240	1.15 (0.78, 1.71)
Greverath 2020, 31747477	*CHRM3*	rs6690809	German	306/240	1.14 (0.90, 1.45)
Greverath 2020, 31747477	*CHRM3*	rs6429157	German	306/240	1.10 (0.86, 1.40)
Greverath 2020, 31747477	*CHRM3*	rs7548522	German	306/240	1.46 (0.91, 2.32)
Greverath 2020, 31747477	*CHRM3*	rs4620530	German	306/240	1.46 (1.15, 1.86)
Jiang 2023, 37348755	*Jak1*	rs4915675	China	2018/3142	1.20 (1.10, 1.30)
Jiang 2023, 37348755	*Jak1*	rs7531799	China	2018/3142	1.19 (1.10, 1.29)
Wu 2024, 37713154	*GTF2I*	rs117026326	China	466/694	1.56 (1.27, 1.96)
Wu 2024, 37713154	*GTF2I*	rs73366469	China	466/694	1.49(1.22, 1.85)
Kruk 2024, 38036008	*ARID3A*	rs2238574	Poland	563/319	1.54 (1.15, 2.05)

Zhang 2024, 38405661	*CCR6*	rs4710181	China	1960/3316	1.14 (1.06, 1.23)
Zhang 2024, 38405661	*CCR6*	rs12529876	China	1960/3316	1.18 (1.10, 1.27)
Dong 2015, 25690649	*TNFRSF1A*	rs1800693	China	1070/1198	1.16 (0.98, 1.38)
Tanaka 2011, 21506939	*GSDMB*	rs2305480	Japan	303/298	1.38 (1.06, 1.80)
Dong 2015, 25690649	*CD80*	rs2293370	China	1070/1198	1.44 (1.27, 1.63)
Li 2016, 27175695	*IL12A*	rs6441286	China	586/726	1.55 (1.25, 1.92)
Paziewska 2017, 28056976	*SMKR1*	rs10488631		443/934	1.43 (1.14, 1.80)
Hitomi 2017, 28588209	*IKZF3*	rs907092	Japan	1389/2578	1.42 (1.27, 1.58)
Stallhofer 2011, 21304239	*4q27*	rs6822844	German	124/1487	0.92 (0.64, 1.32)
Gaj 2008, 18715515	*NOD2/CARD15*	rs2066844	Poland	144/139	2.46 (0.76, 7.95)
Joshita 2014, 24648611	*STAT4*	rs10168266	Japan	395/458	1.31 (1.07, 1.61)
Dong 2015, 25690649	*POU2AF1*	rs4938534	China	1070/1198	1.17 (1.04, 1.31)
Dong 2015, 25690649	*NF-kB1*	rs7665090	China	1070/1198	1.26 (1.13, 1.42)
Wasik 2017, 28299343	*IL12RB2*	rs6679356	Poland	306/258	1.60 (1.20, 2.10)
Hiraide 2005, 15929764	*Fas*	rs1800682	Japan	98/132	0.84 (0.57, 1.21)
Umemura 2016, 27406031	*PTPN22*	rs1217412	Japan	262/322	1.01 (0.80, 1.27)
Umemura 2016, 27406031	*PTPN22*	rs2488457	Japan	262/322	0.84 (0.67, 1.07)
Gaj 2008, 18715515	*OCTN1*	rs1050152	Poland	144/139	1.06 (0.76, 1.48)
Gaj 2008, 18715515	*IL23R*	rs11209026	Poland	144/139	1.68 (0.65, 4.34)
Gaj 2008, 18715515	*DLG5*	rs1248696	Poland	144/139	0.89 (0.51, 1.55)
Stallhofer 2011, 21304239	*4q27*	rs13119723	German	124/1487	1.03 (0.73, 1.45)
Gaj 2008, 18715515	*NOD2/CARD15*	rs2066845	Poland	144/139	0.48 (0.14, 1.60)
Gaj 2008, 18715515	*SLC22A5*	rs2631367	Poland	144/139	0.95 (0.68, 1.31)
Matsushita 2001, 11712863	*MBL*	HYPA/HYPA	Japan	65/218	2.50 (1.34, 4.67)
Matsushita 2001, 11712863	*MBL*	HYPA/LYPA	Japan	65/218	2.51 (0.92, 6.88)
Matsushita 2001, 11712863	*MBL*	HYPA/LYQA	Japan	65/218	0.59 (0.24, 1.48)
Matsushita 2001, 11712863	*MBL*	LYPA/LYPA	Japan	65/218	3.39 (0.21, 54.97)
Matsushita 2001, 11712863	*MBL*	LYPA/LYQA	Japan	65/218	0.47 (0.06, 3.90)
Matsushita 2001, 11712863	*MBL*	LYQA/LYQA	Japan	65/218	0.55 (0.07, 4.67)
Matsushita 2001, 11712863	*MBL*	HYPA/LXPA	Japan	65/218	0.51 (0.17, 1.51)
Matsushita 2001, 11712863	*MBL*	LYPA/LXPA	Japan	65/218	1.35 (0.26, 7.14)
Matsushita 2001, 11712863	*MBL*	HYPA/LYPB	Japan	65/218	0.47 (0.21, 1.05)
Matsushita 2001, 11712863	*MBL*	LYPA/LYPB	Japan	65/218	2.06 (0.48, 8.87)
Matsushita 2001, 11712863	*MBL*	LYQA/LYPB	Japan	65/218	1.28 (0.48, 3.43)
Matsushita 2001, 11712863	*MBL*	LYPB/LXPA	Japan	65/218	1.01 (0.27, 3.77)
Matsushita 2001, 11712863	*MBL*	LYPB/LYPB	Japan	65/218	0.47 (0.06, 3.90)
Selmi 2003, 12974901	*eNOS*	rs1799983	Italy	109/242	0.90 (0.65, 1.26)
Selmi 2003, 12974901	*eNOS*	rs2070744	Italy	109/242	1.30 (0.94, 1.79)
Kimura 2005, 15690482	*CYP2D6*	G1934A	Italy	169/225	1.13 (0.76, 1.67)
Kimura 2005, 15690482	*CYP2E1*	c1/c2	Italy	169/225	1.02 (0.44, 2.34)
Kimura 2005, 15690482	*MDR1*	rs1045642	Italy	169/225	0.90 (0.68, 1.19)
Kimura 2005, 15690482	*PXR*	C-25385 T	Italy	169/225	0.84 (0.63, 1.12)
Kimura 2005, 15690482	*PXR*	A7635G	Italy	169/225	1.06 (0.72, 1.57)

Kimura 2005, 15690482	*PXR*	C8055T	Italy	169/225	0.89 (0.55, 1.45)
Kikuchi2007, 17911416	*TGF-1*	rs1800469	Japan	65/71	1.13 (0.70, 1.83)
Juran 2009, 19491853	*SLC4A2*	rs33966546	US	409/300	1.05 (0.83, 1.34)
Juran 2009, 19491853	*SLC4A2*	rs2303929	US	409/300	1.00 (0.78, 1.28)
Juran 2009, 19491853	*SLC4A2*	rs2303930	US	409/300	0.88 (0.70, 1.09)
Juran 2009, 19491853	*SLC4A2*	rs3793336	US	409/300	1.00 (0.76, 1.32)
Juran 2009, 19491853	*SLC4A2*	rs35576067	US	409/300	1.02 (0.55, 1.89)
Juran 2009, 19491853	*SLC4A2*	rs12703112	US	409/300	1.14 (0.87, 1.48)
Juran 2009, 19491853	*SLC4A2*	rs13247141	US	409/300	1.07 (0.82, 1.40)
Juran 2009, 19491853	*SLC4A2*	rs2303933	US	409/300	1.09 (0.88, 1.35)
Juran 2009, 19491853	*SLC4A2*	rs2303934	US	409/300	1.01 (0.59, 1.73)
Juran 2009, 19491853	*SLC4A2*	rs2303937	US	409/300	1.09 (0.88, 1.34)
Juran 2009, 19491853	*SLC4A2*	rs11765015	US	409/300	1.06 (0.83, 1.35)
Juran 2009, 19491853	*SLC4A2*	rs11766855	US	409/300	1.07 (0.81, 1.41)
Joshita 2010, 20153395	*A2BP1*	rs17139207	Japan	126/95	1.38 (0.70, 2.74)
Joshita 2010, 20153395	*A2BP1*	rs12926282	Japan	126/95	1.35 (0.85, 2.16)
Joshita 2010, 20153395	*A2BP1*	rs17139244	Japan	126/95	0.64 (0.43, 0.94)
Joshita 2010, 20153395	*A2BP1*	rs6500742	Japan	126/95	0.64 (0.44, 0.94)
Joshita 2010, 20153395	*A2BP1*	rs4146812	Japan	126/95	1.48 (0.94, 2.32)
Joshita 2010, 20153395	*A2BP1*	rs4124065	Japan	126/95	1.01 (0.65, 1.55)
Joshita 2010, 20153395	*A2BP1*	rs889699	Japan	126/95	0.96 (0.65, 1.43)
Inamine 2011, 21116829	*ITGAV*	rs3911238	Japan	309/293	0.99 (0.77, 1.28)
Inamine 2011, 21116829	*ITGAV*	rs12611439	Japan	309/293	1.16 (0.86, 1.56)
Inamine 2011, 21116829	*ITGAV*	rs1992898	Japan	309/293	0.97 (0.69, 1.36)
Inamine 2011, 21116829	*ITGAV*	rs10174098	Japan	309/293	0.90 (0.68, 1.19)
Inamine 2011, 21116829	*ITGAV*	rs4667107	Japan	309/293	1.03 (0.82, 1.29)
Inamine 2011, 21116829	*ITGAV*	rs3768785	Japan	309/293	1.12 (0.87, 1.46)
Inamine 2011, 21116829	*ITGAV*	rs1448427	Japan	309/293	1.13 (0.85, 1.49)
Hirschfield 2012, 22257840	*CLEC16A*	rs16957895	Mix_Europe	1450/2967	1.24 (1.12, 1.37)
Hirschfield 2012, 22257840	*CLEC16A*	rs16957976	Mix_Europe	1450/2967	1.39 (1.21, 1.61)
Hirschfield 2012, 22257840	*CLEC16A*	rs876457	Mix_Europe	1450/2967	1.43 (1.21, 1.69)
Hirschfield 2012, 22257840	*CLEC16A*	rs58102322	Mix_Europe	1450/2967	1.46 (1.26, 1.69)
Hirschfield 2012, 22257840	*CLEC16A*	rs2041670	Mix_Europe	1450/2967	1.24 (1.13, 1.37)
Hirschfield 2012, 22257840	*CLEC16A*	rs12708716	Mix_Europe	1450/2967	1.20 (1.10, 1.32)
Hirschfield 2012, 22257840	*CLEC16A*	rs12924729	Mix_Europe	1450/2967	1.25 (1.13, 1.38)
Hirschfield 2012, 22257840	*CLEC16A*	rs12927355	Mix_Europe	1450/2967	1.25 (1.14, 1.38)
Hirschfield 2012, 22257840	*CLEC16A*	rs12927046	Mix_Europe	1450/2967	1.34 (1.14, 1.57)
Hirschfield 2012, 22257840	*CLEC16A*	rs11074956	Mix_Europe	1450/2967	1.19 (1.09, 1.31)
Hirschfield 2012, 22257840	*SOCS1b*	rs1111186	Mix_Europe	1450/2967	1.27 (1.15, 1.40)
Hirschfield 2012, 22257840	*SOCS1*	rs4780355	Mix_Europe	1450/2967	1.25 (1.14, 1.38)
Hirschfield 2012, 22257840	*SOCS1*	rs33989964	Mix_Europe	1450/2967	1.30 (1.17, 1.44)
Hirschfield 2012, 22257840	*SOCS1*	rs243330	Mix_Europe	1450/2967	1.19 (1.09, 1.30)

Hirschfield 2012, 22257840	*SOCS1*	rs243329	Mix_Europe	1450/2967	1.22 (1.11, 1.33)
Hirschfield 2012, 22257840	*SOCS1*	rs243327	Mix_Europe	1450/2967	1.23 (1.12, 1.34)
Hirschfield 2012, 22257840	*SOCS1*	rs243325	Mix_Europe	1450/2967	1.33 (1.21, 1.47)
Hirschfield 2012, 22257840	*SOCS1*	rs243324	Mix_Europe	1450/2967	1.22 (1.12, 1.34)
Hirschfield 2012, 22257840	*TNP2*	rs243323	Mix_Europe	1450/2967	1.30 (1.18, 1.44)
Hirschfield 2012, 22257840	*TNP2*	rs11640138	Mix_Europe	1450/2967	1.19 (1.09, 1.30)
Hirschfield 2012, 22257840	*TNP2*	rs416603	Mix_Europe	1450/2967	1.18 (1.08, 1.29)
Hirschfield 2012, 22257840	*PRM3*	rs367569	Mix_Europe	1450/2967	1.34 (1.21, 1.48)
Hirschfield 2012, 22257840	*PRM1*	rs181694	Mix_Europe	1450/2967	1.24 (1.10, 1.40)
Hirschfield 2012, 22257840	*CLEC16A*	rs12928822	Mix_Europe	1450/2967	1.34 (1.17, 1.52)
Hirschfield 2012, 22257840	*CLEC16A*	rs7203055	Mix_Europe	1450/2967	1.22 (1.11, 1.35)
Hirschfield 2012, 22257840	*SPIB*	rs62116060	Mix_Europe	1450/2967	1.30 (1.18, 1.44)
Hirschfield 2012, 22257840	*SPIB*	rs62116061	Mix_Europe	1450/2967	1.30 (1.18, 1.44)
Hirschfield 2012, 22257840	*SPIB*	rs11546996	Mix_Europe	1450/2967	1.30 (1.18, 1.44)
Hirschfield 2012, 22257840	*SPIB*	rs34944112	Mix_Europe	1450/2967	1.49 (1.31, 1.70)
Hirschfield 2012, 22257840	*SPIB*	rs8108811	Mix_Europe	1450/2967	1.30 (1.18, 1.43)
Hirschfield 2012, 22257840	*SPIB*	rs34795041	Mix_Europe	1450/2967	1.48 (1.30, 1.70)
Hirschfield 2012, 22257840	*SPIB*	rs56235776	Mix_Europe	1450/2967	1.18 (1.08, 1.30)
Hirschfield 2012, 22257840	*SPIBb*	rs1137895	Mix_Europe	1450/2967	1.31 (1.19, 1.46)
Hirschfield 2012, 22257840	*SPIBb*	rs3810277	Mix_Europe	1450/2967	1.41 (1.25, 1.58)
Hirschfield 2012, 22257840	*MYBPC2*	rs3765069	Mix_Europe	1450/2967	1.22 (1.11, 1.33)
Kempinska 2012, 23125866	*TRAF1*	rs3761847	Poland	179/300	1.04 (0.79, 1.35)
Kempinska 2012, 23125866	*TRAF1*	rs2900180	Poland	179/300	1.05 (0.79, 1.39)
Morita 2013, 23142582	*TLR4*	rs10759930	Japan	261/359	1.07 (0.85, 1.36)
Morita 2013, 23142582	*TLR4*	rs2149356	Japan	261/359	0.96 (0.76, 1.22)
Morita 2013, 23142582	*TLR4*	rs11536889	Japan	261/359	0.86 (0.66, 1.12)
Morita 2013, 23142582	*TLR4*	rs7037117	Japan	261/359	1.02 (0.76, 1.36)
Morita 2013, 23142582	*TLR4*	rs7045953	Japan	261/359	1.17 (0.78, 1.75)
Ohishi 2014, 23612856	*OCT-1/SLC22a1*	rs683369	Japan	275/194	0.60 (0.40, 0.88)
Ohishi 2014, 23612856	*OCT-1/SLC22a1*	rs2282143	Japan	275/194	1.13 (0.79, 1.58)
Ohishi 2014, 23612856	*OCT-1/SLC22a1*	rs622342	Japan	275/194	0.82 (0.57, 1.16)
Ohishi 2014, 23612856	*OCT-1/SLC22a1*	rs1443844	Japan	275/194	1.02 (0.76, 1.35)
Kong 2013, 23622253	*TNFSF4*	rs2205960	China	221/393	1.30 (1.01, 1.68)
Kong 2013, 23622253	*TNFSF4*	rs16845607	China	221/393	0.87 (0.53, 1.45)
Kong 2013, 23622253	*TNFSF4*	rs1234313	China	221/393	0.99 (0.80, 1.29)
Kong 2013, 23622253	*TNFSF4*	rs3861950	China	221/393	0.96 (0.64, 1.45)
Chen 2014, 25392597	*BSEP*	rs52304393	China	134/314	0.81 (0.57, 1.17)
Chen 2014, 25392597	*BSEP*	rs473351	China	134/314	2.00 (1.27, 3.14)
Chen 2014, 25392597	*BSEP*	rs860510	China	134/314	1.16 (0.86, 1.56)
Chen 2014, 25392597	*BSEP*	rs2287618	China	134/314	1.03 (0.74, 1.45)
Aiba 2015, 26084578	*C1orf94*	rs4652997	Japan	1279/1015	0.98 (0.88, 1.11)

Aiba 2015, 26084578	*RUNX3*	rs7551188	Japan	1279/1015	1.03 (0.92, 1.16)
Aiba 2015, 26084578	-	rs11894081	Japan	1279/1015	0.89 (0.80, 1.01)
Aiba 2015, 26084578	*TBC1D1*	rs1487630	Japan	1279/1015	1.09 (0.95, 1.25)
Aiba 2015, 26084578	*JAK2*	rs2274471	Japan	1279/1015	1.00 (0.86, 1.17)
Aiba 2015, 26084578	*TNFSF15*	rs6478106	Japan	1279/1015	1.46 (1.30, 1.64)
Aiba 2015, 26084578	*CCDC6*	rs7094419	Japan	1279/1015	0.97 (0.85, 1.12)
Aiba 2015, 26084578	*ELF1*	rs7329174	Japan	1279/1015	1.04 (0.91, 1.18)
Aiba 2015, 26084578	*STAT3*	rs9891119	Japan	1279/1015	0.98 (0.87, 1.11)
Aiba 2015, 26084578	*VAMP3*	rs2797685	Japan	1279/1015	0.94 (0.83, 1.05)
Aiba 2015, 26084578	*CD244*	rs4656940	Japan	1279/1015	1.04 (0.92, 1.17)
Aiba 2015, 26084578	*TNFSF18*	rs7517810	Japan	1279/1015	1.01 (0.82, 1.24)
Aiba 2015, 26084578	*REL*	rs10181042	Japan	1279/1015	1.07 (0.82, 1.40)
Aiba 2015, 26084578	*GCKR*	rs780093	Japan	1279/1015	1.07 (0.95, 1.20)
Aiba 2015, 26084578	*PLCL1*	rs6738825	Japan	1279/1015	0.97 (0.85, 1.10)
Aiba 2015, 26084578	-	rs7702331	Japan	1279/1015	0.93 (0.80, 1.08)
Aiba 2015, 26084578	*IL12B*	rs6556412	Japan	1279/1015	0.85 (0.76, 0.96)
Aiba 2015, 26084578	-	rs17309827	Japan	1279/1015	0.99 (0.88, 1.11)
Aiba 2015, 26084578	*CCR6*	rs415890	Japan	1279/1015	1.12 (0.99, 1.25)
Aiba 2015, 26084578	*CREM*	rs12242110	Japan	1279/1015	1.06 (0.93, 1.21)
Aiba 2015, 26084578	*UBE2D1*	rs1819658	Japan	1279/1015	0.94 (0.83, 1.06)
Aiba 2015, 26084578	*ZNF365*	rs10761659	Japan	1279/1015	1.00 (0.88, 1.14)
Aiba 2015, 26084578	*ZMIZ1*	rs1250550	Japan	1279/1015	0.95 (0.85, 1.07)
Aiba 2015, 26084578	*NKX2-3*	rs4409764	Japan	1279/1015	0.91 (0.81, 1.03)
Aiba 2015, 26084578	*PRDX5*	rs6494739	Japan	1279/1015	1.14 (0.99, 1.32)
Aiba 2015, 26084578	*C13orf31*	rs3764147	Japan	1279/1015	1.02 (0.90, 1.16)
Aiba 2015, 26084578	*GPR65*	rs8005161	Japan	1279/1015	0.92 (0.79, 1.07)
Aiba 2015, 26084578	*IL27*	rs151181	Japan	1279/1015	0.86 (0.72, 1.03)
Aiba 2015, 26084578	*TNFRSF6B*	rs4809330	Japan	1279/1015	0.93 (0.83, 1.05)
Aiba 2015, 26084578	-	rs1736020	Japan	1279/1015	0.97 (0.83, 1.05)
Aiba 2015, 26084578	*ICOSLG*	rs2838519	Japan	1279/1015	1.14 (1.01, 1.28)
Aiba 2015, 26084578	*YDJC*	rs181359	Japan	1279/1015	0.93 (0.83, 1.05)
Aiba 2015, 26084578	*MTMR3*	rs713875	Japan	1279/1015	0.95 (0.83, 1.09)
Li 2016, 27047549	*HELZ2*	rs3810481	China	586/726	0.80 (0.65, 0.99)
Li 2016, 27047549	*HELZ2*	rs3810483	China	586/726	0.82 (0.60, 1.13)
Li 2016, 27047549	*HELZ2*	rs79267778	China	586/726	4.2 (1.67, 10.58)
Li 2016, 27047549	*HELZ2*	rs3810486	China	586/726	0.82 (1.42, 2.35)
Li 2016, 27047549	*HELZ2*	rs6089924	China	586/726	0.38 (0.10, 1.43)
Paziewska 2017, 28056976	*IL12RB2*	rs10489626	Poland	443/934	1.59 (1.28, 1.97)
Paziewska 2017, 28056976	*IL24, CTSE*	rs3024505	Poland	443/934	1.25 (1.00, 1.56)
Paziewska 2017, 28056976	*ABCB11*	rs2287619	Poland	443/934	1.50 (1.11, 2.02)
Paziewska 2017, 28056976	*MIR3120*	rs12118836	Poland	443/934	1.37 (1.09, 1.73)
Paziewska 2017, 28056976	*PVRL3-AS1*	rs28413019	Poland	443/934	1.25 (1.03, 1.51)

Paziewska 2017, 28056976	*MIR1284*	rs1491590	Poland	443/934	1.34 (1.11, 1.61)
Paziewska 2017, 28056976	*TBC1D19*	rs13126571	Poland	443/934	1.24 (1.02, 1.51)
Paziewska 2017, 28056976	*RBM47*	rs3114381	Poland	443/934	1.28 (1.06, 1.55)
Paziewska 2017, 28056976	*TRAM1L1*	rs979961	Poland	443/934	1.39 (1.10, 1.76)
Paziewska 2017, 28056976	*LOC100294145*	rs9268979	Poland	443/934	0.68 (0.57, 0.80)
Paziewska 2017, 28056976	*MIR1275*	rs3128927	Poland	443/934	1.50 (1.24, 1.81)
Paziewska 2017, 28056976	*CCR6*	rs4710185	Poland	443/934	1.23 (1.03, 1.46)
Paziewska 2017, 28056976	*CCR6*	rs975822	Poland	443/934	1.29 (1.08, 1.54)
Paziewska 2017, 28056976	*BBS9*	rs965571	Poland	443/934	1.43 (1.16, 1.76)
Paziewska 2017, 28056976	*POLR2G*	rs35730843	Poland	443/934	0.39 (0.26, 0.60)
Paziewska 2017, 28056976	*MIR4299*	rs12786216	Poland	443/934	0.77 (0.64, 0.93)
Paziewska 2017, 28056976	*LOC100131626*	rs11217040	Poland	443/934	0.66 (0.54, 0.81)
Paziewska 2017, 28056976	*SNX19*	rs73022813	Poland	443/934	1.24 (1.01, 1.53)
Paziewska 2017, 28056976	*KLRF2*	rs7975557	Poland	443/934	1.54 (1.02, 2.33)
Paziewska 2017, 28056976	*MIPOL1*	rs10083358	Poland	443/934	1.34 (1.11, 1.62)
Paziewska 2017, 28056976	*ERI2, IQCK*	rs8055224	Poland	443/934	0.54 (0.34, 0.85)
Paziewska 2017, 28056976	*CDH13*	rs8049648	Poland	443/934	1.68 (1.21, 2.32)
Paziewska 2017, 28056976	*KRTAP21-3*	rs7279062	Poland	443/934	0.80 (0.67, 0.95)
Paziewska 2017, 28056976		rs4927257	Poland	443/934	0.83 (0.70, 0.99)
Paziewska 2017, 28056976	*ALLC*	rs1965732	Poland	443/934	1.24 (1.02, 1.50)
Paziewska 2017, 28056976	*WWC1*	rs9686714	Poland	443/934	0.68 (0.46, 1.00)
Paziewska 2017, 28056976	*C4B_2*	rs7775228	Poland	443/934	1.84 (1.50, 2.26)
Paziewska 2017, 28056976	*COL11A2*	rs1799908	Poland	443/934	1.21 (1.02, 1.44)
Paziewska 2017, 28056976	*C4B_2*	rs7454108	Poland	443/934	1.43 (1.09, 1.87)
Paziewska 2017, 28056976	*TRAF2*	rs10119096	Poland	443/934	0.70 (0.57, 0.86)
Paziewska 2017, 28056976	*CACUL1*	rs17098094	Poland	443/934	0.81 (0.68, 0.96)
Umemura 2017, 28703133	*SH2B3*	rs2238154	Japan	327/325	1.00 (0.75, 1.34)
Hitomi 2019, 30528300	*NFKB1*	rs230534	Japan	1389/2578	1.37 (1.23, 1.53)
Hitomi 2019, 30528300	*NFKB1*	rs3518502	Japan	1389/2578	1.37 (1.23, 1.53)
Hitomi 2019, 30528300	*MANBA*	rs2978641	Japan	1389/2578	1.33 (1.20, 1.48)
Hitomi 2019, 30528300	*MANBA*	rs227277	Japan	1389/2578	1.34 (1.20, 1.49)
Hitomi 2019, 30528300	*NFKB1*	rs230531	Japan	1389/2578	1.34 (1.20, 1.49)
Hitomi 2019, 30528300	*NFKB1*	rs1109778	Japan	1389/2578	1.34 (1.20, 1.49)
Hitomi 2019, 30528300	*NFKB1*	rs1419361	Japan	1389/2578	1.34 (1.20, 1.49)
Hitomi 2019, 30528300	*MANBA*	rs228613	Japan	1389/2578	1.33 (1.20, 1.48)
Hitomi 2019, 30528300	*MANBA*	rs228612	Japan	1389/2578	1.33 (1.20, 1.48)
Hitomi 2019, 30528300	*NFKB1*	rs6232853	Japan	1389/2578	1.34 (1.20, 1.49)
Hitomi 2019, 30528300	*NFKB1*	rs2272676	Japan	1389/2578	1.34 (1.20, 1.50)
Hitomi 2019, 30528300	*MANBA*	rs2866413	Japan	1389/2578	1.32 (1.19, 1.47)
Hitomi 2019, 30528300	*MANBA*	rs227368	Japan	1389/2578	1.33 (1.19, 1.48)
Hitomi 2019, 30528300	*NFKB1*	rs230504	Japan	1389/2578	1.33 (1.19, 1.48)

Hitomi 2019, 30528300	*NFKB1*	rs3774959	Japan	1389/2578	1.32 (1.19, 1.47)
Hitomi 2019, 30528300	*NFKB1*	rs1703285	Japan	1389/2578	1.32 (1.19, 1.47)
Hitomi 2019, 30528300	*NFKB1*	rs4648055	Japan	1389/2578	1.32 (1.19, 1.47)
Hitomi 2019, 30528300	*MANBA*	rs227361	Japan	1389/2578	1.32 (1.19, 1.47)
Xu 2021, 31873148	*ETS-1*	rs4937333	China	151/398	1.44 (1.10, 1.88)
Xu 2021, 31873148	*ETS-1*	rs11221332	China	151/398	0.90 (0.49, 1.64)
Xu 2021, 31873148	*ETS-1*	rs73013527	China	151/398	0.86 (0.65, 1.13)
Hitomi 2021, 33633225	*POU2AF1*	rs12362038	Japan	1381/1070	1.35 (1.22, 1.50)
Hitomi 2021, 33633225	*POU2AF1*	rs10891259	Japan	1381/1070	1.35 (1.22, 1.50)
Hitomi 2021, 33633225	*POU2AF1*	rs4938541	Japan	1381/1070	1.34 (1.21, 1.49)
Hitomi 2021, 33633225	*POU2AF1*	rs7952176	Japan	1381/1070	1.34 (1.21, 1.49)
Hitomi 2021, 33633225	*POU2AF1*	rs1944926	Japan	1381/1070	1.34 (1.21, 1.49)
Hitomi 2021, 33633225	*POU2AF1*	rs1123066	Japan	1381/1070	1.33 (1.20, 1.47)
Hitomi 2021, 33633225	*POU2AF1*	rs4936432	Japan	1381/1070	1.33 (1.20, 1.48)
Hitomi 2021, 33633225	*POU2AF1*	rs7952497	Japan	1381/1070	1.33 (1.20, 1.48)
Hitomi 2021, 33633225	*POU2AF1*	rs1944927	Japan	1381/1070	1.32 (1.19, 1.47)
Hitomi 2021, 33633225	*POU2AF1*	rs10891261	Japan	1381/1070	1.31 (1.18, 1.46)
Hitomi 2021, 33633225	*POU2AF1*	rs6589227	Japan	1381/1070	1.30 (1.17, 1.45)
Hitomi 2021, 33633225	*POU2AF1*	rs11213871	Japan	1381/1070	1.29 (1.16, 1.43)
Hitomi 2021, 33633225	*POU2AF1*	rs1806294	Japan	1381/1070	0.78 (0.70, 0.86)
Hitomi 2021, 33633225	*POU2AF1*	rs6589226	Japan	1381/1070	1.30 (1.17, 1.45)
Hitomi 2021, 33633225	*POU2AF1*	rs7947229	Japan	1381/1070	1.29 (1.16, 1.43)
Hitomi 2021, 33633225	*POU2AF1*	rs4393359	Japan	1381/1070	1.30 (1.17, 1.45)
Hitomi 2021, 33633225	*POU2AF1*	rs4489781	Japan	1381/1070	1.30 (1.17, 1.45)
Hitomi 2021, 33633225	*POU2AF1*	rs6589224	Japan	1381/1070	1.29 (1.16, 1.43)
Hitomi 2021, 33633225	*POU2AF1*	rs12799202	Japan	1381/1070	1.29 (1.16, 1.43)
Hitomi 2021, 33633225	*POU2AF1*	rs12799471	Japan	1381/1070	1.29 (1.16, 1.43)
Hitomi 2021, 33633225	*POU2AF1*	rs4622303	Japan	1381/1070	1.29 (1.16, 1.43)
Hitomi 2021, 33633225	*POU2AF1*	rs4938508	Japan	1381/1070	1.29 (1.16, 1.44)
Hitomi 2021, 33633225	*POU2AF1*	rs4938510	Japan	1381/1070	1.29 (1.16, 1.44)
Hitomi 2021, 33633225	*POU2AF1*	rs10891264	Japan	1381/1070	0.78 (0.70, 0.87)
Hitomi 2021, 33633225	*POU2AF1*	rs34563638	Japan	1381/1070	1.29 (1.16, 1.44)
Hitomi 2021, 33633225	*POU2AF1*	rs4245182	Japan	1381/1070	1.29 (1.16, 1.44)
Hitomi 2021, 33633225	*POU2AF1*	rs1944918	Japan	1381/1070	0.78 (0.70, 0.87)
Hitomi 2021, 33633225	*POU2AF1*	rs4245183	Japan	1381/1070	1.29 (1.16, 1.44)
Hitomi 2021, 33633225	*POU2AF1*	rs12293898	Japan	1381/1070	1.29 (1.16, 1.44)
Hitomi 2021, 33633225	*POU2AF1*	rs7947717	Japan	1381/1070	1.29 (1.16, 1.44)
Hitomi 2021, 33633225	*POU2AF1*	rs4356268	Japan	1381/1070	1.29 (1.16, 1.44)
Hitomi 2021, 33633225	*POU2AF1*	rs12800418	Japan	1381/1070	1.29 (1.16, 1.44)
Hitomi 2021, 33633225	*POU2AF1*	rs7116862	Japan	1381/1070	1.28 (1.15, 1.42)
Hitomi 2021, 33633225	*POU2AF1*	rs6589225	Japan	1381/1070	1.29 (1.16, 1.44)
Hitomi 2021, 33633225	*POU2AF1*	rs4529910	Japan	1381/1070	1.28 (1.15, 1.42)

Hitomi 2021, 33633225	*POU2AF1*	rs3802843	Japan	1381/1070	1.28 (1.15, 1.42)
Hitomi 2021, 33633225	*POU2AF1*	rs7946785	Japan	1381/1070	1.29 (1.16, 1.44)
Hitomi 2021, 33633225	*POU2AF1*	rs35646619	Japan	1381/1070	1.28 (1.15, 1.42)
Li 2023, 36977669	*ARID3A*	rs2238571	China	1931/7852	0.77 (0.71, 0.84)

Supplementary Table 4. Characteristics of the the candidate-gene association studies on variants in HLA genes and risk of primary biliary cirrhosis in the meta-analysis.

Study, PMID	Ethnicity	Country / Region	Variant	Cases/ Controls	OR (95%CI)	Participants
Khor 2023, 37325616	Asian	Japan	A*330301	2328/1653	0.43 (0.35, 0.53)	Japan PBC-GWAS Consortium
Umemura 2012, 21953406	Asian	Japan	A*3303	229/523	0.43 (0.25, 0.74)	The Shinshu PBC Study Group between January 2005 and September 2010
Yasunami 2017, 28894202	Asian	Japan	A*3303	1200/1196	0.40 (0.30, 0.52)	the National Hospital Organization Study Group for Liver Disease in Japan (NHOSLJ)
Invernizzi 2003, 12663229	Caucasian	Italy	B*08	186/558	0.63 (0.26, 1.51)	Patients attending the centre in Milan (Ospedale San Paolo) between June 1974 and December 1998
Neri 2003, 14567462	Caucasian	Italy	B*08	64/183	0.72 (0.32, 1.59)	Six Northern Italian centres
Ercilla 2008, 12731577	Caucasian	Spain	B*08	21/74	4.31 (1.11, 16.7)	Unrelated Caucasian patients from Spain
Khor 2023, 37325616	Asian	Japan	B*440301	2328/1653	0.34 (0.27, 0.42)	Japan PBC-GWAS Consortium
Umemura 2012, 21953406	Asian	Japan	B*4403	229/523	0.34 (0.18, 0.65)	The Shinshu PBC Study Group between January 2005 and September 2010
Yasunami 2017, 28894202	Asian	Japan	B*4403	1200/1196	0.31 (0.23, 0.42)	the National Hospital Organization Study Group for Liver Disease in Japan (NHOSLJ)
Khor 2023, 37325616	Asian	Japan	DPB1*020102	2328/1653	0.70 (0.63, 0.79)	Japan PBC-GWAS Consortium
Yasunami 2017, 28894202	Asian	Japan	DPB1*0201	1200/1196	0.67 (0.57, 0.80)	the National Hospital Organization Study Group for Liver Disease in Japan (NHOSLJ)
Underhill 1995, 7705806	Caucasian	UK	DPB1*0201	82/103	1.02 (0.50, 2.09)	White British PSC patients From the Institute of Liver Studies, King's College Hospital and King's College School of Medicine and Dentistry, England and white Europeans was drawn from laboratory and clinical staff
Khor 2023, 37325616	Asian	Japan	DPB1*040101	2328/1653	0.26 (0.19, 0.35)	Japan PBC-GWAS Consortium
Yasunami 2017, 28894202	Asian	Japan	DPB1*0401	1200/1196	0.24 (0.17, 0.36)	the National Hospital Organization Study Group for Liver Disease in Japan (NHOSLJ)
Underhill 1995, 7705806	Caucasian	UK	DPB1*0401	82/103	1.27 (0.71, 2.27)	White British PSC patients From the Institute of Liver Studies, King's College Hospital and King's College School of Medicine and Dentistry, England and white Europeans was drawn from laboratory and clinical staff
Khor 2023, 37325616	Asian	Japan	DPB1*050101	2328/1653	1.41 (1.28, 1.54)	Japan PBC-GWAS Consortium
Yasunami 2017, 28894202	Asian	Japan	DPB1*0501	1200/1196	1.36 (1.15, 1.60)	the National Hospital Organization Study Group for Liver Disease in Japan (NHOSLJ)
Underhill 1995, 7705806	Caucasian	UK	DPB1*0501	82/103	0.89 (0.27, 2.92)	White British PSC patients From the Institute of Liver Studies, King's College Hospital and King's College School of Medicine and Dentistry, England and white Europeans was drawn from laboratory and clinical staff
Khor 2023, 37325616	Asian	Japan	DQA1*0102:01	2328/1653	0.38 (0.32, 0.45)	Japan PBC-GWAS Consortium
Yasunami 2017, 28894202	Asian	Japan	DQA1*0102	1200/1196	0.47 (0.37, 0.59)	the National Hospital Organization Study Group for Liver Disease in Japan (NHOSLJ)
Mullarkey 2005, 15713222	Caucasian	USA	DQA1*0102	72/381	0.33 (0.15, 0.66)	Patients with PBC were recruited from the practices of gastroenterologists primarily from Washington State and the Seattle area. Control healthy women were recruited from the Seattle area
Donaldson 2006, 16941709	Caucasian	UK	DQA1*0102	412/236	1.05 (0.69, 1.61)	Patients from 2 major UK centers, all patients and controls were of northern European ancestry
Donaldson 2001, 11171832	Caucasian	UK	DQA1*0102	164/102	1.15 (0.66, 2.00)	All were of northern European ancestry and resident within the Newcastle area
Donaldson 2006, 16941709	Caucasian	Italy	DQA1*0102	80/95	0.83 (0.44, 1.59)	Patients from a single center in northern Italy. All were of European ancestry and resided in the Padova area of northern Italy.

Khor 2023, 37325616	Asian	Japan	DQA1*040101	2328/1653	1.75 (1.35, 2.27)	Japan PBC-GWAS Consortium
Mullarkey 2005, 15713222	Caucasian	USA	DQA1*0401	72/381	3.04 (1.39, 6.37)	Patients with PBC were recruited from the practices of gastroenterologists primarily from Washington State and the Seattle area. Control healthy women were recruited from the Seattle area
Donaldson 2006, 16941709	Caucasian	UK	DQA1*0401	412/236	3.59 (1.67, 7.73)	Patients from 2 major UK centers, all patients and controls were of northern European ancestry
Donaldson 2001, 11171832	Caucasian	UK	DQA1*0401	164/102	5.38 (1.57, 18.42)	All were of northern European ancestry and resident within the Newcastle area
Donaldson 2006, 16941709	Caucasian	Italy	DQA1*0401	80/95	2.67 (1.02, 6.98)	Patients from a single center in northern Italy. All were of European ancestry and resided in the Padova area of northern Italy.
Khor 2023, 37325616	Asian	Japan	DQB1*030101	2328/1653	0.54 (0.45, 0.65)	Japan PBC-GWAS Consortium
Umemura 2012, 21953406	Asian	Japan	DQB1*0301	229/523	0.44 (0.29, 0.69)	The Shinshu PBC Study Group between January 2005 and September 2010
Yasunami 2017, 28894202	Asian	Japan	DQB1*0301	1200/1196	0.50 (0.40, 0.63)	the National Hospital Organization Study Group for Liver Disease in Japan (NHOSLJ)
Zhao 2013, 23809616	Asian	China	DQB1*0301	145/500	0.42 (0.27, 0.65)	Beijing You'an Hospital were studied between September 2008 and October 2010, China
Mullarkey 2005, 15713222	Caucasian	USA	DQB1*0301	72/381	0.47 (0.22, 0.94)	Patients with PBC were recruited from the practices of gastroenterologists primarily from Washington State and the Seattle area. Control healthy women were recruited from the Seattle area
Underhill 1992, 1359995	Caucasian	UK	DQB1*0301	83/181	0.89 (0.51, 1.56)	White British PSC patients From the Institute of Liver Studies, King's College Hospital and King's College School of Medicine and Dentistry, England and white Europeans was drawn from laboratory and clinical staff
Donaldson 2006, 16941709	Caucasian	UK	DQB1*0301	412/236	0.70 (0.50, 0.99)	Patients from 2 major UK centers, all patients and controls were of northern European ancestry
Donaldson 2006, 16941709	Caucasian	Italy	DQB1*0301	80/95	0.49 (0.27, 0.91)	Patients from a single center in northern Italy. All were of European ancestry and resided in the Padova area of northern Italy.
Khor 2023, 37325616	Asian	Japan	DQB1*040101	2328/1653	1.44 (1.27, 1.63)	Japan PBC-GWAS Consortium
Umemura 2012, 21953406	Asian	Japan	DQB1*0401	229/523	1.45 (1.07, 1.95)	The Shinshu PBC Study Group between January 2005 and September 2010
Yasunami 2017, 28894202	Asian	Japan	DQB1*0401	1200/1196	1.50 (1.25, 1.80)	the National Hospital Organization Study Group for Liver Disease in Japan (NHOSLJ)
Zhao 2013, 23809616	Asian	China	DQB1*0401	145/500	0.76 (0.39, 1.47)	Beijing You'an Hospital were studied between September 2008 and October 2010, China
Khor 2023, 37325616	Asian	Japan	DQB1*040201	2328/1653	1.56 (1.23, 1.99)	Japan PBC-GWAS Consortium
Yasunami 2017, 28894202	Asian	Japan	DQB1*0402	1200/1196	1.64 (1.24, 2.18)	the National Hospital Organization Study Group for Liver Disease in Japan (NHOSLJ)
Zhao 2013, 23809616	Asian	China	DQB1*0402	145/500	0.86 (0.18, 4.10)	Beijing You'an Hospital were studied between September 2008 and October 2010, China
Wassmuth 2002, 12144621	Caucasian	Sweden	DQB1*0402	99/158	3.32 (1.74, 6.32)	Unrelated patients with PBC from Sweden (University Hospitals of Lund, Malmö, Göteborg, Stockholm, Örebro, Uppsala and Umeå)
Underhill 1992, 1359995	Caucasian	UK	DQB1*0402	83/181	3.55 (1.22, 10.32)	White British PSC patients From the Institute of Liver Studies, King's College Hospital and King's College School of Medicine and Dentistry, England and white Europeans was drawn from laboratory and clinical staff
Donaldson 2006, 16941709	Caucasian	UK	DQB1*0402	412/236	3.02 (1.44, 6.29)	Patients from 2 major UK centers, all patients and controls were of northern European ancestry
Donaldson 2001, 11171832	Caucasian	UK	DQB1*0402	164/102	8.99 (2.08, 38.84)	All were of northern European ancestry and resident within the Newcastle area
Donaldson 2006, 16941709	Caucasian	Italy	DQB1*0402	80/95	3.42 (1.26, 9.30)	Patients from a single center in northern Italy. All were of European ancestry and resided in the Padova area of northern Italy.
Khor 2023, 37325616	Asian	Japan	DQB1*050101	2328/1653	0.76 (0.63, 0.91)	Japan PBC-GWAS Consortium
Zhao 2013, 23809616	Asian	China	DQB1*0501	145/500	0.95 (0.50, 1.81)	Beijing You'an Hospital were studied between September 2008 and October 2010, China
Underhill 1992, 1359995	Caucasian	UK	DQB1*0501	83/181	1.52 (0.88, 2.66)	White British PSC patients From the Institute of Liver Studies, King's College Hospital and King's College School of Medicine and Dentistry, England and white Europeans was drawn from laboratory and clinical staff

Zhao 2013, 23809616	Asian	China	DQB1*0503	145/500	1.20 (0.68, 2.11)	Beijing You'an Hospital were studied between September 2008 and October 2010, China
Underhill 1992, 1359995	Caucasian	UK	DQB1*0503	83/181	2.04 (0.76, 5.49)	White British PSC patients From the Institute of Liver Studies, King's College Hospital and King's College School of Medicine and Dentistry, England and white Europeans was drawn from laboratory and clinical staff
Wassmuth 2002, 12144621	Caucasian	Sweden	DQB1*0503	99/158	4.59 (1.33, 15.85)	Unrelated patients with PBC from Sweden (University Hospitals of Lund, Malmö, Göteborg, Stockholm, Örebro, Uppsala and Umeå)
Khor 2023, 37325616	Asian	Japan	DQB1*060101	2328/1653	1.46 (1.32, 1.63)	Japan PBC-GWAS Consortium
Umemura 2012, 21953406	Asian	Japan	DQB1*0601	229/523	1.75 (1.32, 2.30)	The Shinshu PBC Study Group between January 2005 and September 2010
Yasunami 2017, 28894202	Asian	Japan	DQB1*0601	1200/1196	1.51 (1.27, 1.78)	the National Hospital Organization Study Group for Liver Disease in Japan (NHOSLJ)
Zhao 2013, 23809616	Asian	China	DQB1*0601	145/500	2.08 (1.37, 3.16)	Beijing You'an Hospital were studied between September 2008 and October 2010, China
Underhill 1992, 1359995	Caucasian	UK	DQB1*0601	83/181	1.66 (0.36, 7.59)	White British PSC patients From the Institute of Liver Studies, King's College Hospital and King's College School of Medicine and Dentistry, England and white Europeans was drawn from laboratory and clinical staff
Mullarkey 2005, 15713222	Caucasian	USA	DQB1*0601	72/381	3.31 (0.50, 17.40)	Patients with PBC were recruited from the practices of gastroenterologists primarily from Washington State and the Seattle area. Control healthy women were recruited from the Seattle area
Khor 2023, 37325616	Asian	Japan	DQB1*060201	2328/1653	0.57 (0.46, 0.69)	Japan PBC-GWAS Consortium
Umemura 2012, 21953406	Asian	Japan	DQB1*0602	229/523	0.61 (0.41, 0.91)	The Shinshu PBC Study Group between January 2005 and September 2010
Zhao 2013, 23809616	Asian	China	DQB1*0602	145/500	1.15 (0.71, 1.86)	Beijing You'an Hospital were studied between September 2008 and October 2010, China
Underhill 1992, 1359995	Caucasian	UK	DQB1*0602	83/181	0.64 (0.34, 1.19)	White British PSC patients From the Institute of Liver Studies, King's College Hospital and King's College School of Medicine and Dentistry, England and white Europeans was drawn from laboratory and clinical staff
Donaldson 2001, 11171832	Caucasian	UK	DQB1*0602	164/102	0.91 (0.51, 1.65)	All were of northern European ancestry and resident within the Newcastle area
Mullarkey 2005, 15713222	Caucasian	USA	DQB1*0602	72/381	0.38 (0.16, 0.81)	Patients with PBC were recruited from the practices of gastroenterologists primarily from Washington State and the Seattle area. Control healthy women were recruited from the Seattle area
Zhao 2013, 23809616	Asian	China	DQB1*0603	145/500	0.44 (0.13, 1.48)	Beijing You'an Hospital were studied between September 2008 and October 2010, China
Underhill 1992, 1359995	Caucasian	UK	DQB1*0603	83/181	1.88 (0.75, 4.73)	White British PSC patients From the Institute of Liver Studies, King's College Hospital and King's College School of Medicine and Dentistry, England and white Europeans was drawn from laboratory and clinical staff
Mullarkey 2005, 15713222	Caucasian	USA	DQB1*0603	72/381	1.94 (0.94, 3.84)	Patients with PBC were recruited from the practices of gastroenterologists primarily from Washington State and the Seattle area. Control healthy women were recruited from the Seattle area
Khor 2023, 37325616	Asian	Japan	DQB1*060401	2328/1653	0.26 (0.20, 0.33)	Japan PBC-GWAS Consortium
Umemura 2012, 21953406	Asian	Japan	DQB1*0604	229/523	0.35 (0.17, 0.72)	The Shinshu PBC Study Group between January 2005 and September 2010
Yasunami 2017, 28894202	Asian	Japan	DQB1*0604	1200/1196	0.19 (0.13, 0.28)	the National Hospital Organization Study Group for Liver Disease in Japan (NHOSLJ)
Underhill 1992, 1359995	Caucasian	UK	DQB1*0604	83/181	1.10 (0.40, 3.03)	White British PSC patients From the Institute of Liver Studies, King's College Hospital and King's College School of Medicine and Dentistry, England and white Europeans was drawn from laboratory and clinical staff
Mullarkey 2005, 15713222	Caucasian	USA	DQB1*0604	72/381	0.41 (0.05, 1.72)	Patients with PBC were recruited from the practices of gastroenterologists primarily from Washington State and the Seattle area. Control healthy women were recruited from the Seattle area
Gregory 1993, 7909617	Caucasian	UK	DRB1*01	130/363	1.19 (0.77, 1.85)	Local healthy volunteers attending the local blood transfusion service from University of Newcastle upon Tyne.
Invernizzi 2003, 12663229	Caucasian	Italy	DRB1*01	186/558	0.62 (0.35, 1.11)	Patients attending the centre in Milan (Ospedale San Paolo) between June 1974 and December 1998
Invernizzi 2008, 19003916	Caucasian	Italy	DRB1*01	664/1992	1.20 (0.88, 1.62)	the Italian PBC Genetic Study Group

Khor 2023, 37325616	Asian	Japan	DRB1*010101	2328/1653	0.72 (0.59, 0.87)	Japan PBC-GWAS Consortium
Nakamura 2010, 20374297	Asian	Japan	DRB1*0101	334/258	1.25 (0.71, 2.21)	the National Hospital Organization Study Group for Liver Disease in Japan (NHOSLJ)
Zhao 2013, 23809616	Asian	China	DRB1*0101	145/500	0.73 (0.30, 1.79)	Beijing You'an Hospital were studied between September 2008 and October 2010, China
Li 2021, 34016546	Asian	China	DRB1*0101	52/126	2.50 (0.71, 8.81)	Three families with at least one of FDRs diagnosed as PBC were recruited in Tianjin, China
Invernizzi 2003, 12663229	Caucasian	Italy	DRB1*03	186/558	0.63 (0.38, 1.05)	Patients attending the centre in Milan (Ospedale San Paolo) between June 1974 and December 1998
Invernizzi 2008, 19003916	Caucasian	Italy	DRB1*03	664/1992	1.44 (1.07, 1.93)	the Italian PBC Genetic Study Group
Clemente 2017, 28588884	Caucasian	Italy	DRB1*03	20/89	3.02 (1.48, 6.16)	An independent Sardinian cohort
Nakamura 2010, 20374297	Asian	Japan	DRB1*0301	334/258	2.32 (0.09, 57.1)	the National Hospital Organization Study Group for Liver Disease in Japan (NHOSLJ)
Zhao 2013, 23809616	Asian	China	DRB1*0301	145/500	1.78 (1.00, 3.19)	Beijing You'an Hospital were studied between September 2008 and October 2010, China
Li 2021, 34016546	Asian	China	DRB1*0301	52/126	0.93 (0.32, 2.67)	Three families with at least one of FDRs diagnosed as PBC were recruited in Tianjin, China
Zhao 2013, 23809616	Asian	China	DRB1*04	145/500	1.04 (0.66, 1.65)	Beijing You'an Hospital were studied between September 2008 and October 2010, China
Gregory 1993, 7909617	Caucasian	UK	DRB1*04	130/363	1.14 (0.76, 1.72)	Local healthy volunteers attending the local blood transfusion service from University of Newcastle upon Tyne.
Invernizzi 2003, 12663229	Caucasian	Italy	DRB1*04	186/558	0.66 (0.36, 1.21)	Patients attending the centre in Milan (Ospedale San Paolo) between June 1974 and December 1998
Invernizzi 2008, 19003916	Caucasian	Italy	DRB1*04	664/1992	1.10 (0.82, 1.49)	the Italian PBC Genetic Study Group
Zepeda-gomez 2009, 19811438	Other	Mexico	DRB1*04	16/99	0.76 (0.34, 1.70)	Recruited from the Hepatology Clinic at the Instituto Nacional de Ciencias Medicas y Nutrición, from august 2002 through September 2003
Nakamura 2010, 20374297	Asian	Japan	DRB1*0401	334/258	0.66 (0.22, 1.97)	the National Hospital Organization Study Group for Liver Disease in Japan (NHOSLJ)
Zhao 2013, 23809616	Asian	China	DRB1*0401	145/500	1.30 (0.34, 4.96)	Beijing You'an Hospital were studied between September 2008 and October 2010, China
Li 2021, 34016546	Asian	China	DRB1*0401	52/126	0.81 (0.08, 7.84)	Three families with at least one of FDRs diagnosed as PBC were recruited in Tianjin, China
Khor 2023, 37325616	Asian	Japan	DRB1*040501	2328/1653	1.44 (1.27, 1.63)	Japan PBC-GWAS Consortium
Nakamura 2010, 20374297	Asian	Japan	DRB1*0405	334/258	1.53 (1.11, 2.11)	the National Hospital Organization Study Group for Liver Disease in Japan (NHOSLJ)[†]
Yasunami 2017, 28894202	Asian	Japan	DRB1*0405	1200/1196	1.49 (1.24, 1.78)	the National Hospital Organization Study Group for Liver Disease in Japan (NHOSLJ)[†]
Umemura 2012, 21953406	Asian	Japan	DRB1*0405	229/523	1.38 (1.02, 1.87)	The Shinshu PBC Study Group between January 2005 and September 2010
Zhao 2013, 23809616	Asian	China	DRB1*0405	145/500	0.78 (0.40, 1.50)	Beijing You'an Hospital were studied between September 2008 and October 2010, China
Li 2021, 34016546	Asian	China	DRB1*0405	52/126	0.59 (0.16, 2.15)	Three families with at least one of FDRs diagnosed as PBC were recruited in Tianjin, China
Khor 2023, 37325616	Asian	Japan	DRB1*040601	2328/1653	0.56 (0.41, 0.74)	Japan PBC-GWAS Consortium
Nakamura 2010, 20374297	Asian	Japan	DRB1*0406	334/258	0.46 (0.21, 0.99)	the National Hospital Organization Study Group for Liver Disease in Japan (NHOSLJ)
Zhao 2013, 23809616	Asian	China	DRB1*0406	145/500	2.03 (0.83, 4.93)	Beijing You'an Hospital were studied between September 2008 and October 2010, China
Li 2021, 34016546	Asian	China	DRB1*0406	52/126	0.40 (0.05, 3.35)	Three families with at least one of FDRs diagnosed as PBC were recruited in Tianjin, China
Gregory 1993, 7909617	Caucasian	UK	DRB1*07	130/363	1.06 (0.63, 1.80)	Local healthy volunteers attending the local blood transfusion service from University of Newcastle upon Tyne.
Invernizzi 2003, 12663229	Caucasian	Italy	DRB1*07	186/558	0.61 (0.38, 0.97)	Patients attending the centre in Milan (Ospedale San Paolo) between June 1974 and December 1998
Invernizzi 2008, 19003916	Caucasian	Italy	DRB1*07	664/1992	1.53 (1.20, 1.96)	the Italian PBC Genetic Study Group
Almasio 2016, 28070198	Caucasian	Italy	DRB1*07	28/237	5.03 (2.13, 11.87)	PBC patients were recruited at the gastroenterology and liver unit of Azienda Ospedaliera Policlinico Universitario P. Giaccone of Palermo, Italy, from January 2001

Nakamura 2010, 20374297	Asian	Japan	DRB1*0701	334/258	0.77 (0.19, 3.09)	the National Hospital Organization Study Group for Liver Disease in Japan (NHOSLJ)
Zhao 2013, 23809616	Asian	China	DRB1*0701	145/500	1.87 (1.24, 2.82)	Beijing You'an Hospital were studied between September 2008 and October 2010, China
Li 2021, 34016546	Asian	China	DRB1*0701	52/126	2.12 (1.11, 4.07)	Three families with at least one of FDRs diagnosed as PBC were recruited in Tianjin, China
Zhao 2013, 23809616	Asian	China	DRB1*08	145/500	2.88 (1.77, 4.69)	Beijing You'an Hospital were studied between September 2008 and October 2010, China
Wassmuth 2002, 12144621	Caucasian	Sweden	DRB1*08	99/158	3.22 (1.71, 3.18)	Unrelated patients with PBC from Sweden (University Hospitals of Lund, Malmö, Göteborg, Stockholm, Örebro, Uppsala and Umeå)
Mullarkey 2005, 15713222	Caucasian	USA	DRB1*08	72/381	2.55 (1.18, 5.24)	Patients with PBC were recruited from the practices of gastroenterologists primarily from Washington State and the Seattle area. Control healthy women were recruited from the Seattle area
Invernizzi 2003, 12663229	Caucasian	Italy	DRB1*08	186/558	1.54 (0.77, 3.10)	Patients attending the centre in Milan (Ospedale San Paolo) between June 1974 and December 1998
Invernizzi 2008, 19003916	Caucasian	Italy	DRB1*08	664/1992	3.30 (2.18, 4.99)	the Italian PBC Genetic Study Group
Donaldson 2006, 16941709	Caucasian	Italy	DRB1*08	80/95	3.15 (1.15, 8.62)	Patients from a single center in northern Italy. All were of European ancestry and resided in the Padova area of northern Italy.
Gordon 1999, 10453936	Caucasian	UK	DRB1*08	91/213	2.51 (1.01, 6.25)	North of England Caucasian patients were recruited
Donaldson 2006, 16941709	Caucasian	UK	DRB1*08	412/236	3.12 (1.55, 6.28)	Patients from 2 major UK centers, all patients and controls were of northern European ancestry
Gregory 1993, 7909617	Caucasian	UK	DRB1*08	130/363	2.26 (1.28, 4.00)	Local healthy volunteers attending the local blood transfusion service from University of Newcastle upon Tyne[†]
Mehal 1994, 7927254	Caucasian	UK	DRB1*08	64/61	1.27 (0.53, 3.07)	Unrelated patients attending specialist clinics in Newcastle-Upon-Tyne[†]
Nakamura 2010, 20374297	Asian	Japan	DRB1*0801	334/258	2.32 (0.09, 57.1)	the National Hospital Organization Study Group for Liver Disease in Japan (NHOSLJ)
Mullarkey 2005, 15713222	Caucasian	USA	DRB1*0801	72/381	2.36 (1.00, 5.23)	Patients with PBC were recruited from the practices of gastroenterologists primarily from Washington State and the Seattle area. Control healthy women were recruited from the Seattle area
Donaldson 2001, 11171832	Caucasian	UK	DRB1*0801	164/102	5.94 (1.74, 20.2)	All were of northern European ancestry and resident within the Newcastle area
Khor 2023, 37325616	Asian	Japan	DRB1*080201	2328/1653	1.45 (1.18, 1.80)	Japan PBC-GWAS Consortium
Nakamura 2010, 20374297	Asian	Japan	DRB1*0802	334/258	1.62 (0.91, 2.88)	the National Hospital Organization Study Group for Liver Disease in Japan (NHOSLJ)
Zhao 2013, 23809616	Asian	China	DRB1*0802	145/500	2.32 (0.38, 14.0)	Beijing You'an Hospital were studied between September 2008 and October 2010, China
Mullarkey 2005, 15713222	Caucasian	USA	DRB1*0802	72/381	5.35 (0.07, 421.19)	Patients with PBC were recruited from the practices of gastroenterologists primarily from Washington State and the Seattle area. Control healthy women were recruited from the Seattle area
Khor 2023, 37325616	Asian	Japan	DRB1*080302	2328/1653	1.78 (1.54, 2.06)	Japan PBC-GWAS Consortium
Umemura 2012, 21953406	Asian	Japan	DRB1*0803	229/523	2.22 (1.53, 3.20)	The Shinshu PBC Study Group between January 2005 and September 2010
Nakamura 2010, 20374297	Asian	Japan	DRB1*0803	334/258	2.24 (1.48, 3.41)	the National Hospital Organization Study Group for Liver Disease in Japan (NHOSLJ)[†]
Yasunami 2017, 28894202	Asian	Japan	DRB1*0803	1200/1196	1.75 (1.42, 2.16)	the National Hospital Organization Study Group for Liver Disease in Japan (NHOSLJ)[†]
Zhao 2013, 23809616	Asian	China	DRB1*0803	145/500	2.86 (1.74, 4.71)	Beijing You'an Hospital were studied between September 2008 and October 2010, China
Li 2021, 34016546	Asian	China	DRB1*0803	52/126	1.40 (0.60, 3.27)	Three families with at least one of FDRs diagnosed as PBC were recruited in Tianjin, China
Nakamura 2010, 20374297	Asian	Japan	DRB1*0901	334/258	0.96 (0.69, 1.34)	the National Hospital Organization Study Group for Liver Disease in Japan (NHOSLJ)
Zhao 2013, 23809616	Asian	China	DRB1*0901	145/500	0.76 (0.47, 1.22)	Beijing You'an Hospital were studied between September 2008 and October 2010, China
Li 2021, 34016546	Asian	China	DRB1*0901	52/126	0.76 (0.38, 1.52)	Three families with at least one of FDRs diagnosed as PBC were recruited in Tianjin, China
Gregory 1993, 7909617	Caucasian	UK	DRB1*10	130/363	1.12 (0.21, 5.84)	Local healthy volunteers attending the local blood transfusion service from University of Newcastle upon Tyne.
Invernizzi 2003, 12663229	Caucasian	Italy	DRB1*10	186/558	0.80 (0.23, 2.84)	Patients attending the centre in Milan (Ospedale San Paolo) between June 1974 and December 1998

Invernizzi 2008, 19003916	Caucasian	Italy	DRB1*10	664/1992	1.66 (0.79, 3.48)	the Italian PBC Genetic Study Group
Li 2021, 34016546	Asian	China	DRB1*1001	52/126	1.22 (0.22, 6.74)	Three families with at least one of FDRs diagnosed as PBC were recruited in Tianjin, China
Zhao 2013, 23809616	Asian	China	DRB1*1001	145/500	1.95 (0.64, 5.91)	Beijing You'an Hospital were studied between September 2008 and October 2010, China
Nakamura 2010, 20374297	Asian	Japan	DRB1*1001	334/258	1.81 (0.46, 7.03)	the National Hospital Organization Study Group for Liver Disease in Japan (NHOSLJ)
Gregory 1993, 7909617	Caucasian	UK	DRB1*11	130/363	1.09 (0.54, 2.19)	Local healthy volunteers attending the local blood transfusion service from University of Newcastle upon Tyne.
Wassmuth 2002, 12144621	Caucasian	Sweden	DRB1*11	99/158	0.94 (0.09, 0.65)	Unrelated patients with PBC from Sweden (University Hospitals of Lund, Malmö, Göteborg, Stockholm, Örebro, Uppsala and Umeå)
Invernizzi 2003, 12663229	Caucasian	Italy	DRB1*11	186/558	0.64 (0.36, 1.13)	Patients attending the centre in Milan (Ospedale San Paolo) between June 1974 and December 1998
Donaldson 2006, 16941709	Caucasian	UK	DRB1*11	412/236	0.97 (0.55, 1.73)	Patients from 2 major UK centers, all patients and controls were of northern European ancestry
Invernizzi 2008, 19003916	Caucasian	Italy	DRB1*11	664/1992	0.37 (0.29, 0.47)	the Italian PBC Genetic Study Group
Almasio 2016, 28070198	Caucasian	Italy	DRB1*11	28/237	0.29 (0.10, 0.86)	PBC patients were recruited at the gastroenterology and liver unit of Azienda Ospedaliera Policlinico Universitario P. Giaccone of Palermo, Italy, from January 2001
Clemente 2017, 28588884	Caucasian	Italy	DRB1*11	20/89	0.43 (0.13, 1.51)	An independent Sardinian cohort
Donaldson 2006, 16941709	Caucasian	Italy	DRB1*11	80/95	0.42 (0.22, 0.80)	Patients from a single center in northern Italy. All were of European ancestry and resided in the Padova area of northern Italy.
Khor 2023, 37325616	Asian	Japan	DRB1*110101	2328/1653	0.46 (0.30, 0.67)	Japan PBC-GWAS Consortium
Nakamura 2010, 20374297	Asian	Japan	DRB1*1101	334/258	0.28 (0.11, 0.66)	the National Hospital Organization Study Group for Liver Disease in Japan (NHOSLJ)
Umemura 2012, 21953406	Asian	Japan	DRB1*1101	229/523	0.35 (0.15, 0.83)	The Shinshu PBC Study Group between January 2005 and September 2010
Zhao 2013, 23809616	Asian	China	DRB1*1101	145/500	0.36 (0.15, 0.87)	Beijing You'an Hospital were studied between September 2008 and October 2010, China
Li 2021, 34016546	Asian	China	DRB1*1101	52/126	0.68 (0.22, 2.12)	Three families with at least one of FDRs diagnosed as PBC were recruited in Tianjin, China
Gregory 1993, 7909617	Caucasian	UK	DRB1*12	130/363	0.42 (0.09, 1.89)	Local healthy volunteers attending the local blood transfusion service from University of Newcastle upon Tyne.
Invernizzi 2003, 12663229	Caucasian	Italy	DRB1*12	186/558	0.47 (0.06, 3.77)	Patients attending the centre in Milan (Ospedale San Paolo) between June 1974 and December 1998
Invernizzi 2008, 19003916	Caucasian	Italy	DRB1*12	664/1992	1.05 (0.44, 2.50)	the Italian PBC Genetic Study Group
Khor 2023, 37325616	Asian	Japan	DRB1*120101	2328/1653	0.65 (0.50, 0.84)	Japan PBC-GWAS Consortium
Nakamura 2010, 20374297	Asian	Japan	DRB1*1201	334/258	0.83 (0.38, 1.77)	the National Hospital Organization Study Group for Liver Disease in Japan (NHOSLJ)
Zhao 2013, 23809616	Asian	China	DRB1*1201	145/500	0.64 (0.29, 1.39)	Beijing You'an Hospital were studied between September 2008 and October 2010, China
Li 2021, 34016546	Asian	China	DRB1*1201	52/126	0.34 (0.04, 2.80)	Three families with at least one of FDRs diagnosed as PBC were recruited in Tianjin, China
Nakamura 2010, 20374297	Asian	Japan	DRB1*1202	334/258	0.41 (0.15, 1.13)	the National Hospital Organization Study Group for Liver Disease in Japan (NHOSLJ)
Zhao 2013, 23809616	Asian	China	DRB1*1202	145/500	0.43 (0.21, 0.85)	Beijing You'an Hospital were studied between September 2008 and October 2010, China
Li 2021, 34016546	Asian	China	DRB1*1202	52/126	0.75 (0.31, 1.82)	Three families with at least one of FDRs diagnosed as PBC were recruited in Tianjin, China
Mullarkey 2005, 15713222	Caucasian	USA	DRB1*13	72/381	0.92 (0.46, 1.74)	Patients with PBC were recruited from the practices of gastroenterologists primarily from Washington State and the Seattle area. Control healthy women were recruited from the Seattle area
Gregory 1993, 7909617	Caucasian	UK	DRB1*13	130/363	0.13 (0.02, 1.00)	Local healthy volunteers attending the local blood transfusion service from University of Newcastle upon Tyne.
Donaldson 2006, 16941709	Caucasian	UK	DRB1*13	412/236	0.65 (0.42, 0.99)	Patients from 2 major UK centers, all patients and controls were of northern European ancestry
Donaldson 2001, 11171832	Caucasian	UK	DRB1*13	164/102	0.42 (0.21, 0.84)	All were of northern European ancestry and resident within the Newcastle area

Invernizzi 2003, 12663229	Caucasian	Italy	DRB1*13	186/558	0.64 (0.36, 1.13)	Patients attending the centre in Milan (Ospedale San Paolo) between June 1974 and December 1998
Invernizzi 2008, 19003916	Caucasian	Italy	DRB1*13	664/1992	0.74 (0.55, 1.01)	the Italian PBC Genetic Study Group
Almasio 2016, 28070198	Caucasian	Italy	DRB1*13	28/237	2.42 (0.89, 6.56)	PBC patients were recruited at the gastroenterology and liver unit of Azienda Ospedaliera Policlinico Universitario P. Giaccone of Palermo, Italy, from January 2001
Clemente 2017, 28588884	Caucasian	Italy	DRB1*13	20/89	2.26 (0.2, 25.51)	An independent Sardinian cohort
Donaldson 2006, 16941709	Caucasian	Italy	DRB1*13	80/95	0.25 (0.11, 0.59)	Patients from a single center in northern Italy. All were of European ancestry and resided in the Padova area of northern Italy.
Li 2021, 34016546	Asian	China	DRB1*1301	52/126	0.60 (0.07, 5.45)	Three families with at least one of FDRs diagnosed as PBC were recruited in Tianjin, China
Zhao 2013, 23809616	Asian	China	DRB1*1301	145/500	0.48 (0.14, 1.64)	Beijing You'an Hospital were studied between September 2008 and October 2010, China
Nakamura 2010, 20374297	Asian	Japan	DRB1*1301	334/258	0.77 (0.10, 5.49)	the National Hospital Organization Study Group for Liver Disease in Japan (NHOSLJ)
Mullarkey 2005, 15713222	Caucasian	USA	DRB1*1301	72/381	1.53 (0.69, 3.16)	Patients with PBC were recruited from the practices of gastroenterologists primarily from Washington State and the Seattle area. Control healthy women were recruited from the Seattle area
Khor 2023, 37325616	Asian	Japan	DRB1*130201	2328/1653	0.27 (0.21, 0.34)	Japan PBC-GWAS Consortium
Nakamura 2010, 20374297	Asian	Japan	DRB1*1302	334/258	0.38 (0.20, 0.72)	the National Hospital Organization Study Group for Liver Disease in Japan (NHOSLJ)
Umemura 2012, 21953406	Asian	Japan	DRB1*1302	229/523	0.49 (0.27, 0.91)	The Shinshu PBC Study Group between January 2005 and September 2010
Yasunami 2017, 28894202	Asian	Japan	DRB1*1302	1200/1196	0.24 (0.17, 0.33)	the National Hospital Organization Study Group for Liver Disease in Japan (NHOSLJ)
Zhao 2013, 23809616	Asian	China	DRB1*1302	145/500	0.46 (0.16, 1.33)	Beijing You'an Hospital were studied between September 2008 and October 2010, China
Mullarkey 2005, 15713222	Caucasian	USA	DRB1*1302	72/381	0.60 (1.15, 1.77)	Patients with PBC were recruited from the practices of gastroenterologists primarily from Washington State and the Seattle area. Control healthy women were recruited from the Seattle area
Zhao 2013, 23809616	Asian	China	DRB1*14	145/500	1.80 (0.92, 3.52)	Beijing You'an Hospital were studied between September 2008 and October 2010, China
Gregory 1993, 7909617	Caucasian	UK	DRB1*14	130/363	1.92 (0.77, 4.80)	Local healthy volunteers attending the local blood transfusion service from University of Newcastle upon Tyne.
Wassmuth 2002, 12144621	Caucasian	Sweden	DRB1*14	99/158	6.86 (1.70, 27.6)	Unrelated patients with PBC from Sweden (University Hospitals of Lund, Malmö, Göteborg, Stockholm, Örebro, Uppsala and Umeå)
Invernizzi 2003, 12663229	Caucasian	Italy	DRB1*14	186/558	0.64 (0.33, 1.25)	Patients attending the centre in Milan (Ospedale San Paolo) between June 1974 and December 1998
Invernizzi 2008, 19003916	Caucasian	Italy	DRB1*14	664/1992	1.45 (1.03, 2.05)	the Italian PBC Genetic Study Group
Almasio 2016, 28070198	Caucasian	Italy	DRB1*14	28/237	3.00 (1.01, 8.95)	PBC patients were recruited at the gastroenterology and liver unit of Azienda Ospedaliera Policlinico Universitario P. Giaccone of Palermo, Italy, from January 2001
Khor 2023, 37325616	Asian	Japan	DRB1*140301	2328/1653	0.29 (0.15, 0.52)	Japan PBC-GWAS Consortium
Yasunami 2017, 28894202	Asian	Japan	DRB1*1403	1200/1196	0.21 (0.09, 0.49)	the National Hospital Organization Study Group for Liver Disease in Japan (NHOSLJ)
Zhao 2013, 23809616	Asian	China	DRB1*1403	145/500	0.57 (0.07, 4.79)	Beijing You'an Hospital were studied between September 2008 and October 2010, China
Li 2021, 34016546	Asian	China	DRB1*1405	52/126	3.42 (1.16, 10.11)	Three families with at least one of FDRs diagnosed as PBC were recruited in Tianjin, China
Nakamura 2010, 20374297	Asian	Japan	DRB1*1405	334/258	1.16 (0.61, 2.21)	the National Hospital Organization Study Group for Liver Disease in Japan (NHOSLJ)
Zhao 2013, 23809616	Asian	China	DRB1*1405	145/500	2.06 (0.99, 4.29)	Beijing You'an Hospital were studied between September 2008 and October 2010, China
Gregory 1993, 7909617	Caucasian	UK	DRB1*15	130/363	0.88 (0.55, 1.41)	Local healthy volunteers attending the local blood transfusion service from University of Newcastle upon Tyne.
Donaldson 2006, 16941709	Caucasian	UK	DRB1*15	412/236	0.74 (0.50, 1.11)	Patients from 2 major UK centers, all patients and controls were of northern European ancestry
Mullarkey 2005, 15713222	Caucasian	USA	DRB1*15	72/381	0.54 (0.26, 1.05)	Patients with PBC were recruited from the practices of gastroenterologists primarily from Washington State and the Seattle area. Control healthy women were recruited from the Seattle area

Almasio 2016, 28070198	Caucasian	Italy	DRB1*15	28/237	0.21 (0.05, 0.92)	PBC patients were recruited at the gastroenterology and liver unit of Azienda Ospedaliera Policlinico Universitario P. Giaccone of Palermo, Italy, from January 2001
Donaldson 2006, 16941709	Caucasian	Italy	DRB1*15	80/95	0.96 (0.43, 2.13)	Patients from a single center in northern Italy. All were of European ancestry and resided in the Padova area of northern Italy.
Khor 2023, 37325616	Asian	Japan	DRB1*150101	2328/1653	0.59 (0.48, 0.72)	Japan PBC-GWAS Consortium
Li 2021, 34016546	Asian	China	DRB1*1501	52/126	1.12 (0.56, 2.26)	Three families with at least one of FDRs diagnosed as PBC were recruited in Tianjin, China
Zhao 2013, 23809616	Asian	China	DRB1*1501	145/500	0.91 (0.6, 1.38)	Beijing You'an Hospital were studied between September 2008 and October 2010, China
Nakamura 2010, 20374297	Asian	Japan	DRB1*1501	334/258	0.56 (0.37, 0.84)	the National Hospital Organization Study Group for Liver Disease in Japan (NHOSLJ)
Mullarkey 2005, 15713222	Caucasian	USA	DRB1*1501	72/381	0.42 (0.19, 0.87)	Patients with PBC were recruited from the practices of gastroenterologists primarily from Washington State and the Seattle area. Control healthy women were recruited from the Seattle area
Donaldson 2001, 11171832	Caucasian	UK	DRB1*1501	164/102	0.96 (0.54, 1.71)	All were of northern European ancestry and resident within the Newcastle area
Li 2021, 34016546	Asian	China	DRB1*1502	52/126	0.60 (0.13, 2.87)	Three families with at least one of FDRs diagnosed as PBC were recruited in Tianjin, China
Zhao 2013, 23809616	Asian	China	DRB1*1502	145/500	0.73 (0.33, 1.61)	Beijing You'an Hospital were studied between September 2008 and October 2010, China
Nakamura 2010, 20374297	Asian	Japan	DRB1*1502	334/258	1.41 (0.96, 2.06)	the National Hospital Organization Study Group for Liver Disease in Japan (NHOSLJ)
Mullarkey 2005, 15713222	Caucasian	USA	DRB1*1502	72/381	3.27 (0.49, 17.19)	Patients with PBC were recruited from the practices of gastroenterologists primarily from Washington State and the Seattle area. Control healthy women were recruited from the Seattle area
Li 2021, 34016546	Asian	China	DRB1*1602	52/126	0.60 (0.13, 2.87)	Three families with at least one of FDRs diagnosed as PBC were recruited in Tianjin, China
Zhao 2013, 23809616	Asian	China	DRB1*1602	145/500	0.65 (0.25, 1.73)	Beijing You'an Hospital were studied between September 2008 and October 2010, China
Nakamura 2010, 20374297	Asian	Japan	DRB1*1602	334/258	1.74 (0.53, 5.70)	the National Hospital Organization Study Group for Liver Disease in Japan (NHOSLJ)

[†]The participants were repeated.

Supplementary Table 6. Characteristics of the candidate-gene association studies on variants in HLA genes and risk of primary biliary cirrhosis with datasets less than three.

Study, PMID	Gene	Variants	Country	Cases/ Controls	OR (95% CI)
Invernizzi 2003, 12663229	HLA	A*01	Italy	186/558	0.95 (0.51, 1.75)
Ercilla 2008, 12731577	HLA	A*01	Spain	21/74	4.27 (1.5, 12.13)
Umemura 2012, 21953406	HLA	A*0201	Japan	229/523	1.63 (1.19, 2.24)
Yasunami 2017, 28894202	HLA	A*0201	Japan	1200/1196	1.51 (1.26, 1.81)
Invernizzi 2003, 12663229	HLA	B*07	Italy	186/558	0.52 (0.16, 1.74)
Neri 2003, 14567462	HLA	B*07	Italy	64/183	0.74 (0.31, 1.80)
Invernizzi 2003, 12663229	HLA	B*13	Italy	186/558	1.11 (0.37, 3.35)
Neri 2003, 14567462	HLA	B*13	Italy	64/183	0.56 (0.12, 2.62)
Invernizzi 2003, 12663229	HLA	B*14	Italy	186/558	1.68 (0.45, 6.30)
Neri 2003, 14567462	HLA	B*14	Italy	64/183	0.95 (0.19, 4.84)
Invernizzi 2003, 12663229	HLA	B*18	Italy	186/558	1.00 (0.49, 2.03)
Neri 2003, 14567462	HLA	B*18	Italy	64/183	0.51 (0.14, 1.82)
Invernizzi 2003, 12663229	HLA	B*27	Italy	186/558	2.03 (0.63, 6.59)
Neri 2003, 14567462	HLA	B*27	Italy	64/183	4.45 (0.73, 27.27)
Invernizzi 2003, 12663229	HLA	B*35	Italy	186/558	0.60 (0.35, 1.04)
Neri 2003, 14567462	HLA	B*35	Italy	64/183	0.50 (0.25, 1.00)
Invernizzi 2003, 12663229	HLA	B*38	Italy	186/558	1.84 (0.58, 5.89)
Neri 2003, 14567462	HLA	B*38	Italy	64/183	0.80 (0.25, 2.54)
Invernizzi 2003, 12663229	HLA	B*39	Italy	186/558	1.34 (0.44, 4.12)
Neri 2003, 14567462	HLA	B*39	Italy	64/183	1.47 (0.53, 4.11)
Invernizzi 2003, 12663229	HLA	B*44	Italy	186/558	0.60 (0.28, 1.30)
Neri 2003, 14567462	HLA	B*44	Italy	64/183	0.86 (0.43, 1.70)
Invernizzi 2003, 12663229	HLA	B*49	Italy	186/558	0.55 (0.07, 4.38)
Neri 2003, 14567462	HLA	B*49	Italy	64/183	0.95 (0.19, 4.84)
Invernizzi 2003, 12663229	HLA	B*50	Italy	186/558	0.90 (0.20, 4.14)
Neri 2003, 14567462	HLA	B*50	Italy	64/183	1.44 (0.26, 8.08)
Invernizzi 2003, 12663229	HLA	B*51	Italy	186/558	0.70 (0.38, 1.31)
Neri 2003, 14567462	HLA	B*51	Italy	64/183	1.52 (0.77, 2.99)
Invernizzi 2003, 12663229	HLA	B*52	Italy	186/558	1.25 (0.14, 11.27)
Neri 2003, 14567462	HLA	B*52	Italy	64/183	0.56 (0.12, 2.62)
Invernizzi 2003, 12663229	HLA	B*55	Italy	186/558	2.92 (0.84, 10.13)
Neri 2003, 14567462	HLA	B*55	Italy	64/183	1.15 (0.35, 3.81)
Invernizzi 2003, 12663229	HLA	B*56	Italy	186/558	1.67 (0.17, 16.17)
Neri 2003, 14567462	HLA	B*56	Italy	64/183	1.45 (0.35, 5.98)
Invernizzi 2003, 12663229	HLA	B*57	Italy	186/558	0.45 (0.06, 3.51)
Neri 2003, 14567462	HLA	B*57	Italy	64/183	1.44 (0.26, 8.08)
Invernizzi 2003, 12663229	HLA	B*58	Italy	186/558	5.05 (0.7, 36.27)
Neri 2003, 14567462	HLA	B*58	Italy	64/183	0.47 (0.06, 3.97)
Zhao 2013, 23809616	HLA	B*61	China	145/500	1.62 (0.98, 2.65)
Neri 2003, 14567462	HLA	B*61	Italy	64/183	0.71 (0.15, 3.41)
Underhill 1995, 7705806	HLA	DPB1*0301	UK	82/103	1.28 (0.65, 2.50)
Wassmuth 2002, 12144621	HLA	DPB1*0301	Sweden	99/158	1.85 (1.04, 3.28)
Underhill 1995, 7705806	HLA	DPB1*0402	UK	82/103	0.89 (0.41, 1.94)

Wassmuth 2002, 12144621	HLA	DPB1*0402	Sweden	99/158	0.42 (0.21, 0.85)
Zhao 2013, 23809616	HLA	DQB1*0201	China	145/500	1.78 (1.00, 3.19)
Underhill 1992, 1359995	HLA	DQB1*0201	UK	83/181	1.21 (0.72, 2.03)
Zhao 2013, 23809616	HLA	DQB1*0302	China	145/500	1.56 (0.88, 2.77)
Underhill 1992, 1359995	HLA	DQB1*0302	UK	83/181	0.63 (0.35, 1.15)
Zhao 2013, 23809616	HLA	DQB1*0303	China	145/500	0.77 (0.49, 1.21)
Underhill 1992, 1359995	HLA	DQB1*0303	UK	83/181	1.27 (0.51, 3.16)
Zhao 2013, 23809616	HLA	DQB1*0502	China	145/500	0.97 (0.53, 1.78)
Underhill 1992, 1359995	HLA	DQB1*0502	UK	83/181	0.72 (0.07, 7.06)
Zhao 2013, 23809616	HLA	DR1	China	145/500	0.85 (0.38, 1.90)
Zepeda 2009, 19811438	HLA	DR1	Mexico	16/99	2.05 (0.68, 6.19)
Invernizzi 2003, 12663229	HLA	DRB1*02	Italy	186/558	0.62 (0.38, 1.01)
Invernizzi 2008, 19003916	HLA	DRB1*02	Italy	664/1992	1.00 (0.77, 1.32)
Nakamura 2010, 20374297	HLA	DRB1*0403	Japan	334/258	0.65 (0.33, 1.24)
Zhao 2013, 23809616	HLA	DRB1*0403	China	145/500	0.62 (0.14, 2.84)
Nakamura 2010, 20374297	HLA	DRB1*0404	Japan	334/258	2.32 (0.09, 57.1)
Zhao 2013, 23809616	HLA	DRB1*0404	China	145/500	1.55 (0.47, 5.10)
Nakamura 2010, 20374297	HLA	DRB1*0407	Japan	334/258	0.77 (0.11, 5.49)
Zhao 2013, 23809616	HLA	DRB1*0407	China	145/500	0.57 (0.07, 4.79)
Nakamura 2010, 20374297	HLA	DRB1*0410	Japan	334/258	0.84 (0.36, 2.04)
Li 2021, 34016546	HLA	DRB1*0410	China	52/126	2.44 (0.15, 39.33)
Invernizzi 2003, 12663229	HLA	DRB1*09	Italy	186/558	0.63 (0.07, 5.24)
Invernizzi 2008, 19003916	HLA	DRB1*09	Italy	664/1992	3.77 (1.01, 14.08)
Li 2021, 34016546	HLA	DRB1*1407	China	52/126	2.44 (0.15, 39.33)
Zhao 2013, 23809616	HLA	DRB1*1407	China	145/500	0.49 (0.06, 4.01)
Li 2021, 34016546	HLA	DRB1*1454	China	52/126	0.80 (0.16, 4.05)
Zhao 2013, 23809616	HLA	DRB1*1454	China	145/500	1.50 (0.72, 3.11)
Invernizzi 2003, 12663229	HLA	A*02	Italy	186/558	0.89 (0.57, 1.39)
Umemura 2012, 21953406	HLA	A*0206	Japan	229/523	0.61 (0.39, 0.95)
Invernizzi 2003, 12663229	HLA	A*03	Italy	186/558	0.68 (0.34, 1.37)
Invernizzi 2003, 12663229	HLA	A*11	Italy	186/558	1.00 (0.45, 2.19)
Invernizzi 2003, 12663229	HLA	A*23	Italy	186/558	0.83 (0.10, 6.95)
Zhao 2013, 23809616	HLA	A*2301	China	145/500	7.06 (1.28, 38.97)
Invernizzi 2003, 12663229	HLA	A*24	Italy	186/558	1.06 (0.58, 1.93)
Invernizzi 2003, 12663229	HLA	A*25	Italy	186/558	1.11 (0.24, 5.20)
Invernizzi 2003, 12663229	HLA	A*26	Italy	186/558	0.93 (0.27, 3.25)
Invernizzi 2003, 12663229	HLA	A*28	Italy	186/558	0.88 (0.25, 3.04)
Invernizzi 2003, 12663229	HLA	A*29	Italy	186/558	0.44 (0.10, 1.91)
Invernizzi 2003, 12663229	HLA	A*30	Italy	186/558	1.70 (0.66, 4.38)
Invernizzi 2003, 12663229	HLA	A*31	Italy	186/558	0.33 (0.04, 2.49)
Zhao 2013, 23809616	HLA	A*3101	China	145/500	0.38 (0.13, 1.08)
Invernizzi 2003, 12663229	HLA	A*32	Italy	186/558	2.13 (0.95, 4.76)
Invernizzi 2003, 12663229	HLA	A*33	Italy	186/558	1.25 (0.26, 5.97)
Khor 2023, 37325616	HLA	B*070201	Japan	2328/1653	0.70 (0.58, 0.86)
Yasunami 2017, 28894202	HLA	B*0702	Japan	1200/1196	0.58 (0.44, 0.77)

Invernizzi 2003, 12663229	HLA	B*15	Italy	186/558	2.48 (1.09, 5.63)
Khor 2023, 37325616	HLA	B*150101	Japan	2328/1653	0.61 (0.51, 0.73)
Invernizzi 2003, 12663229	HLA	B*37	Italy	186/558	3.04 (0.72, 12.93)
Invernizzi 2003, 12663229	HLA	B*40	Italy	186/558	1.82 (0.64, 5.15)
Khor 2023, 37325616	HLA	B*400201	Japan	2328/1653	1.42 (1.20, 1.68)
Zhao 2013, 23809616	HLA	B*4002	China	145/500	0.68 (0.23, 2.02)
Zhao 2013, 23809616	HLA	B*4003	China	145/500	0.69 (0.08, 5.93)
Zhao 2013, 23809616	HLA	B*4006	China	145/500	2.24 (1.27, 3.93)
Invernizzi 2003, 12663229	HLA	B*41	Italy	186/558	7.65 (1.26, 46.33)
Khor 2023, 37325616	HLA	B*460101	Japan	2328/1653	1.37 (1.14, 1.65)
Invernizzi 2003, 12663229	HLA	B*47	Italy	186/558	2.50 (0.23, 27.86)
Invernizzi 2003, 12663229	HLA	B*53	Italy	186/558	2.50 (0.23, 27.86)
Neri 2003, 14567462	HLA	B*60	Italy	64/183	0.35 (0.04, 2.83)
Neri 2003, 14567462	HLA	B*62	Italy	64/183	2.12 (0.77, 5.84)
Neri 2003, 14567462	HLA	B*63	Italy	64/183	5.87 (0.52, 65.88)
Khor 2023, 37325616	HLA	C*010201	Japan	2328/1653	1.19 (1.07, 1.34)
Umemura 2012, 21953406	HLA	C*0303	Japan	229/523	1.48 (1.10, 1.99)
Umemura 2012, 21953406	HLA	C*0801	Japan	229/523	0.51 (0.32, 0.81)
Khor 2023, 37325616	HLA	C*140301	Japan	2328/1653	0.33 (0.27, 0.42)
Umemura 2012, 21953406	HLA	C*1403	Japan	229/523	0.38 (0.21, 0.70)
Umemura 2012, 21953406	HLA	C*1502	Japan	229/523	0.44 (0.21, 0.90)
Mehal 1994, 7927254	HLA	C4B2	UK	64/61	0.57 (0.26, 1.28)
Yasunami 2017, 28894202	HLA	DPA1*0103	Japan	1200/1196	0.55 (0.46, 0.67)
Khor 2023, 37325616	HLA	DPA1*020202	Japan	2328/1653	1.20 (1.06, 1.36)
Underhill 1995, 7705806	HLA	DPB1*0101	UK	82/103	1.02 (0.46, 2.27)
Underhill 1995, 7705806	HLA	DPB1*0601	UK	82/103	0.83 (0.14, 5.11)
Underhill 1995, 7705806	HLA	DPB1*0901	UK	82/103	2.63 (0.64, 10.86)
Underhill 1995, 7705806	HLA	DPB1*1001	UK	82/103	0.94 (0.31, 2.82)
Underhill 1995, 7705806	HLA	DPB1*1101	UK	82/103	2.16 (0.50, 9.34)
Underhill 1995, 7705806	HLA	DPB1*1301	UK	82/103	1.92 (0.31, 11.76)
Underhill 1995, 7705806	HLA	DPB1*1401	UK	82/103	0.41 (0.04, 4.03)
Underhill 1995, 7705806	HLA	DPB1*1501	UK	82/103	0.62 (0.06, 7.00)
Zhao 2013, 23809616	HLA	DQ2	China	145/500	2.07 (1.41, 3.05)
Zhao 2013, 23809616	HLA	DQ4	China	145/500	0.77 (0.42, 1.42)
Zhao 2013, 23809616	HLA	DQ5	China	145/500	1.05 (0.71, 1.57)
Zhao 2013, 23809616	HLA	DQ6	China	145/500	1.63 (1.13, 2.37)
Khor 2023, 37325616	HLA	DQA1*010101	Japan	2328/1653	0.73 (0.60, 0.88)
Mullarkey 2005, 15713222	HLA	DQA1*0101	USA	72/381	1.33 (0.70, 2.46)
Khor 2023, 37325616	HLA	DQA1*010301	Japan	2328/1653	1.45 (1.30, 1.61)
Mullarkey 2005, 15713222	HLA	DQA1*0103	USA	72/381	2.25 (1.10, 4.41)
Khor 2023, 37325616	HLA	DQA1*010401	Japan	2328/1653	1.38 (1.14, 1.68)
Khor 2023, 37325616	HLA	DQA1*030301	Japan	2328/1653	1.41 (1.26, 1.59)
Khor 2023, 37325616	HLA	DQA1*050501	Japan	2328/1653	0.50 (0.37, 0.67)
Mullarkey 2005, 15713222	HLA	DQB1*04	USA	72/381	3.02 (1.14, 6.18)
Zhao 2013, 23809616	HLA	DQB1*0202	China	145/500	1.85 (1.20, 2.83)

Underhill 1992, 1359995	HLA	DQB1*0504	UK	83/181	2.2 (0.14, 35.53)
Zhao 2013, 23809616	HLA	DQB1*0609	China	145/500	0.79 (0.22, 2.82)
Zhao 2013, 23809616	HLA	DR11	China	145/500	0.37 (0.17, 0.83)
Zhao 2013, 23809616	HLA	DR12	China	145/500	0.47 (0.28, 0.80)
Zhao 2013, 23809616	HLA	DR13	China	145/500	0.46 (0.20, 1.03)
Zhao 2013, 23809616	HLA	DR15	China	145/500	0.90 (0.61, 1.33)
Zhao 2013, 23809616	HLA	DRB1*0102	China	145/500	1.73 (0.31, 9.57)
Nakamura 2010, 20374297	HLA	DRB1*0409	Japan	334/258	2.32 (0.09, 57.1)
Mullarkey 2005, 15713222	HLA	DRB1*0804	USA	72/381	5.35 (0.07, 421.19)
Zhao 2013, 23809616	HLA	DRB1*1104	China	145/500	0.49 (0.06, 4.01)
Zhao 2013, 23809616	HLA	DRB1*1210	China	145/500	0.69 (0.08, 5.93)
Mullarkey 2005, 15713222	HLA	DRB1*1303	USA	72/381	0.47 (0.01, 3.36)
Nakamura 2010, 20374297	HLA	DRB1*1401	Japan	334/258	0.80 (0.44, 1.45)
Zhao 2013, 23809616	HLA	DRB1*1404	China	145/500	0.62 (0.14, 2.84)
Nakamura 2010, 20374297	HLA	DRB1*1406	Japan	334/258	0.34 (0.12, 1.00)
Gregory 1993, 7909617	HLA	DRB1*17	UK	130/363	1.17 (0.40, 3.39)
Gregory 1993, 7909617	HLA	DRB1*18	UK	130/363	2.81 (0.17, 45.19)
Gregory 1993, 7909617	HLA	DRB3*0101	UK	98/107	0.52 (0.26, 1.04)
Gregory 1993, 7909617	HLA	DRB3*0201	UK	98/107	1.27 (0.63, 2.57)
Gregory 1993, 7909617	HLA	DRB3*0301	UK	98/107	0.37 (0.11, 1.21)
Ercilla 2008, 12731577	HLA	DRw3	Spain	21/74	7.64 (2.6, 22.39)
Paziewska 2017, 28056976	HLA-DQA1	rs2187668	Polish	443/934	0.73 (0.56, 0.95)
Paziewska 2017, 28056976	HLA-DQB2	rs3213489	Polish	443/934	0.60 (0.50, 0.71)
Paziewska 2017, 28056976	HLA-DQA1	rs9272346	Polish	443/934	0.79 (0.67, 0.94)

Supplementary Table 7. Variants in HLA gene that were not associated with risk of PBC in meta-analysis.

Variant	Ethnicity	Data sets	Cases/ Controls	Risk estimates		Heterogeneity		***P* for Interaction**
				**OR (95%CI)**	***P***	***I^2***	***P***	
B*08	Caucasian[†]	3	271/815	1.09 (0.41, 2.93)	0.87	67.2%	0.05	
DQB1*0501	All ancestries	3	2556/2334	0.98 (0.63, 1.51)	0.91	64.6%	0.06	0.02
	Asian	2	2473/2153	0.77 (0.65, 0.92)	0.004	0.0%	0.52	
DQB1*0503	All ancestries	3	327/839	1.91 (0.92, 3.98)	0.08	50.0%	0.13	0.08
	Caucasian	2	182/339	2.80 (1.29, 6.08)	0.009	0.6%	0.32	
DQB1*0603	All ancestries	3	300/1062	1.31 (0.58, 2.98)	0.51	57.1%	0.10	0.03
	Caucasian	2	155/562	1.92 (1.10, 3.36)	0.02	0.0%	0.96	
DRB1*01	Caucasian[†]	3	980/2913	1.02 (0.72, 1.46)	0.91	51.9%	0.13	
DRB1*0101	Asian[†]	5	2859/2537	0.95 (0.61, 1.49)	0.83	54.1%	0.09	
DRB1*03	Caucasian[†]	3	870/2639	1.36 (0.65, 2.82)	0.42	85.2%	0.00	
DRB1*0301	Asian[†]	3	531/884	1.55 (0.94, 2.56)	0.09	0.0%	0.55	
DRB1*04	Caucasian[†]	5	1141/3512	1.02 (0.84, 1.24)	0.84	0.0%	0.55	
DRB1*0401	Asian[†]	3	531/884	0.86 (0.39, 1.90)	0.71	0.0%	0.74	
DRB1*0406	Asian[†]	4	2859/2537	0.71 (0.37, 1.35)	0.29	62.5%	0.05	
DRB1*07	Caucasian[†]	4	1008/3150	1.39 (0.73, 2.62)	0.32	86.3%	<0.001	
DRB1*0901	Asian[†]	3	531/884	0.87 (0.68, 1.12)	0.28	0.0%	0.66	
DRB1*10	Caucasian[†]	3	980/2913	1.34 (0.74, 2.43)	0.33	0.0%	0.61	
DRB1*1001	Asian[†]	3	531/884	1.73 (0.80, 3.73)	0.16	0.0%	0.90	
DRB1*12	Caucasian[†]	3	980/2913	0.78 (0.39, 1.59)	0.50	0.0%	0.51	
DRB1*1301	All ancestries	4	603/1265	1.03 (0.57, 1.86)	0.93	0.0%	0.42	0.10
	Asian	3	531/884	0.56 (0.22, 1.43)	0.22	0.0%	0.92	
DRB1*1502	All ancestries	4	603/1265	1.16 (0.70, 1.91)	0.57	27.6%	0.23	0.24
	Asian	3	531/884	1.07 (0.64, 1.80)	0.80	32.6%	0.25	
DRB1*1602	Asian[†]	3	531/884	0.88 (0.45, 1.74)	0.72	0.0%	0.39	

Supplementary Table 8. Variants in Non-HLA gene that were not associated with PBC in meta-analysis.

Gene	Variant	Allele*	Ethnicity	Data sets	Cases/ Controls	Risk estimates		Heterogeneity		P for Interaction
						OR (95%CI)	*P*	I^2	*P*	
VDR	rs731236	G/A	All ancestries	5	609/1015	1.09 (0.79, 1.49)	0.61	64.8%	0.02	0.89
			Asian	2	253/339	1.04 (0.57, 1.90)	0.89	30.8%	0.23	
			Caucasian	3	356/676	1.10 (0.72, 1.68)	0.67	79.8%	0.01	
VDR	rs7975232	A/C	All ancestries	5	609/1015	1.05 (0.77, 1.42)	0.77	73.4%	0.01	0.05
			Asian	2	253/339	1.40 (0.91, 2.17)	0.13	63.2%	0.10	
			Caucasian	3	356/676	0.88 (0.72, 1.07)	0.19	0.0%	0.52	
CTLA-4	rs733618	C/T	All ancestries	3	555/560	1.12 (0.88, 1.42)	0.37	24.3%	0.27	0.53
			Asian	2	308/268	1.06 (0.73, 1.52)	0.77	56.1%	0.13	
TNF-α	rs361525	A/G	Caucasian†	4	366/491	0.99 (0.56, 1.77)	0.97	28.3%	0.24	
FCRL3	rs7528684	G/A	All ancestries	3	678/831	0.71 (0.42, 1.20)	0.20	88.0%	<0.001	<0.001
			Caucasian	2	446/601	0.95 (0.78, 1.16)	0.61	0.0%	0.39	
IL-10	-592 C/A	C/A	All ancestries	4	407/444	1.11 (0.89, 1.38)	0.34	0.0%	0.42	0.55
			Asian	2	142/231	1.23 (0.90, 1.68)	0.20	0.0%	0.97	
			Caucasian	2	265/213	1.04 (0.66, 1.62)	0.87	50.8%	0.15	
IL-10	-819 C/T	C/T	All ancestries	3	236/303	0.79 (0.60, 1.03)	0.08	0.0%	0.96	0.80
			Asian	2	142/231	0.81 (0.59, 1.11)	0.18	0.0%	0.92	

* Risk allele versus reference allele. †Only Asian or Caucasian data were available for meta-analysis.

Supplementary Table 9. Subgroup analyses stratified by diagnostic items and genotyping methods for the associations with high heterogeneity.

**Variant**	**Subgroup**	**Data sets**	**OR**	***P***	**I^2**	***P***	***P* for interaction**	***P* for meta - regression**
DQB1*0402	**All**	8	2.26 (1.63, 3.15)	1.20×10^{-6}	55.6%	0.03		
	**Diagnosis of PBC**						**0.031**	**0.096**
	Two-out-of-three items	5	1.84 (1.37, 2.48)	5.01×10^{-5}	44.8%	0.124		
	All of the three items	3	3.66 (2.11, 6.33)	3.75×10^{-6}	0.0%	0.42		
	**Genotyping methods**						**0.043**	**0.115**
	PCR-SSO	6	2.89 (1.83, 4.56)	5.42×10^{-6}	56.3%	0.043		
DQB1*0602	**All**	6	0.68 (0.52, 0.88)	4.00×10^{-3}	50.0%	0.08		
	**Diagnosis of PBC**						0.301	
	Two-out-of-three items	5	0.65 (0.49, 0.86)	0.003	52.6%	0.077		
	**Genotyping methods**						**0.031**	
	PCR-SSO	4	0.64 (0.48, 0.84)	0.001	1.7%	0.384		
DQB1*0604	**All**	5	0.31 (0.20, 0.48)	1.42×10^{-7}	64.2%	0.03		
	**Genotyping methods**						0.380	
	PCR-SSO	4	0.38 (0.17, 0.83)	0.016	73.2%	0.011		
DRB1*1302	All	6	0.38 (0.25, 0.57)	2.72×10^{-6}	84.9%	<0.001		
	**Genotyping methods**						**<0.001**	
	PCR-SBT	3	0.29 (0.20, 0.42)	3.47×10^{-11}	21.1%	0.282		
DQA1*0102	**All**	6	0.61 (0.42, 0.90)	0.01	85.1%	<0.001		
	**Diagnosis of PBC**						**<0.001**	**0.007**
	Two-out-of-three items	3	0.41 (0.35, 0.48)	5.15×10^{-28}	16.4%	0.302		
	All of the three items	3	1.03 (0.76, 1.39)	0.864	0.0%	0.751		
	**Genotyping methods**						**0.017**	**0.302**
	PCR-SSO	5	0.70 (0.44, 1.11)	0.131	79.6%	0.001		
DRB1*11	**All**	8	0.58 (0.40, 0.85)	5.00×10^{-3}	64.4%	0.01		
	**Diagnosis of PBC**						0.141	
	Two-out-of-three items	4	0.41 (0.28, 0.59)	1.43×10^{-6}	17.1%	0.306		
	All of the three items	3	0.76 (0.43, 1.36)	0.358	60.2%	0.081		
	**Genotyping methods**						0.125	
	PCR-SSO	4	0.66 (0.41, 1.08)	0.099	34.2%	0.207		
	Reverse Line Blot Assay using PCR-SSO	2	0.46 (0.27, 0.78)	0.004	68.3%	0.076		
DRB1*13	**All**	9	0.66 (0.46, 0.93)	0.02	55.5%	0.02		
	**Diagnosis of PBC**						0.057	0.193
	Two-out-of-three items	4	1.03 (0.61, 1.73)	0.905	46.7%	0.131		
	All of the three items	4	0.41 (0.24, 0.71)	0.001	47.8%	0.125		
	**Genotyping methods**						**0.026**	**0.727**
	PCR-SSO	4	0.48 (0.28, 0.83)	0.008	45.8%	0.136		
	Reverse Line Blot Assay using PCR-SSO	2	0.72 (0.55, 0.94)	0.016	0.0%	0.636		
DRB1*14	**All**	6	1.68 (1.03, 2.74)	0.04	61.2%	0.02		
	**Diagnosis of PBC**						**0.020**	
	Two-out-of-three items	4	2.06 (1.23, 3.45)	0.006	47.6%	0.126		
	**Genotyping methods**						0.176	
	Reverse Line Blot Assay using PCR-SSO	2	1.02 (0.46, 2.25)	0.964	78.0%	0.033		

DQB1*0503	**All**	3	1.91 (0.92, 3.98)	0.08	50.0%	0.13	
	**Genotyping methods**						0.083
	PCR-SSO	2	2.80 (1.29, 6.08)	0.009	0.6%	0.316	
DQB1*0603	**All**	3	1.31 (0.58, 2.98)	0.51	57.1%	0.1	
	**Genotyping methods**						**0.031**
	PCR-SSO	2	1.92 (1.10, 3.36)	0.023	0.0%	0.957	
DRB1*01	**All**	3	1.02 (0.72, 1.46)	0.91	51.9%	0.13	
	**Genotyping methods**						0.485
	Reverse Line Blot Assay using PCR-SSO	2	0.90 (0.48, 1.71)	0.756	74.3%	0.049	
DRB1*0101	**All**	4	0.95 (0.61, 1.49)	0.83	54.1%	0.09	
	**Genotyping methods**						0.080
	PCR-SBT	3	1.20 (0.70, 2.04)	0.511	19.8%	0.287	
DRB1*03	**All**	3	1.36 (0.65, 2.82)	0.42	85.2%	0.001	
	**Diagnosis of PBC**						**0.012**
	Two-out-of-three items	2	1.94 (0.95, 3.95)	0.068	71.8%	0.06	
	**Genotyping methods**						**0.041**
	Reverse Line Blot Assay using PCR-SSO	2	0.98 (0.44, 2.20)	0.96	86.7%	0.006	
DRB1*0406	**All**	4	0.71 (0.37, 1.35)	0.29	62.5%	0.05	
	**Genotyping methods**						0.553
	PCR-SBT	3	0.80 (0.25, 2.56)	0.712	69.5%	0.038	
DRB1*07	**All**	4	1.39 (0.73, 2.62)	0.32	86.3%	<0.001	
	**Diagnosis of PBC**						**0.045**
	Two-out-of-three items	2	2.58 (0.81, 8.20)	0.108	85.4%	0.009	
	**Genotyping methods**						**0.006**
	Reverse Line Blot Assay using PCR-SSO	2	0.99 (0.40, 2.44)	0.976	91.5%	0.001	
rs9459874	**All**	3	1.17 (1.05, 1.32)	5.00×10^{-03}	81.7%	0	
	**Genotyping methods**						0.272
	Genotyping arrays	2	1.14 (1.01, 1.30)	0.037	87.9%	0.004	
rs7975232	**All**	5	1.05 (0.77, 1.42)	0.77	73.4%	0.01	
	**Genotyping methods**						0.259
	PCR-RFLP	4	1.11 (0.78, 1.59)	0.572	75.0%	0.007	
rs731236	**All**	5	1.09 (0.79, 1.49)	0.61	64.8%	0.02	
	**Genotyping methods**						**0.004**
	PCR-RFLP	4	0.95 (0.77, 1.19)	0.663	0.0%	0.413	

PCR-SBT, polymerase chain reaction with sequence-based typing; PCR-RFLP, polymerase chain reaction with restriction length fragment polymorphisms; PCR-SSO, polymerase chain reaction with sequence-specific oligonucleotide probes. The three items for diagnosing PBC are (i) cholestatic liver function tests,(ii) positive serum anti-mitochondrial antibody titer >1:40, and (iii) liver histology diagnostic of or compatible with PBC.

Supplementary Table 10. Details of protection from bias for variants significantly associated with risk of diseases.

Variant	Protection from bias	Reason for bias exemption	Reason for bias	Initial study influence		Deviation from HWE	OR<1.15 or OR>0.87	*P* value for small study bias	*P* value for publication bias
				OR (95%CI)	*P*				
rs1544410	A	NA		1.59 (1.34, 1.89)	1.55×10^{-7}	No	No	0.15	0.81
rs1864325	C	NA	Small study	0.79 (0.72, 0.86)	2.16×10^{-7}	No	No	0.01	1.00
rs231775	A	NA		1.30 (1.21, 1.40)	2.88×10^{-13}	No	No	0.32	0.24
rs3087243	A	NA		0.84 (0.76, 0.93)	0.001	No	No	0.29	0.90
rs3790567	A	Identified by GWAS	Initial study	1.23 (0.89, 1.69)	0.207	No	No	0.83	0.73
rs7574865	A	Identified by GWAS		1.26 (1.13, 1.41)	5.03×10^{-5}	No	No	0.27	1.00
rs9303277	A	Identified by GWAS		1.34 (1.21, 1.48)	6.75×10^{-9}	No	No	0.89	1.00
rs5742909	A	NA		0.77 (0.67, 0.89)	3.53×10^{-4}	No	No	0.54	0.68
-1082 G/A	C	NA	Initial study	1.41 (0.77, 2.56)	0.268	No	No	0.30	0.54
rs170183	C	NA	Publication bias	0.81 (0.72, 0.92)	1.00×10^{-3}	No	No	1.00	0.02
rs1800629	C	NA	Initial study	0.81 (0.64, 1.03)	0.086	No	No	1.00	0.88
rs231725	A	NA		1.31 (1.18, 1.45)	2.28×10^{-7}	No	No	0.46	0.93
rs9459874	A	NA		1.14 (1.01, 1.30)	0.037	No	No	1.00	0.35
rs9533090	A	NA		1.20 (1.06, 1.35)	4.00×10^{-3}	No	No	0.30	0.14
A*3303	A	NA		0.42 (0.36, 0.49)	7.19×10^{-25}	No	No	1.00	0.92
B*4403	A	NA		0.33 (0.28, 0.39)	2.23×10^{-34}	No	No	1.00	0.90
DPB1*0201	A	NA		0.71 (0.61, 0.83)	6.73×10^{-6}	No	No	0.45	1.00
DPB1*0401	C	NA	Initial study	0.56 (0.12, 2.65)	0.467	No	No	0.30	0.26
DPB1*0501	C	NA	Publication bias	1.41 (1.28, 1.54)	4.26×10^{-13}	No	No	0.30	0.04
DQA1*0102	C	NA	Initial study	0.67 (0.44, 1.02)	0.065	No	No	1.00	0.14
DQA1*0401	A	Identified by GWAS	Publication bias	2.34 (1.59, 3.44)	1.79×10^{-5}	No	No	0.22	0.01
DQB1*0301	A	Identified by GWAS		0.52 (0.47, 0.59)	3.72×10^{-27}	No	No	0.90	0.89
DQB1*0401	A	NA		1.41 (1.18, 1.67)	1.18×10^{-4}	No	No	0.73	0.25
DQB1*0402	A	Identified by GWAS	Publication bias	2.18 (1.55, 3.07)	7.16×10^{-6}	No	No	0.71	0.04
DQB1*0601	C	NA	Publication bias	1.50 (1.37, 1.64)	8.79×10^{-20}	No	No	0.26	0.07
DQB1*0602	A	Identified by GWAS		0.71 (0.54, 0.94)	0.015	No	No	1.00	0.52
DQB1*0604	A	NA		0.31 (0.18, 0.52)	1.44×10^{-5}	No	No	0.46	0.27

DRB1*0405	C	NA	Small study	1.38 (1.15, 1.65)	0.001	No	No	0.05	0.22
DRB1*0701	C	NA	Initial study	1.55 (0.62, 3.89)	0.346	No	No	1.00	0.45
DRB1*08	A	Identified by GWAS		2.90 (2.40, 3.49)	6.92×10^{-29}	No	No	0.20	0.27
DRB1*0801	A	NA		2.36 (1.06, 5.25)	0.036	No	No	1.00	0.86
DRB1*0802	C	NA	Small study, Publication bias	1.47 (1.21, 1.80)	1.26×10^{-4}	No	No	0.09	<0.001
DRB1*0803	A	Identified by GWAS		1.83 (1.58, 2.12)	1.56×10^{-15}	No	No	0.43	0.46
DRB1*11	A	Identified by GWAS		0.55 (0.37, 0.83)	4.00×10^{-3}	No	No	1.00	0.18
DRB1*1101	A	Identified by GWAS		0.44 (0.32, 0.61)	6.52×10^{-7}	No	No	0.64	1.00
DRB1*1201	C	NA	Initial study	0.69 (0.41, 1.18)	0.177	No	No	0.80	0.73
DRB1*1202	C	NA	Initial study	0.58 (0.30, 1.13)	0.107	No	No	0.83	1.00
DRB1*13	A	NA		0.72 (0.53, 0.99)	0.045	No	No	1.00	0.79
DRB1*1302	A	NA		0.38 (0.24, 0.60)	3.64×10^{-5}	No	No	1.00	0.88
DRB1*14	C	NA	Initial study, Small study	1.88 (0.92, 3.85)	0.083	No	No	0.06	0.34
DRB1*1403	A	NA		0.31 (0.17, 0.56)	9.98×10^{-5}	No	No	1.00	0.65
DRB1*1405	C	NA	Initial study	1.50 (0.86, 2.63)	0.154	No	No	0.27	0.30
DRB1*15	A	NA		0.67 (0.46, 0.98)	0.037	No	No	0.46	0.23
DRB1*1501	C	NA	Initial study	0.74 (0.54, 1.01)	0.058	No	No	0.37	0.71

Supplementary Table 11. Functional annotation for variants associated with PBC through HeploReg V4.1.

SNP	chr:pos (hg38)	Alt/ Ref	AFR _freq	AMR _freq	ASN _freq	EUR_ freq	Promoter histone marks[1]	Enhancer histone marks[2]	DNAse[3]	Motifs changed[4]	GENCODE genes	dbSNP func annot
rs3790567	1:67356694	G/A	0.26	0.72	0.75	0.76	SKIN	10 tissues	SKIN	Foxl1,THAP1	IL12RB2	intronic
rs231725	2:203875952	A/G	0.48	0.40	0.62	0.33		BLD		HNF6	CTLA4	
rs3087243	2:203874196	A/G	0.18	0.4	0.24	0.45		4 tissues	5 tissues		CTLA4	
rs231775	2:203867991	G/A	0.40	0.41	0.64	0.36	BLD, GI, THYM	4 tissues	BLD,BLD	AP-4,Rad21	CTLA4	missense
rs5742909	2:203867624	T/C	0.01	0.08	0.11	0.09	BLD	BLD, SKIN, THYM	BLD,THYM		CTLA4	
rs7574865	2:191099907	G/T	0.86	0.69	0.66	0.77		BLD		Foxj2,Foxp1	STAT4	intronic
rs1800629	6:31575254	A/G	0.10	0.07	0.06	0.14	7 tissues	12 tissues	BLD,BLD,BLD	ATF3,CCNT2,SP1	TNF	
rs9459874	6:167090639	T/C	0.39	0.40	0.37	0.43	BLD	6 tissues	BLD,BLD		CCR6	
rs1544410	12:47846052	T/C	0.28	0.27	0.07	0.4		4 tissues		4 altered motifs	VDR	intronic
rs9533090	13:42377313	T/C	0.25	0.44	0.07	0.49	BLD	BLD, GI, THYM	BLD,BLD		AKAP11	
rs1864325	17:45900461	AGT /C	0.02	0.19	0	0.23		BRST, BRN, MUS			MAPT	intronic
rs9303277	17:39820216	T/C	0.62	0.44	0.35	0.53		5 tissues	BLD,BLD	4 altered motifs	IKZF3	intronic
rs170183	21:36476036	A/G	0.05	0.40	0.38	0.54		LIV		HNF4	AP000695	intronic

[1]Evidence of local H3K4Me1 and H3K27Ac modification (cell lines/types: if >3, only the number is included).
[2]Evidence of local H3K4Me3 modification (cell lines/types: if >3, only the number is included).
[3]Evidence of chromatin hypersensitivity to DNase (cell lines/types: if >3, only the number is included).
[4]Evidence of alteration in regulatory motif (if >3, only the number is included).

Supplementary Table 12. Significant association in phenome-wide analysis of the novel variant using data from UK Biobank.

Variant	Position	Effect/other_Allele	MAF	Trait	Beta	*P*-value
rs231775	2:204732714	G/A	0.385	thyroid problem (not cancer)	1.009 (1.010, 1.008)	1.45×10^{-82}
				hypothyroidism/myxoedema	1.008 (1.009, 1.007)	4.52×10^{-72}
				E03 Other hypothyroidism	1.005 (1.004, 1.006)	3.20×10^{-38}
				E00-E07 Disorders of thyroid gland	1.005 (1.004, 1.006)	7.23×10^{-35}
				hyperthyroidism/thyrotoxicosis	1.002 (1.001, 1.002)	2.29×10^{-20}
				C43-C44 Melanoma and other malignant neoplasms of skin	0.997 (0.997, 0.998)	1.35×10^{-10}
				C44 Other malignant neoplasms of skin	0.998 (0.997, 0.998)	8.19×10^{-10}
				E05 Thyrotoxicosis [hyperthyroidism]	1.001 (1.000, 1.001)	8.86×10^{-6}
				Trunk predicted mass	0.975 (0.965, 0.985)	4.75×10^{-7}
				Trunk fat-free mass	0.974 (0.964, 0.984)	6.58×10^{-7}
				Whole body fat-free mass	0.952 (0.933, 0.972)	2.36×10^{-6}
				Arm predicted mass (left)	0.996 (0.995, 0.998)	2.55×10^{-6}
				Whole body water mass	0.965 (0.951, 0.980)	3.05×10^{-6}
				Arm fat-free mass (left)	0.996 (0.995, 0.998)	3.67×10^{-6}
				Basal metabolic rate	0.002 (0.000, 0.025)	4.62×10^{-6}
				Arm fat-free mass (right)	0.997 (0.995, 0.998)	9.65×10^{-6}
				Arm predicted mass (right)	0.997 (0.996, 0.998)	1.13×10^{-5}
				Red blood cell (erythrocyte) count	0.997 (0.996, 0.999)	2.02×10^{-5}
				Sitting height	0.976 (0.965, 0.987)	2.93×10^{-5}
				Number of self-reported non-cancer illnesses	1.015 (1.008, 1.022)	5.11×10^{-5}
				Weight	0.907 (0.864, 0.951)	5.36×10^{-5}
				Leg predicted mass (left)	0.992 (0.989, 0.996)	7.96×10^{-5}
				Leg fat-free mass (left)	0.992 (0.988, 0.996)	7.98×10^{-5}

Supplementary Table 13. Variants significant associated with risk of primary biliary cirrhosis in meta-analysis with two datasets.

Gene	Variants	Cases	Controls	Risk estimates		Heterogeneity	
				OR (95%CI)	***P***	I^2	***P***
CCR6	rs6905911	4455	7599	1.21 (1.13, 1.31)	1.80×10^{-7}	41.9%	0.19
TNFSF15	rs4979462	2349	2289	1.50 (1.36, 1.65)	6.86×10^{-16}	25.4%	0.247
IL12A	rs485499	1656	1924	1.17 (1.01, 1.34)	0.03	0.0%	0.601
CCR6	rs968334	10516	20772	1.16 (1.03, 1.31)	0.01	84.4%	0.011
PTPN22	rs2476601	390	711	0.75 (0.57, 0.99)	0.04	0.0%	0.461
SOCS1	rs725613	2520	4165	1.17 (1.08, 1.26)	1.10×10^{-4}	3.6%	0.308
SPIB	rs3745516	1893	3901	1.34 (1.22, 1.46)	2.29×10^{-10}	7.8%	0.298
RUNX3	rs7529070	2553	7241	1.18 (1.09, 1.28)	1.01×10^{-4}	31.4%	0.227
HLA-A	A*0201	1429	1719	1.54 (1.32, 1.80)	7.66×10^{-8}	0.0%	0.681
HLA-B	B*35	250	741	0.56 (0.37, 0.86)	8.00×10^{-3}	0.0%	0.685
HLA-DPB1	DPB1*0301	181	261	1.58 (1.02, 2.45)	0.04	0.0%	0.415

Supplementary Table 14. Allele frequency of variants in HLA region of Asia and Europe

Variants	Frequency in Asia	Frequency in Europe	Variants	Frequency in Asia	Frequency in Europe
A*3303	0.069	0.007	DRB1*08	0.071	0.033
B*4403	0.021	0.044	DRB1*0801	0.008	0.027
DPB1*0201	0.110	0.141	DRB1*0802	0.007	0.002
DPB1*0401	0.093	0.393	DRB1*0803	0.045	0.005
DPB1*0501	0.400	0.021	DRB1*11	0.053	0.138
DQA1*0102	0.200	0.175	DRB1*1101	0.045	0.077
DQA1*0401	0.015	0.028	DRB1*1201	0.034	0.017
DQB1*0301	0.193	0.220	DRB1*1202	0.143	0.005
DQB1*0401	0.041	0.006	DRB1*13	0.037	0.121
DQB1*0402	0.023	0.030	DRB1*1302	0.021	0.040
DQB1*0601	0.109	0.013	DRB1*14	0.066	0.042
DQB1*0602	0.045	0.098	DRB1*1403	0.007	<0.001
DQB1*0604	0.015	0.030	DRB1*1405	0.021	<0.001
DRB1*0405	0.054	0.014	DRB1*15	0.218	0.106
DRB1*0701	0.071	0.125	DRB1*1501	0.084	0.104

The allele frequency was obtained from Allele Frequency Net Database (http://www.allelefrequencies.net/hla6006a.asp).
